# Developing multispecies quorum-sensing modulators based on the *Streptococcus mitis* competence-stimulating peptide

**DOI:** 10.1016/j.jbc.2023.105448

**Published:** 2023-11-10

**Authors:** Tahmina A. Milly, Clay P. Renshaw, Yftah Tal-Gan

**Affiliations:** Department of Chemistry, University of Nevada, Reno, Reno, Nevada, USA

**Keywords:** quorum sensing, *Streptococcus mitis*, *Streptococcus pneumoniae*, competence stimulating peptide (CSP), Structure-activity relationships

## Abstract

Bacteria utilize quorum sensing (QS) to coordinate many group behaviors. As such, QS has attracted significant attention as a potential mean to attenuate bacterial infectivity without introducing selective pressure for resistance development. *Streptococcus mitis*, a human commensal, acts as a genetic diversity reservoir for *Streptococcus pneumoniae*, a prevalent human pathogen. *S. mitis* possesses a typical *comABCDE* competence regulon QS circuitry; however, the competence-stimulating peptide (CSP) responsible for QS activation and the regulatory role of the competence regulon QS circuitry in *S. mitis* are yet to be explored. We set out to delineate the competence regulon QS circuitry in *S. mitis*, including confirming the identity of the native CSP signal, evaluating the molecular mechanism that governs CSP interactions with histidine kinase receptor ComD leading to ComD activation, and defining the regulatory roles of the competence regulon QS circuitry in initiating various *S. mitis* phenotypes. Our analysis revealed important structure-activity relationship insights of the CSP signal and facilitated the development of novel CSP-based QS modulators. Our analysis also revealed the involvement of the competence regulon in modulating competence development and biofilm formation. Furthermore, our analysis revealed that the native *S. mitis* CSP signal can modulate QS response in *S. pneumoniae*. Capitalizing on this crosstalk, we developed a multispecies QS modulator that activates both the pneumococcus ComD receptors and the *S. mitis* ComD-2 receptor with high potencies. The novel scaffolds identified herein can be utilized to evaluate the effects temporal QS modulation has on *S. mitis* as it inhabits its natural niche.

*Streptococcus mitis* is a pioneer oral commensal species that inhabits the human mouth and is an integral member of the oral microbiome. *S. mitis* colonizes virtually all oral sites, such as dental hard tissues as well as mucous membranes, and is found in the throat and nasopharynx, where it may exist, side-by-side, in biofilms with *Streptococcus pneumoniae* (pneumococcus), a closely related major human pathogen (1, 2, 3). *S. pneumoniae* is also a nasopharyngeal commensal; however, unlike *S. mitis*, it has high pathogenic potential and is responsible for an estimated 445,000 hospitalizations and 22,000 deaths annually in the United States alone (4). Most of the deaths caused by *S. pneumoniae* are a result of bacteremia, meningitis, or pneumonia (5). Conversely, *S. mitis* presents mostly a nonvirulent behavior and is only occasionally associated with diseases (5, 6, 7, 8); however, concerns about its pathogenic potential have recently been raised (9). The differences in pathogenic potential in these two species are striking, as they share more than 80% gene similarity (1, 3). Evolutionary analyses suggest that although both species share a common ancestor, loss of virulence genes may have contributed to the less pathogenic potential in *S. mitis* (1). The ability to persistently colonize oral surfaces and the nasopharynx, as well as the ability to induce mucosal antibody responses, are unique biological features of *S. mitis* that make this commensal bacterium a potential mucosal vaccine or therapeutic delivery vehicle (10). Despite the universal prevalence of *S. mitis* as a human colonizer, very few studies have been conducted to address the molecular mechanisms involved in *S. mitis* colonization and host interactions, making it of particular interest to study (6, 11).

*S. mitis* acts as a genetic pool to competent pneumococci, including genes responsible for antibiotic resistance and virulence factor production (12). It has been postulated that the acquisition of *S. mitis* genes by *S. pneumoniae* has contributed to the survival of *S. pneumoniae* during stress conditions, suggesting that *S. mitis* plays an important role in pneumococcal evolution (13, 14). This process was found to be bidirectional, as genome analysis of six *S. mitis* strains revealed the presence of a pneumococcal-like capsule locus in four of them including in the *S. mitis* type strain CCUG 31611 (also known as NCTC 12261 or ATCC 49456) (15, 16). The exchange of serotype 4 capsule locus has been reported between the *S. mitis* type strain and *S. pneumoniae* TIGR4, following induction of competence for natural transformation (11). Natural transformation has been considered as a dominant force for the evolution of bacteria. This well-known process is used by competent streptococci to acquire genetic material from the surroundings, including genes that confer antibiotic resistance, as well as genes that regulate a wide range of functions related to pathogenicity, such as virulence factor production and biofilm formation. In both *S. mitis* and *S. pneumoniae*, competence is regulated by a density dependent quorum-sensing (QS) system, which is centered on competence pheromones, also known as competence stimulating peptides (CSPs) (17, 18, 19, 20). QS is a ubiquitous process that allows bacteria to assess their population density and alter their gene expression once they achieve high cell number (21, 22). This process is centered on the production, secretion, and detection of small diffusible signal molecules, also termed autoinducers. The concentration of these signal molecules is directly proportional to the bacterial cell density, and once the signal reaches a threshold concentration, indicative of high cell number, it activates a cognate receptor, resulting in alteration in the expression of group behavior genes (23). In many streptococci, the QS circuitry that regulates competence, termed the competence (or ComABCDE) regulon, launches the competence development cascade: first, the CSP precursor-peptide (ComC) is processed and exported to the extracellular environment by an ABC transporter (ComAB) as the mature CSP signal (24). Once CSP reaches a threshold concentration, the CSP binds and activates a membrane-bound histidine kinase receptor (ComD) (24). This binding event results in the phosphorylation of the cytoplasmic response regulator (ComE). Phosphorylated ComE acts as a transcription factor to upregulate the transcription of the QS genes (*comABCDE*) as well as the alternative sigma factor gene (*comX*), the master regulator of the QS circuitry that controls the different QS-regulated phenotypes, including competence (Fig. 1).

**Figure 1:**
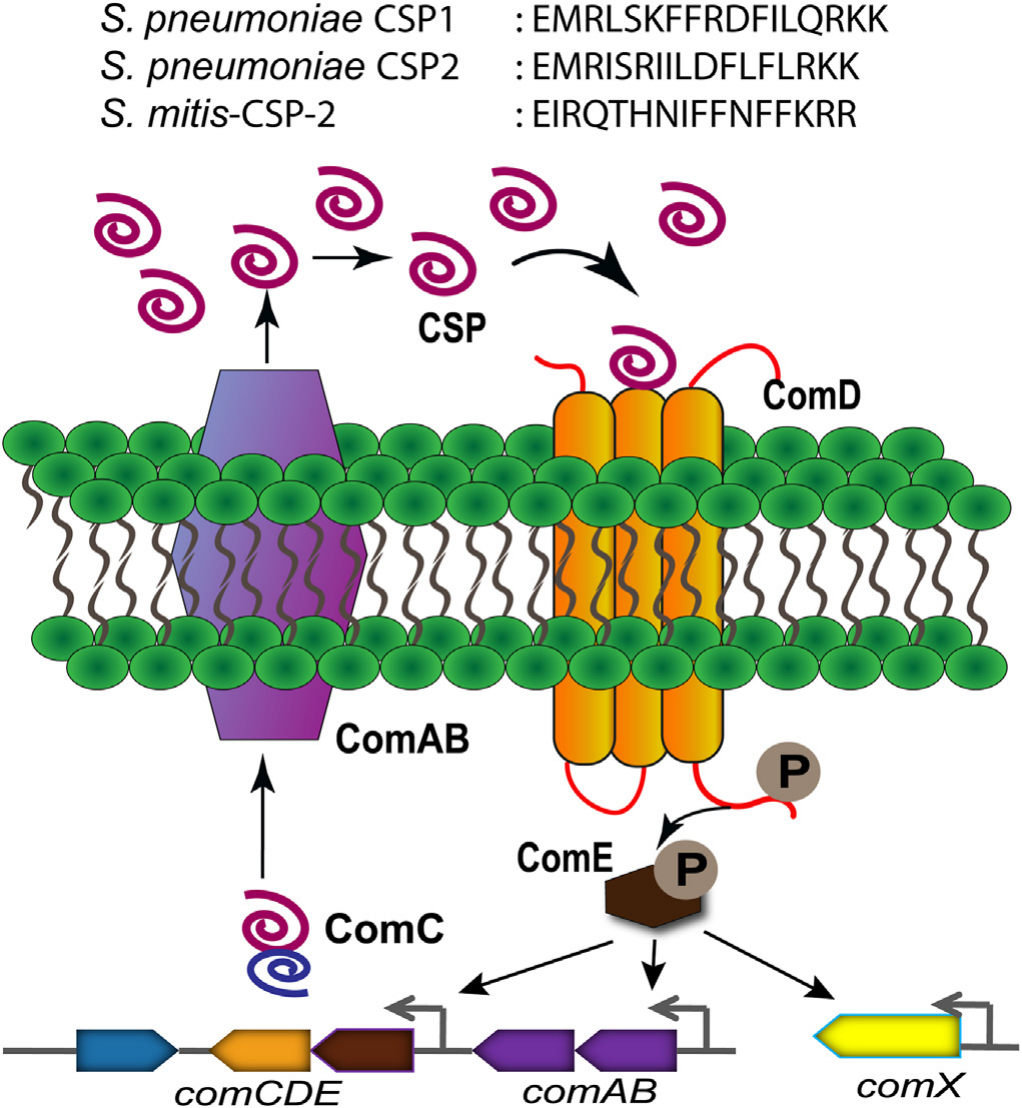
**General streptococcal CSP-mediated QS pathway.** ComC is processed and exported to the extracellular environment as the mature CSP signal by ComAB. At high concentration, the CSP binds to its cognate histidine-kinase receptor ComD. Activation of ComD leads to phosphorylation of ComE, a response regulator, resulting in further activation of the *comABCDE* operon and expression of *comX*, the master regulator of group-behavior genes. The sequences of the *Streptococcus pneumoniae* CSP1 and CSP2 as well as *Streptococcus mitis*-CSP-2 are presented at the *top*. CSP, competence-stimulating peptide; QS, quorum sensing.

Thus far, two main pherotypes, or specificity groups, were identified in *S. pneumoniae*, each utilizing a different pheromone (CSP1 or CSP2, Fig. 1) and cognate receptor (ComD1 or ComD2, respectively) (25). Similarly, due to the highly recombinogenic nature of *S. mitis*, *S. mitis* strains were predicted to produce multiple CSP variants (3, 26). Moreover, due to the structural similarity between the *S. pneumoniae* and *S. mitis* CSPs, it was postulated that *S. mitis* has the potential to influence pneumococcal QS-regulated phenotypes. Indeed, in a previous study, we have shown that a native *S. mitis* CSP pheromone, *S. mitis*-CSP-2, can effectively activate the competence regulon in both group 1 and group 2 pneumococcus (2). Capitalizing on this crosstalk and utilizing rational design, we were able to develop *S. mitis*–based CSP analogs capable of modulating the pneumococcal competence regulon at low nanomolar concentrations. This study demonstrated a complementary strategy to attenuate pneumococcal infections by using the native CSP signals of other related species. Furthermore, the privileged scaffolds identified in this study could potentially influence the *S. mitis* competence regulon, thereby providing novel chemical tools capable of modulating QS in multiple species. An additional evidence of potential crosstalk between *S. mitis* and *S. pneumoniae* has been recently reported by Junges *et al*. (27). In this study, the authors found that the Rgg (regulator gene of glucosyltransferase)/short hydrophobic peptide cell-to-cell communication system in *S. mitis* activates an Rgg/short hydrophobic peptide system in *S. pneumoniae* associated with the regulation of pneumococcal surface polysaccharide synthesis. The cross-communication between QS systems may provide important insights regarding interspecies interactions within the microbiome and between commensal and pathogenic species. However, the activity, regulation, and possible role of the *S. mitis* competence regulon in the flow of genetic information across species remain unknown, and the ability of this QS circuitry to regulate other bacterial phenotypes has yet to be explored.

The native CSP sequence for the *S. mitis* type strain was previously deduced using genetic prediction; however its identity was not confirmed through isolation from bacterial supernatants (26). Another study exhibited that with the use of this proposed synthetic 16-amino acid peptide, termed *S. mitis*-CSP-2, the challenges associated with genetically transforming *S. mitis* in the laboratory setting can be resolved (3). Furthermore, several studies reported the existence of a large variety of CSP pheromones in the mitis group of streptococci, specifically among *S. mitis* strains (3, 26). We started our study by confirming the identity of the native CSP signal for our working *S. mitis* type strain (*S. mitis* ATCC 49456) by isolating it from *S. mitis* supernatants. We then set out to characterize the regulatory role of the *S. mitis* competence regulon and to gain a deeper understanding of the molecular mechanisms that drive signal/receptor binding and subsequent activation of the QS circuitry. To this end, we first performed a full alanine and D-amino acid scans of the *S. mitis*-CSP-2 sequence to determine the structural motifs required for ComD binding and activation and developed a luciferase-based reporter gene system to measure the activity profiles of all the *S. mitis*-CSP-2 analogs. We then utilized CD spectroscopy to analyze the overall structural features of all the alanine and D-amino acid *S. mitis*-CSP-2–substituted analogs. Through the structure-function insights we gained, we designed and synthesized a library of second-generation analogs and identified a potent CSP-based inhibitor of the *S. mitis* competence regulon. We also evaluated the activities of our previously designed *S. mitis*-CSP-2–based pneumococcal QS modulators (2) against the *S. mitis* QS system and identified a highly potent multispecies *S. mitis*-CSP-2–based QS activator. Finally, we utilized RNA-Seq, along with several phenotypic assays to evaluate the role the *S. mitis* competence regulon QS circuitry plays in modulating different pathogenic phenotypes and found that this circuitry has a regulatory role in both bacterial competence and biofilm formation.

## Results

### Prediction and isolation of the *S. mitis*-CSP-2 signal from cell-free supernatants

**Figure 2:**
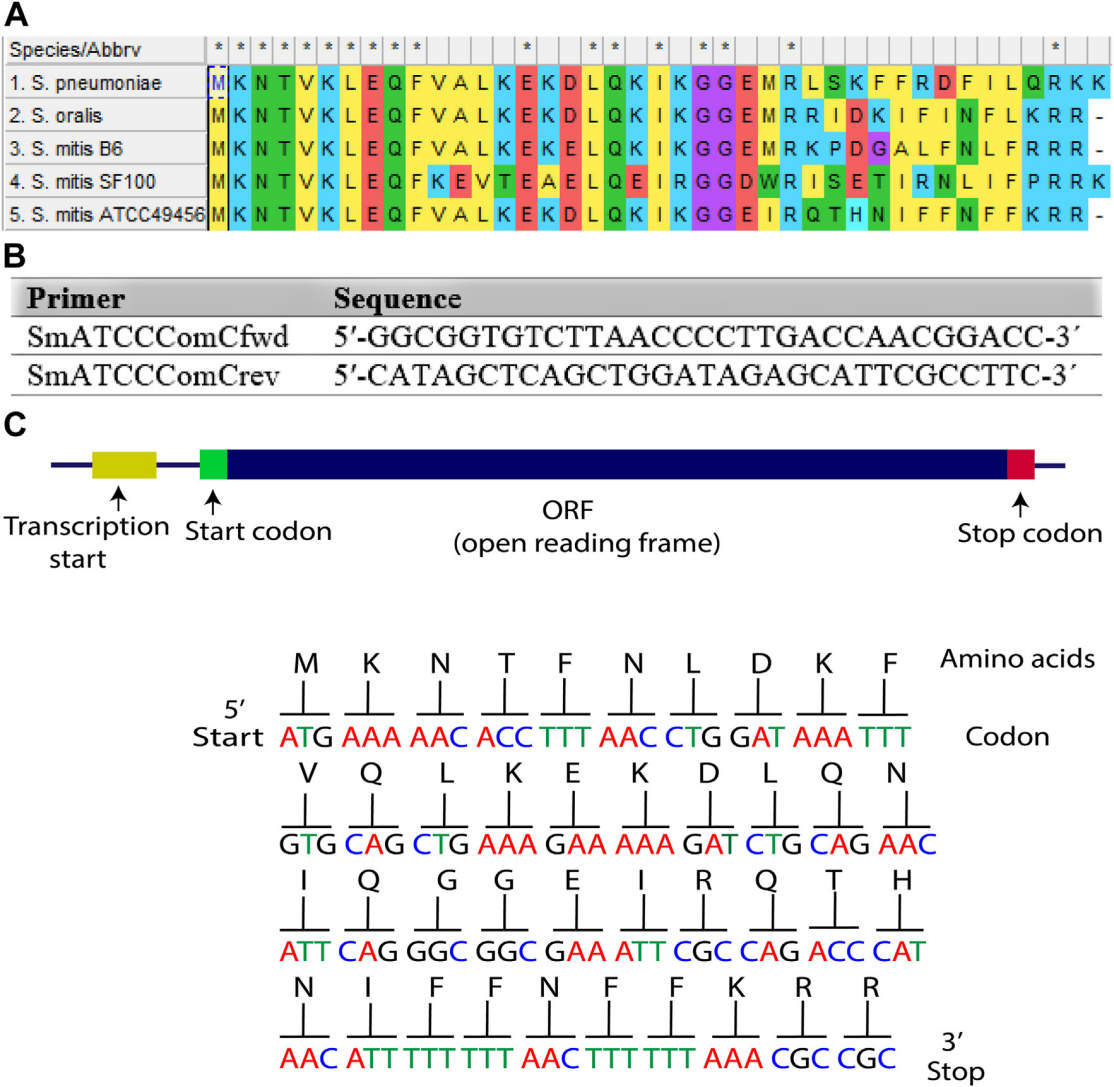
**Predicted** ***S. mitis*****-CSP-2 sequence based on sequencing results.***A*, ClustalW alignment of the *comC* gene product of several mitis group streptococci. ComC is typically cleaved at the double glycine site to afford a mature CSP signal with a negatively charged *N*-terminal residue. *B*, primers used for *comC* amplification. *C*, identification of *Streptococcus mitis*-CSP-2 following *comC* sequencing. CSP, competence-stimulating peptide.

**Figure 3:**
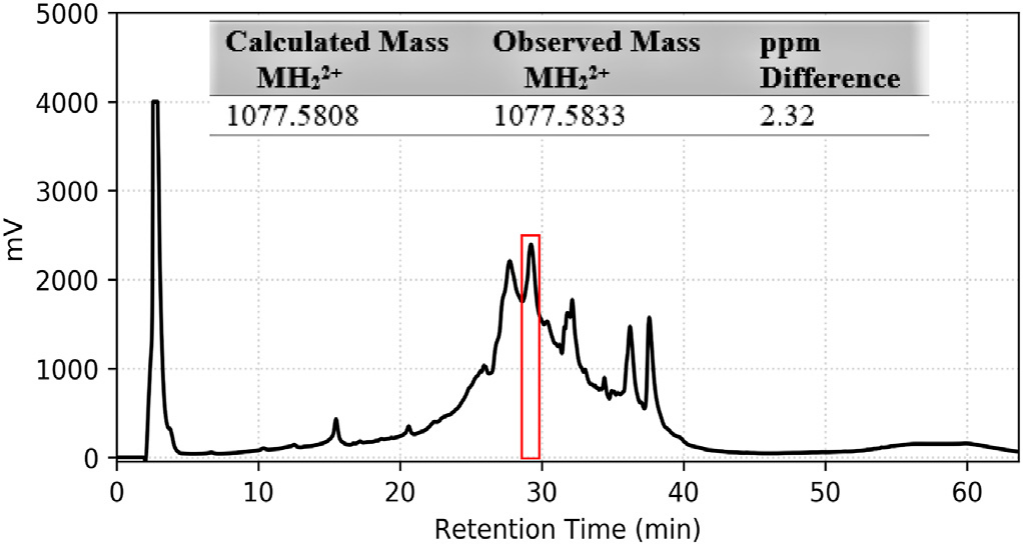
**Isolation and detection of the *Streptococcus mitis*-CSP-2 from cell-free supernatants.** The RP-HPLC chromatogram of total proteins isolated from the supernatant sample and high-resolution ESI-TOF MS of the fraction collected from 28 to 30 min (*red*). See the Supporting information for full experimental details. CSP, competence-stimulating peptide.

As previous studies reported the likelihood of multiple CSP variants in the mitis group of streptococci, particularly among *S. mitis* strains, we wanted to validate the predicted CSP sequence of our tested *S. mitis* strain (*S. mitis* ATCC 49456) (3, 26). Based on genomic data, Salvadori *et al*. have previously predicted the native CSP sequence for the *S. mitis* ATCC 49456 strain, synthesized the predicted 16-amino acid CSP with the sequence EIRQTHNIFFNFFKRR, and exhibited that this synthetic peptide can induce competence development in *S. mitis* ATCC 49456 (3). We first identified the CSP-encoding gene (*comC*) of our tested *S. mitis* strain (see Fig. 2 for primer sequences). Our sequencing results confirmed the previously reported predicted 16-amino acid peptide sequence (Fig. 2). Most often, after translation of the PCR product, the deduced mature CSP sequence is preceded by a double glycine cleavage site. Previously, several studies have reported that to afford a mature CSP signal, ComC sometimes undergoes additional processing, either through further processing by the membrane-bound ComAB transporter or through cleavage of the exported CSP signal with the help of an extracellular SepM protease (26, 28, 29). We therefore sought to validate the identity of the mature *S. mitis* CSP by isolating the processed CSP from bacterial supernatants. Following ammonium sulfate precipitation of the excreted crude peptide mixture from cell-free *S. mitis* supernatants, we used semipreparative RP-HPLC to fractionate the total crude mixture and observed exact masses similar to those of the predicted CSP sequence (Fig. 3).

### Comparison of isolated and synthetic *S. mitis*-CSP-2

**Figure 4:**
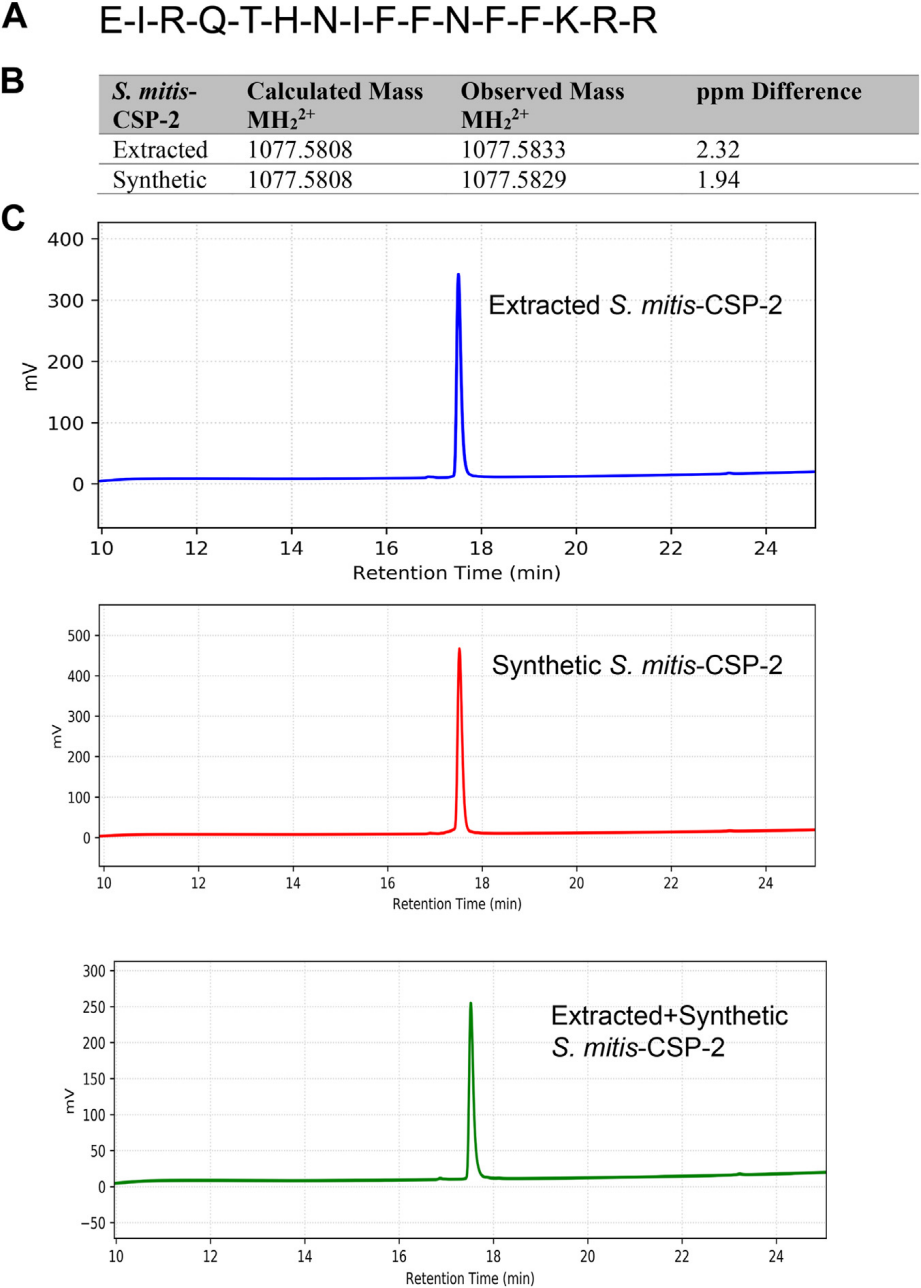
**Comparison of purified synthetic and isolated *Streptococcus mitis*-CSP-2.***A*, proposed structure of the 16-amino acid *S. mitis*-CSP-2. *B*, comparison of observed masses of isolated and synthetic peptides by ESI-TOF MS. *C*, comparison of analytical RP-HPLC chromatograms of the purified isolated, synthetic, and isolated and synthetic *S. mitis*-CSP-2. CSP, competence-stimulating peptide.

To further validate the identity of the *S. mitis* native CSP signal, the predicted CSP was synthesized (for full synthetic details, see the Supporting information). We then compared the synthetic purified peptide to the isolated purified peptide obtained from *S. mitis* cell-free supernatants. Analytical HPLC analysis of both individual peptides revealed a dominant peak with the same retention time, and a combined fraction of the synthetic and isolated peptides in a 1:1 ratio resulted in a single peak that possessed the same retention time as the individual fractions (Fig. 4). The exact masses of the isolated and synthetic peptides were obtained and matched the expected exact mass of the 16 amino acid CSP sequence (Fig. 4). Furthermore, MS/MS analysis of the isolated and synthetic peptides validated the connectivity of the CSP sequence (Figs. S22–S24). Overall, our results confirmed the native *S. mitis* CSP sequence, which is the 16-amino acid peptide, EIRQTHNIFFNFFKRR.

### Development of *S. mitis* luciferase QS reporter strain

A luciferase-based *S. mitis* ATCC 49456 QS reporter strain was constructed to test the ability of the native *S. mitis*-CSP-2 and *S. mitis*-CSP-2 analogs to modulate the *S. mitis* ComD-2 receptor activity. We utilized a similar approach performed by Salvadori *et al*. (3) to construct a luciferase-based *S. mitis* reporter strain to directly quantify the degree of *comX* expression upon CSP/ComD binding. Successful construction of the reporter strain (ATCC_49456_ P*comX* luc::Spc) was verified by sequencing as well as by observing an increase in luminescence following treatment of *S. mitis* reporter culture with the native *S. mitis*-CSP-2 signal and 15 μg/ml D-luciferin, compared to a control untreated with the *S. mitis*-CSP-2 (see the Supporting information for full experimental details).

### Design and synthesis of first-generation *S. mitis*-CSP-2 analogs

To quantitatively evaluate the contribution of each side chain and chiral center of the 16-amino acid *S. mitis*-CSP-2, we conducted full alanine and D-amino acid scans of the *S. mitis*-CSP-2 sequence. The activity of each synthesized *S. mitis*-CSP-2 analog was determined through a luciferase reporter gene assay using the *S. mitis* luciferase QS reporter strain we constructed (see Experimental procedures for protocol details).

### Structure-activity relationship analysis of the *S. mitis*-CSP-2 alanine-screen analogs

We first quantified the ability of the native *S. mitis*-CSP-2 to activate its cognate ComD receptor through determination of its EC_50_ value (half maximal effective concentration). Initial bioassays revealed that *S. mitis*-CSP-2 activates *S. mitis* ComD-2 with an EC_50_ value of 148 nM (Table 1). All the *S. mitis*-CSP-2 analogs were then initially screened, at high analog concentration (10,000 nM), for their ability to activate the *S. mitis* ComD-2 receptor to a level comparable to the native *S. mitis*-CSP-2 signal (see Fig. S1). *S. mitis*-CSP-2 analogs that revealed high receptor activation, as determined in the initial screening (>75% activation compared to *S. mitis*-CSP-2), were further assessed to determine their EC_50_ values, while *S. mitis*-CSP-2 analogs that failed to activate the receptor (<50% activation compared to *S. mitis*-CSP-2) were assessed for their ability to competitively inhibit the receptor (see Fig. S8).

**Table 1:** EC_50_ or IC_50_ values of *Streptococcus mitis*-CSP-2 alanine-screen analogs against the *S. mitis* ComD-2 receptor^a^

Peptide name	Peptide sequence	EC_50_ or IC_50_∗ (nM)^b^	95% CI^c^
*S. mitis-*CSP-2	EIRQTHNIFFNFFKRR	148	86.0–255
*S. mitis-*CSP-2-E1A	**A**IRQTHNIFFNFFKRR	456**∗**	338–617
*S. mitis-*CSP-2-I2A	E**A**RQTHNIFFNFFKRR	---d	---
*S. mitis-*CSP-2-R3A	EI**A**QTHNIFFNFFKRR	---d	---
*S. mitis-*CSP-2-Q4A	EIR**A**THNIFFNFFKRR	227	210–246
*S. mitis*-CSP-2-T5A	EIRQ**A**HNIFFNFFKRR	178	108–292
*S. mitis*-CSP-2-H6A	EIRQT**A**NIFFNFFKRR	>1000	---
*S. mitis*-CSP-2-N7A	EIRQTH**A**IFFNFFKRR	114	74.3–175
*S. mitis*-CSP-2-I8A	EIRQTHN**A**FFNFFKRR	605	558–655
*S. mitis*-CSP-2-F9A	EIRQTHNI**A**FNFFKRR	>1000	---
*S. mitis*-CSP-2-F10A	EIRQTHNIF**A**NFFKRR	579	497–675
*S. mitis*-CSP-2-N11A	EIRQTHNIFF**A**FFKRR	46.3	41.0–53.0
*S. mitis*-CSP-2-F12A	EIRQTHNIFFN**A**FKRR	121	92.4–158
*S. mitis*-CSP-2-F13A	EIRQTHNIFFNF**A**KRR	142	112–180
*S. mitis*-CSP-2-K14A	EIRQTHNIFFNFF**A**RR	163	143–187
*S. mitis*-CSP-2-R15A	EIRQTHNIFFNFFK**A**R	303	248–370
*S. mitis*-CSP-2-R16A	EIRQTHNIFFNFFKR**A**	251	184–344

Modified residues in the CSP sequence are bolded.

a See the ‘Experimental procedures’ section for experimental details and the Supporting information for details of the reporter strain and plots of agonism or antagonism dose-response curves. All assays were performed in triplicate.

b EC_50_ or IC_50_ values were determined by testing peptides over a wide range of concentrations.

c 95% confidence interval.

d EC_50_ not determined due to the analog’s low induction in primary agonism screening assay. See the Supporting information for details.

The reporter gene data revealed several interesting structure-activity relationship (SAR) trends regarding *S. mitis*-CSP-2:ComD-2 binding and consequently ComD-2 activation. The *S. mitis*-CSP-2 can be divided into three distinct regions, the *N* terminus (first three amino acid residues), the central region (residues 4–13), and the *C* terminus (last three amino acid residues, residues 14–16). Alanine screening of the *S. mitis*-CSP-2 *N* terminus revealed that the first three residues (Glu1, Ile2, and Arg3) have a critical role in *S. mitis* ComD-2 receptor binding and activation. Alanine replacements of Ile2 and Arg3 resulted in a complete loss of activity, whereas alanine replacement of Glu1 resulted in the conversion of the *S. mitis*-CSP-2 into a ComD-2 inhibitor (Table 1). These results suggest that the *N* terminus of *S. mitis*-CSP-2 participates in very specific binding interactions that cannot withstand significant side-chain modifications. Our results of the *N*-terminus region of *S. mitis*-CSP-2 are consistent with what was previously observed for CSPs produced by other members of the mitis group of streptococci, such as *S. pneumoniae* and *Streptococcus oligofermentans*, where the first negatively charged amino acid (Glu or Asp) was found to play a critical role in receptor activation while the third residue (Arg) was found to play a critical role in receptor binding, suggesting the presence of highly conserved motifs at these two positions (29, 30).

In the *S. mitis*-CSP-2 core region, the alanine scan results revealed that changes in residues H6, I8, F9, and F10 resulted in a significant reduction in potency, while modification in residues Q4, T5, N7, N11, F12, and F13 resulted in analogs with similar activities to the native CSP (Table 1). Specifically, the alanine substitution results suggest that the H6 and F9 side chains are either critical for receptor binding or are key to stabilizing the peptide bioactive conformation. Thus, modifications to either one of these residues resulted in the most significant loss in potency (Table 1). In contrast, alanine replacement of N11 resulted in an analog with increased potency (EC_50_ = 46.3 nM) compared to the native signal (EC_50_ = 148 nM), suggesting that the removal of this side chain either directly improved the binding interactions with the ComD-2 receptor (likely through elimination of electrostatic or steric clashes) or allowed the peptide to assume a more favorable conformation for receptor binding.

As for the *S. mitis*-CSP-2 *C*-terminal region (residues K14, R15, and R16), alanine mutation resulted in analogs with similar activities to the parent *S. mitis*-CSP-2 signal (Table 1), suggesting that these residues are not critical for receptor binding or activation. These findings are in agreement with the results obtained for CSPs produced by other members of the mitis group of streptococci (29, 30). Yet, since these three residues are conserved in most mitis group of streptococci CSPs, it is likely that these residues have an important evolutionary role in the competence regulon QS circuitry. Indeed, it was hypothesized that these three positively charged residues increase the solubility of these overall relatively hydrophobic peptide sequences, thereby facilitating their departure from the cell membrane to the extracellular environment following export.

### SAR analysis of the *S. mitis*-CSP-2 D-amino acid scan analogs

Similarly to the *S. mitis*-CSP-2 *N*-terminus alanine substitutions, examination of the D-amino acid scan of the *S. mitis*-CSP-2 *N*-terminus further confirmed the importance of the first three residues in ComD-2 receptor binding and activation. Alanine and D-amino acid replacement of Ile2 and Arg3 resulted in complete loss of activity. However, in the case of Glu1, while alanine substitution resulted in the conversion of *S. mitis*-CSP-2 into a potent ComD-2 inhibitor, D-amino acid replacement completely abolished the peptide activity (see both Tables 1 and 2). These results suggest that the *N* terminus of the *S. mitis*-CSP-2 cannot accommodate chirality alterations, likely due to the inability of the differently oriented side chains to fit into their respective receptor binding pockets.

**Table 2:** EC_50_ values of *Streptococcus mitis*-CSP-2 D-amino acid scan analogs against the *S. mitis* ComD-2 receptor^a^

Peptide name	Peptide sequence	EC_50_(nM)^b^	95% CI^c^
*S. mitis-*CSP-2	EIRQTHNIFFNFFKRR	148	86.0–255
*S. mitis-*CSP-2-e1	**e**IRQTHNIFFNFFKRR	---d	---
*S. mitis-*CSP-2-i2	E**i**RQTHNIFFNFFKRR	---d	---
*S. mitis-*CSP-2-r3	EI**r**QTHNIFFNFFKRR	---d	---
*S. mitis-*CSP-2-q4	EIR**q**THNIFFNFFKRR	>1000	---
*S. mitis*-CSP-2-t5	EIRQ**t**HNIFFNFFKRR	>1000	---
*S. mitis*-CSP-2-h6	EIRQT**h**NIFFNFFKRR	>1000	---
*S. mitis*-CSP-2-n7	EIRQTH**n**IFFNFFKRR	>1000	---
*S. mitis*-CSP-2-i8	EIRQTHN**i**FFNFFKRR	90.0	49.0–165
*S. mitis*-CSP-2-f9	EIRQTHNI**f**FNFFKRR	438	328–583
*S. mitis*-CSP-2-f10	EIRQTHNIF**f**NFFKRR	64.0	42.2–96.4
*S. mitis*-CSP-2-n11	EIRQTHNIFF**n**FFKRR	38.3	22.4–66.0
*S. mitis*-CSP-2-f12	EIRQTHNIFFN**f**FKRR	245	152–394
*S. mitis*-CSP-2-f13	EIRQTHNIFFNF**f**KRR	338	155–735
*S. mitis*-CSP-2-k14	EIRQTHNIFFNFF**k**RR	95.0	47.3–189
*S. mitis*-CSP-2-r15	EIRQTHNIFFNFFK**r**R	143	73.1–278
*S. mitis*-CSP-2-r16	EIRQTHNIFFNFFKR**r**	64.0	59.0–69.0

Modified residues in the CSP sequence are bolded.

a See the ‘Experimental procedures’ section for experimental details and the Supporting information for details of the reporter strain and plots of agonism or antagonism dose-response curves. All assays were performed in triplicate.

b EC_50_ values were determined by testing peptides over a wide range of concentrations.

c 95% confidence interval.

d EC_50_ not determined due to the analog’s low induction in primary agonism screening assay. See the Supporting information for details.

Looking at the hydrophilic residues at the central region of *S. mitis*-CSP-2 (Gln4, Thr5, His6, Asn7, and Asn11, Table 2), it appears that side chain orientation changes are less tolerated than alanine mutations, as, with the exception of Asn11, D-amino acid substitution analogs exhibited significant loss of activity. Thus, we concluded that in these cases, the side chain orientation and concomitantly induced local peptide conformation is more important than the actual side chain residue. Interestingly, like the alanine substitution, the D-amino acid replacement of Asn11 resulted in an analog with improved potency (EC_50_ = 38.3 nM, Table 2), producing the most potent analog in these two libraries. Since position 11 of *S. mitis*-CSP-2 tolerates well both chirality and side chain changes, it is likely not directly participating in important receptor-binding interactions.

Our D-amino acid scan analysis of the hydrophobic residues at the core region of *S. mitis*-CSP-2 (I8, F9, F10, F12, and F13) revealed an opposite trend than the one for the hydrophilic residues. That is, in the case of the hydrophobic residues, the side chain identity is more important than the actual side chain orientation, as most of the D-amino acid substitutions in these positions yielded analogs with similar or only slightly reduced activities compared to the native *S. mitis*-CSP-2 signal, whereas most alanine modifications in these positions resulted in significant reduction in potency (see both Tables 1 and 2). The results of both the alanine and D-amino acid replacements suggest that the Phe12 and Phe13 residues do not play an important role in *S. mitis* ComD-2 receptor binding.

Lastly, chirality changes at the *C*-terminal residues of *S. mitis*-CSP-2 confirmed that these three positions (Lys14, Arg15, and Arg16) are highly modifiable, as D-amino acid substitutions resulted in analogs with similar (Arg15) or even better activities (Lys14 and Arg16) compared to the native *S. mitis*-CSP-2 signal.

### Second-generation CSP analogs

Our initial alanine and D-amino acid substitutions analysis revealed several interesting activity trends. For instance, our alanine scan analysis revealed that alanine replacements of Asn7, Asn11, Phe12, and Phe13 resulted in analogs with improved activities compared to the native peptide (Table 1). Furthermore, replacement of the negatively charged glutamic acid at position 1 with alanine resulted in an analog that exhibits competitive inhibition against the *S. mitis* ComD-2 receptor (Table 1), a trend consistent with CSPs produced by other mitis group streptococci (29, 30). Similarly, the D-amino acid scan revealed that replacements of Ile8, Phe10, Asn11, Lys14, and Arg16 with their enantiomers resulted in analogs with enhanced activities compared to native *S. mitis*-CSP-2 (Table 2). Therefore, to further explore these trends and identify enhanced CSP-based QS modulators of the *S. mitis* competence regulon, we designed and synthesized a second-generation library of *S. mitis*-CSP-2 analogs that included both sequential truncations of either the three *N*-terminal residues or the three *C*-terminal residues of the native CSP signal (Table 3), as well as multiple-modification analogs combining the E1A modification with the modifications that led to enhanced activators (Table 4).

**Table 3:** EC_50_ or IC_50_ values of *Streptococcus mitis*-CSP-2 truncated analogs against the *S. mitis* ComD-2 receptor^a^

Peptide name	Peptide sequence	EC_50_ or IC_50_∗(nM)^b^	95% CI^c^
*S. mitis-*CSP-2	EIRQTHNIFFNFFKRR	148	86.0–255
*S. mitis-*CSP-2-des-E1	IRQTHNIFFNFFKRR	>1000∗	---
*S. mitis-*CSP-2-des-E1I2	RQTHNIFFNFFKRR	---d	---
*S. mitis-*CSP-2- des-E1I2R3	QTHNIFFNFFKRR	---d	---
*S. mitis*-CSP-2-des-R16	EIRQTHNIFFNFFKR	39.2	21.4–72.0
*S. mitis*-CSP-2-des-R15R16	EIRQTHNIFFNFFK	242	185–315
*S. mitis-*CSP-2-des-K14R15R16	EIRQTHNIFFNFF	---d	---

a See the ‘Experimental procedures’ section for experimental details and the Supporting information for details of the reporter strain and plots of agonism or antagonism dose-response curves. All assays were performed in triplicate.

b EC50 or IC50 values were determined by testing peptides over a wide range of concentrations.

c 95% confidence interval.

d EC50 not determined due to the analog’s low induction in primary agonism screening assay. See the Supporting information for details.

**Table 4:** IC_50_ values of *Streptococcus mitis*-CSP-2-E1A modification analogs against the *S. mitis* ComD-2 receptor^a^

Peptide name	Peptide sequence	IC_50_(nM)^b^	95% CI^c^
*S. mitis-*CSP-2-E1A	**A**IRQTHNIFFNFFKRR	456	338–617
*S. mitis*-CSP-2-E1AN7A	**A**IRQTH**A**IFFNFFKRR	242	139–422
*S. mitis*-CSP-2-E1AN11A	**A**IRQTHNIFF**A**FFKRR	513	322–819
*S. mitis*-CSP-2-E1AF12A	**A**IRQTHNIFFN**A**FKRR	>1000	---
*S. mitis*-CSP-2-E1AF13A	**A**IRQTHNIFFNF**A**KRR	>1000	---
*S. mitis*-CSP-2-E1Ai8	**A**IRQTHN**i**FFNFFKRR	---d	---
*S. mitis*-CSP-2-E1Af10	**A**IRQTHNIF**f**NFFKRR	372	215–643
*S. mitis*-CSP-2-E1An11	**A**IRQTHNIFF**n**FFKRR	258	121–548
*S. mitis*-CSP-2-E1Ak14	**A**IRQTHNIFFNFF**k**RR	159	106–238
*S. mitis*-CSP-2-E1Ar16	**A**IRQTHNIFFNFFKR**r**	236	147–380
*S. mitis*-CSP-2-E1A-des-R16	**A**IRQTHNIFFNFFKR	347	208–579
*S. mitis*-CSP-2-E1AN7Af10	**A**IRQTH**A**IF**f**NFFKRR	172	123–240
*S. mitis*-CSP-2-E1AN7An11	**A**IRQTH**A**IFF**n**FFKRR	197	127–306
*S. mitis*-CSP-2-E1AN7Ak14	**A**IRQTH**A**IFFNFF**k**RR	440	301–642
*S. mitis*-CSP-2-E1AN7Ar16	**A**IRQTH**A**IFFNFFKR**r**	290	151–555
*S. mitis*-CSP-2-E1Af10n11	**A**IRQTHNIF**fn**FFKRR	>1000	---
*S. mitis*-CSP-2-E1Af10k14	**A**IRQTHNIF**f**NFF**k**RR	521	380–715
*S. mitis*-CSP-2-E1Af10r16	**A**IRQTHNIF**f**NFFKR**r**	87.3	50.0–154
*S. mitis*-CSP-2-E1An11k14	**A**IRQTHNIFF**n**FF**k**RR	248	148–416
*S. mitis*-CSP-2-E1An11r16	**A**IRQTHNIFF**n**FFKR**r**	238	135–420
*S. mitis*-CSP-2-E1Ak14r16	**A**IRQTHNIFFNFF**k**R**r**	661	537–814

Modified residues in the CSP sequence are bolded.

a See the ‘Experimental procedures’ section for experimental details and the Supporting information for details of the reporter strain and plots of antagonism dose-response curves. All assays were performed in triplicate.

b IC_50_ values were determined by testing peptides over a wide range of concentrations.

c 95% confidence interval.

d IC_50_ not determined due to the analog’s low activity in primary antagonism screening assay. See the Supporting information for details.

**Figure 5:**
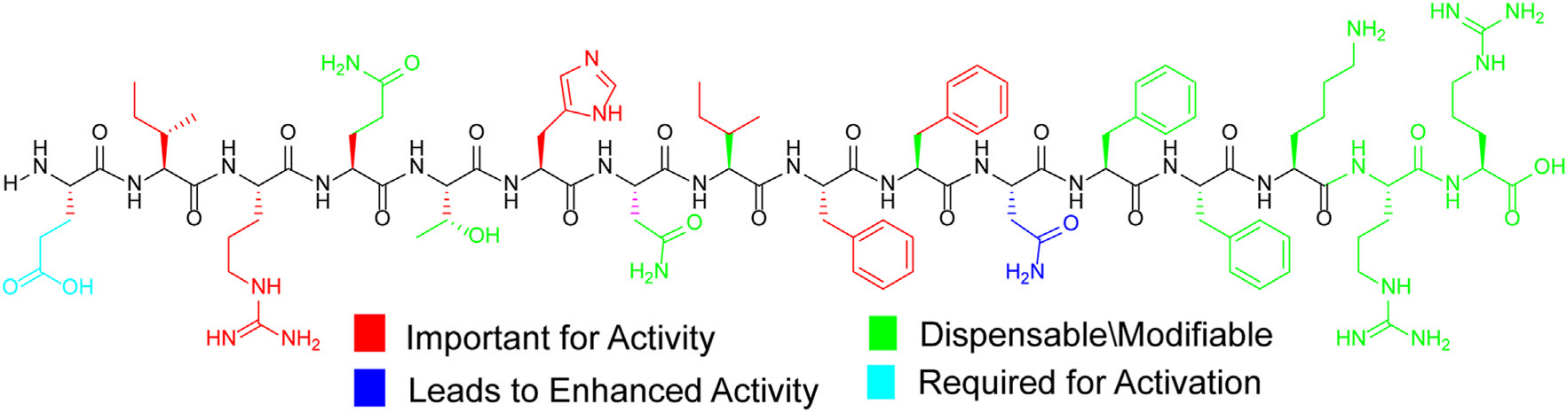
**Structure of *Streptococcus mitis*-CSP-2 highlighting the SAR trends observed through**Table 1, Table 2, Table 3. CSP, competence-stimulating peptide; SAR, structure-activity relationship.

Truncation of the first amino acid at the peptide *N* terminus resulted in a weak QS inhibitor (*S. mitis*-CSP-2-des-E1, IC_50_ > 1000 nM, Table 3). Removal of any additional residue from the *N* terminus resulted in complete loss of activity, highlighting the importance of the *S. mitis*-CSP-2 *N* terminus in both receptor binding and activation. As for the *C* terminus, removal of the last *C*-terminal residue produced a peptide with a four-fold enhanced activity compared to the native peptide (compare the EC_50_ value of *S. mitis*-CSP-2-des-R16, 39.2 nM with that of *S. mitis*-CSP-2, 148 nM, Table 3). Furthermore, truncation of the last two residues at the *C* terminus resulted in an analog with comparable activity to the native signal. Together, these results suggest that the last two *C*-terminal residues within the *S. mitis*-CSP-2 scaffold do not play a major role in the peptide activity. Contrary, truncation of three residues at the *C* terminus, to afford *S. mitis*-CSP-2-des-K14R15R16, has led to a complete loss of activity (Table 3), revealing the minimal sequence required for effective *S. mitis* ComD-2 binding. Of note, truncation of either two or three *C*-terminal residues produced analogs that were difficult to dissolve in aqueous solutions, supporting the hypothesis that the purpose of the positively charged *S. mitis*-CSP-2 *C* terminus is to enhance peptide solubility. Figure 5 summarizes the SAR trends observed through Table 1, Table 2, Table 3.

Biological evaluation of the four double-alanine mutation analogs, *S. mitis*-CSP-2-E1AN7A, *S. mitis*-CSP-2-E1AN11A, *S. mitis*-CSP-2-E1AF12A, and *S. mitis*-CSP-2-E1AF13A, revealed four *S. mitis* ComD-2 inhibitors (Table 4). However, when comparing the bioactivities of the resulting double mutation inhibitors with the corresponding initial single mutation analogs, it appears that there is no correlation between EC_50_ and IC_50_ values. Although alanine replacement of all these residues, with the exception of the E1A modification, yielded potent *S. mitis* ComD-2 activators (Table 1), only the double mutant bearing the N7A modification combined with the E1A modification generated a more potent *S. mitis* ComD-2 inhibitor (IC_50_ = 242 nM) compared to the E1A substitution alone (IC_50_ = 456 nM). The remaining three analogs were only weak inhibitors (Table 4).

A different trend was observed for the double mutation analogs generated from the combination of the potent activators revealed through the D-amino acid replacement and the E1A mutation. In this case, all the resultant analogs, with the exception of *S. mitis*-CSP-2-E1Ai8, exhibited *S. mitis* ComD-2 inhibition with higher potency than the E1A substitution alone (Table 4). Interestingly, D-amino acid replacement of residue Ile8 displayed two-fold higher agonistic activity than the native signal (Table 2), whereas the combination with the E1A substitution resulted in an analog with complete loss of activity. Overall, our results suggest that there are different requirements for receptor inhibition compared to receptor activation. These results are consistent with results obtained for CSPs produced by other members of the mitis group of streptococci, demonstrating that direct conversion of potent activators into potent inhibitors is not necessarily straightforward and requires additional fine-tuning (18).

Our sequential truncation analysis revealed that removal of R16 produced a peptide that exhibited a 4-fold enhanced activity compared to that of the native peptide (Table 3), suggesting that this side chain residue may in fact cause some steric clashes that reduces the potency of the native CSP. Thus, we incorporated this truncation together with the E1A modification to afford *S. mitis*-CSP-2-E1A-des-R16 (Table 4). This analog exhibited similar *S. mitis* ComD-2 inhibitory activity to that of the analog containing only the E1A modification, suggesting that for *S. mitis* ComD-2 inhibition, the presence of this side chain residue is not detrimental and can thus be incorporated for the design of potent inhibitors of the *S. mitis* competence regulon.

To produce enhanced inhibitors of the competence regulon, a library of tri-substituted analogs was designed and constructed. To this end, the E1A modification was combined with two of the following modifications: alanine replacement of position 7 or D-amino acid replacement at positions 10, 11, 14, or 16, which were found to be the most beneficial in the doubly-substitution library (Table 4). As expected, all the tri-substituted analogs exhibited *S. mitis* ComD-2 inhibitory activity. Analogs containing the E1A modification together with the N7A substitution and D-amino acid replacement of either position 10, 11, or 16 resulted in three analogs, *S. mitis*-CSP-2-E1AN7Af10, *S. mitis*-CSP-2-E1AN7An11, and *S. mitis*-CSP-2-E1AN7Ar16, that exhibited a 2- to 3-fold improvement in inhibitory potency compared to the E1A substitution alone, whereas the combination of the E1A, N7A, and D-amino acid substitution at position 14 produced an analog, *S. mitis*-CSP-2-E1AN7Ak14, that exhibited comparable activity to that of the E1A substitution alone (Table 4). The combination of D-amino acid substitution at position Phe10 and D-amino acid in either Asn11 or Lys14, together with the E1A modification, resulted in significant decrease in inhibitory potency (*S. mitis*-CSP-2-E1Af10n11, IC_50_ > 1000 nM and *S. mitis*-CSP-2-E1Af10k14, IC_50_ = 521 nM; Table 4). Contrary, the combination of D-amino acid at positions Phe10 and Arg16, together with the E1A modification, resulted in an analog, *S. mitis*-CSP-2-E1Af10r16, that exhibited a 5-fold increase in potency compared to the E1A modification alone, yielding the most potent inhibitor identified in this study (IC_50_ = 87.3 nM, Table 4). Overall, several potent *S. mitis* ComD-2 inhibitors were discovered through the multiple mutation library.

### Structural analysis using CD spectroscopy

Though our SAR analysis of the *S. mitis*-CSP-2 revealed the importance of several residues in receptor binding or activation, this analysis was not enough to explain how these different modifications affected the overall peptide structure. To gain insight as to how the different modifications affected the overall peptide structure and correlate it to bioactivity, we set out to assess the structures of the native *S. mitis*-CSP-2 pheromone and its alanine and D-amino acid scan analogs using CD spectroscopy. We assessed all the synthetic *S. mitis*-CSP-2 analogs under both aqueous (PBS buffer, pH 7.4) (Figs. S14–S17) and membrane-mimicking conditions (20% trifluoroethanol in PBS, pH 7.4) (Figs. S18–S21). In both aqueous and membrane-mimicking conditions, the native *S. mitis*-CSP-2 was found to be unstructured, exhibiting a random coil characteristic. Like the native pheromone, most of the alanine and D-amino acid scan analogs were found to be unstructured, exhibiting a random coil pattern in both aqueous and membrane-mimicking conditions, with the exception of a few analogs that exhibited some β-sheet pattern. These results were surprising; as for CSPs produced by other members of the mitis group of streptococci, including *S. pneumoniae* and *S. oligofermentans*, an α-helix conformation was proposed to be important for biological activity (29, 30). Overall, a correlation between a specific structural motif and biological activity could not be determined in the case of *S. mitis*-CSP-2.

### Identifying *S. mitis*-CSP-2–based multispecies QS modulators

In a previous study, we sought to uncover potential crosstalk between streptococci species by evaluating the ability of native streptococci CSPs to modulate the *S. pneumoniae* competence regulon (2). Our analysis revealed a potential role of *S. mitis* in modulating QS in *S. pneumoniae*, as the native *S. mitis*-CSP-2 pheromone was found to activate both pneumococcal ComD receptors (ComD1 and ComD2). We therefore sought to evaluate whether the same native signals from closely related streptococci species, which we already tested against *S. pneumoniae*, namely CSP signals produced by members of the mitis and anginosus groups of streptococci, are able to modulate the *S. mitis* competence regulon. Our cell-based luciferase assay revealed that only one native signal produced by *S. pneumoniae*, termed CSP1, is capable of activating the *S. mitis* ComD-2 receptor, albeit at high peptide concentration (EC_50_ = 3020 nM, Table 5; see the Supporting information for initial screening).

**Table 5:** EC_50_ or IC_50_ values of select *Streptococcus mitis*-CSP-2–based pneumococcal QS modulators against the pneumococcus ComD1 and ComD2 and the *S. mitis* ComD-2 receptors^a^

Peptide name	Peptide sequence	*Streptococcus mitis*		*Streptococcus pneumoniae*^b^	
		ComD-2		ComD1	ComD2
		EC_50_ or IC_50_∗ (nM)^c^	95% CI^d^	EC_50_ or IC_50_∗ (nM)	EC_50_ or IC_50_∗ (nM)
*S. mitis*-CSP-2	EIRQTHNIFFNFFKRR	148	86.0–255	663	635
*S. pneumoniae*-CSP1	EMRLSKFFRDFILQRKK	3020	1550–5870	10.3	526
*S. pneumoniae*-CSP2	EMRISRIILDFLFLRKK	---	---	1650	50.7
*S. mitis*-CSP-2-I2M	E**M**RQTHNIFFNFFKRR	591	385–908	87.7	136
*S. mitis*-CSP-2-Q4L	EIR**L**THNIFFNFFKRR	54.2	25.0–118	88.2	252
*S. mitis*-CSP-2-I8F	EIRQTHN**F**FFNFFKRR	470	234–945	128	209
*S. mitis*-CSP-2-N11F	EIRQTHNIFF**F**FFKRR	47.1	22.0–102	4.63	220
*S. mitis*-CSP-2-N7I	EIRQTH**I**IFFNFFKRR	105	57.0-194	101	211
*S. mitis*-CSP-2-I2MQ4L	E**M**R**L**THNIFFNFFKRR	332	212–522	49.9	141
*S. mitis*-CSP-2-I2MI8F	E**M**RQTHN**F**FFNFFKRR	---e	---	151	83.2
*S. mitis*-CSP-2-I2MF12L	E**M**RQTHNIFFN**L**FKRR	738	475–1150	25.8	146
*S. mitis*-CSP-2-Q4LF12L	EIR**L**THNIFFN**L**FKRR	201	129–315	68.7	202
*S. mitis*-CSP-2-N7II8F	EIRQTH**IF**FFNFFKRR	98.3	59.2–163	87.2	22.8
*S. mitis*-CSP-2-N11FF12L	EIRQTHNIFF**FL**FKRR	289	202–415	4.97	127
*S. mitis*-CSP-2-I2MQ4LN7F	E**M**R**L**TH**F**IFFNFFKRR	>1000	---	137	75.6
*S. mitis*-CSP-2-I2MI8FN11F	E**M**RQTHN**F**FF**F**FFKRR	---e	---	6.95	26.2
*S. mitis*-CSP-2-I2MN7FF12L	E**M**RQTH**F**IFFN**L**FKRR	>1000	---	72.8	112
*S. mitis*-CSP-2-I2MN7II8F	E**M**RQTH**IF**FFNFFKRR	>1000	---	61.6	2.67
*S. mitis*-CSP-2-I2MQ4LF12L	E**M**R**L**THNIFFN**L**FKRR	878	688–1120	17.1	139
*S. mitis*-CSP-2-I2MQ4LI8F	E**M**R**L**THN**F**FFNFFKRR	---e	---	42.0	30.0
*S. mitis*-CSP-2-I2MQ4LN11F	E**M**R**L**THNIFF**F**FFKRR	>1000	---	14.8	188
*S. mitis*-CSP-2-I2MN7FI8F	E**M**RQTH**FF**FFNFFKRR	>1000	---	63.6	13.5
*S. mitis*-CSP-2-N7FI8FF12L	EIRQTH**FF**FFN**L**FKRR	548	434–693	101	67.6
*S. mitis*-CSP-2-E1AN11FF12L	**A**IRQTHNIFF**FL**FKRR	>1000∗	---	---e	---e

Modified residues in the CSP sequence are bolded.

a See the ‘Experimental procedures’ section for experimental details and the Supporting information for details of the reporter strain and plots of agonism or antagonism dose-response curves. All assays were performed in triplicate.

b Data from ref. (2).

c EC_50_ or IC_50_ values were determined by testing peptides over a wide range of concentrations.

d 95% confidence interval.

e EC_50_ not determined due to the analog’s low induction in primary agonism screening assay. See the Supporting information for details.

**Figure 6:**
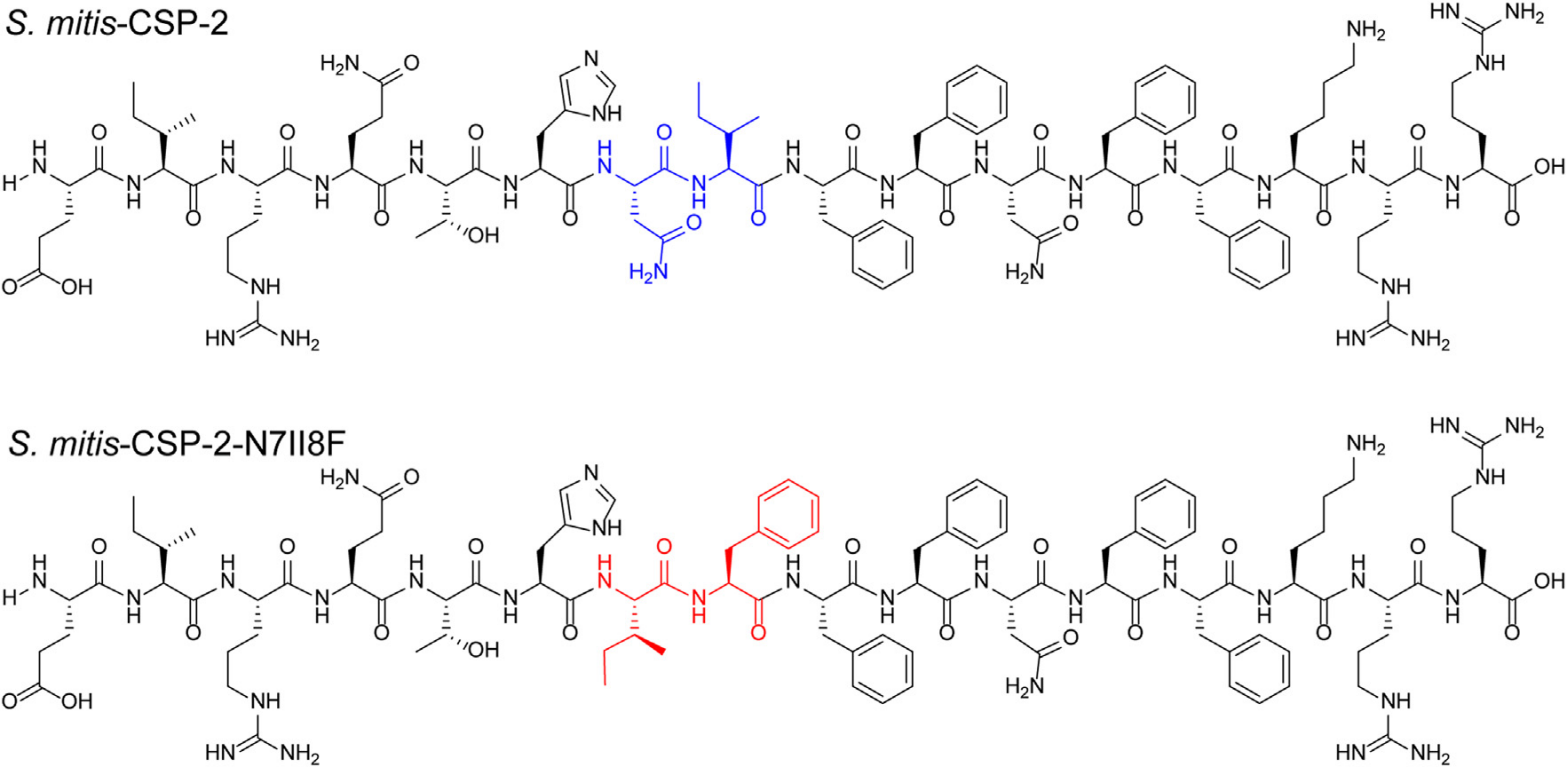
**Structures of *Streptococcus mitis*-CSP-2 and *S. mitis*-CSP-2-N7II8F with the modified residues highlighted**. CSP, competence-stimulating peptide.

In our previous work, we utilized the *S. mitis*-CSP-2 scaffold to develop analogs capable of modulating the two pneumococcal ComD receptors at low nanomolar concentrations (2). We wanted to evaluate whether the optimization of the *S. mitis*-CSP-2 scaffold to the pneumococcus ComD receptors affected the ability of the modified analogs to interact with the *S. mitis* ComD-2 receptor. We therefore set out to test the 20 most potent pneumococcus activators identified in our previous study against the *S. mitis* ComD-2 receptor (Table 5). Our analysis revealed that several potent *S. mitis*-CSP-2–based activators of the pneumococcal QS circuitry are also able to activate the *S. mitis* competence regulon with high potency. In addition to identifying QS modulators that are capable of activating both the pneumococcal and the mitis competence regulon, our analysis also highlighted several important SAR insights regarding ComD receptor binding in both *S. pneumoniae* and *S. mitis*. For example, the singly substituted *S. mitis*-CSP-2–based analog, *S. mitis*-CSP-2-I2M, exhibited high activity against both pneumococcal ComD receptors (EC_50_ values of 87.7 nM and 136 nM against ComD1 and ComD2, respectively, Table 5) (2). However, this mutation resulted in reduced activity of this analog against the *S. mitis* ComD-2 receptor (EC_50_ = 591 nM, Table 5). The same trend was observed for all the other *S. mitis*-CSP-2–based analogs bearing the I2M mutation that were evaluated in this study. These results are in line with previous SAR studies of the pneumococcal CSPs and highlight the importance of the side chain residue in position 2 in both pneumococcal and mitis CSPs for receptor binding and activation. These results also suggest that position 2 has an important role in conferring receptor specificity between the *S. pneumoniae* and *S. mitis* native CSP signals. Importantly, our analysis revealed a highly potent QS activator, *S. mitis*-CSP-2-N7II8F, capable of activating both the pneumococcus ComD receptors and the *S. mitis* ComD-2 receptor at low nanomolar concentrations (EC_50_ values of 87.2 nM and 22.8 nM against the pneumococcal ComD1 and ComD2 receptors, respectively (2), and EC_50_ value of 98.3 against the *S. mitis* ComD-2 receptor, Table 5 and Figure 6). To the best of our knowledge, this is the first example of a CSP-based multispecies QS activator, highlighting the potential of these privileged scaffolds as tools to study the role of QS in the competition between bacterial species in complex bacterial milieu.

Contrary to the *S. mitis*-CSP-2–based pneumococcal QS activators, none of the previously designed *S. mitis*-CSP-2–based pneumococcal QS inhibitors were found to effectively inhibit the *S. mitis* ComD-2 receptor. From 11 *S. mitis*-CSP-2–based potential pneumococcal inhibitors, only *S. mitis*-CSP-2-E1AN11FF12L was found to weakly inhibit the *S. mitis* ComD-2 receptor (Table 5). However, this analog exhibited no inhibitory activity against the *S. pneumoniae* ComD1 and ComD2 receptors (Table 5) (2), highlighting the different structural requirements for receptor inhibition compared to receptor activation in different streptococcal species.

### Defining the regulatory role of the competence regulon QS circuitry in *S. mitis*

Following the confirmation of the native CSP sequence for the *S. mitis* ATCC 49456 strain, we wanted to evaluate the regulatory role of this native CSP signal and the competence regulon QS circuitry. To this end, we first performed RNA-seq analysis of *S. mitis* following treatment with the native CSP or our lead QS inhibitor. Preliminary qPCR data evaluating the expression of the competence genes *comE* and *comX* revealed peak expression of these genes at 10 min post *S. mitis*-CSP-2 treatment (data not shown). Thus, total RNA was isolated for sequencing after a 10-min treatment with either *S. mitis*-CSP-2, *S. mitis*-CSP-2-E1Af10r16, or no treatment (control). Differential gene expression analysis of the *S. mitis*-CSP-2 *versus* control RNA-sequencing data (Table 6) exhibited upregulation of early competence and bacteriocin-associated genes. Conversely, the *S. mitis*-CSP-2-E1Af10r16 inhibitor *versus* control data (Table 7) exhibited downregulation of early competence genes and one gene related to biofilm formation, *gtfA*. Differences in late competence gene expression were not observed, which is in alignment with previous observations by others of a 5-min delay in late competence gene expression relative to the peak in early competence gene expression seen at 10 min post CSP treatment (31, 32, 33, 34).

**Table 6:** *Streptococcus mitis*-CSP-2 *versus* control – Differentially expressed genes

Gene name	Fold change	*p*-value	Product
SM12261_RS08340	6.95	1.3672E-30	Putative competence protein
comA	6.91	6.8685E-27	Peptide cleavage/export ABC transporter ComA
comB	5.91	6.4853E-41	Competence pheromone export protein ComB
SM12261_RS08125	5.12	3.1494E-34	ABC transporter ATP-binding protein
comD	5.04	7.7412E-33	Competence system sensor histidine kinase ComD
comE	4.87	2.5689E-41	Competence system response regulator transcription factor ComE
comC	4.61	3.9256E-32	Competence-stimulating peptide ComC
SM12261_RS08120	4.44	7.6758E-27	ABC transporter permease
SM12261_RS00065	3.89	2.7529E-09	SigX1; sigma-70 family RNA polymerase sigma factor
comW	3.89	4.9906E-18	Sigma(X)-activator ComW
SM12261_RS06180	3.89	0.00081753	Blp family class II bacteriocin
SM12261_RS05165	2.64	2.9241E-17	Bifunctional riboflavin kinase/FAD synthetase
SM12261_RS00270	2.64	0.03477743	Class IIb bacteriocin, lactobin A/cerein 7B family protein
SM12261_RS00285	2.64	0.00080415	Phosphoribosylaminoimidazolesuccinocarboxamide synthase
SM12261_RS03795	2.64	8.2775E-12	Efflux RND transporter periplasmic adaptor subunit
SM12261_RS03800	2.54	6.5851E-14	ABC transporter ATP-binding protein
SM12261_RS06935	2.50	2.7814E-12	Polypeptide deformylase family protein
SM12261_RS06175	2.49	4.7893E-05	Hypothetical protein
SM12261_RS06165	2.44	4.6621E-06	Enterocin A immunity family protein
SM12261_RS00295	2.34	1.0509E-08	Phosphoribosylformylglycinamidine synthase
SM12261_RS03805	2.32	2.3246E-09	macB-like periplasmic core domain protein
SM12261_RS06930	2.28	1.0287E-08	Hypothetical protein

No significantly downregulated genes were observed.

**Table 7:** *S. mitis*-CSP-2-E1Af10r16 inhibitor *versus* control – Differentially expressed genes

Gene name	Fold change	*p*-value	Product
comD	−1.92	2.5434E-06	Competence system sensor histidine kinase ComD
comC	−1.86	0.00016594	Competence-stimulating peptide ComC
SM12261_RS08340	−1.82	0.00278356	Putative competence protein
comE	−1.80	6.2284E-07	Competence system response regulator transcription factor ComE
comA	−1.60	0.00025614	Peptide cleavage/export ABC transporter ComA
SM12261_RS08125	−1.49	0.00285315	ABC transporter ATP-binding protein
SM12261_RS08120	−1.42	0.01888603	ABC transporter permease
comB	−1.33	0.02653476	Competence pheromone export protein ComB
gtfA	−1.25	0.04407347	Sucrose phosphorylase; 1,4-alpha-oligoglucan phosphorylase activity

No significantly upregulated genes were observed.

While we expected to see upregulation in early competence genes post CSP treatment, it was interesting to also observe bacteriocin genes upregulated at such early stages of competence regulation. These findings make sense when we consider the idea that *S. mitis* might first use bacteriocins to attack the membranes of competing bacteria in their biological niche, thereby releasing intracellular DNA, which would then be scavenged and internalized upon production of the late competence proteins.

### Phenotypic evaluation

Our RNA-seq analysis revealed the upregulation of genes involved in competence and bacteriocin production following *S. mitis*-CSP-2 treatment, as well as downregulation of a gene involved in biofilm formation following QS inhibitor treatment. To further validate these observations, we set out to evaluate whether the genomic-level changes translate to changes at the phenotypic level. We therefore focused our analysis on three phenotypes: competence, biofilm formation, and virulence factor production, because genes involved in these phenotypes were found to be modulated in the RNA-seq analysis and because these three phenotypes have also been found to be regulated by the competence regulon QS circuitry in several other streptococcal species (24, 29, 35, 36, 37, 38).

**Figure 7:**
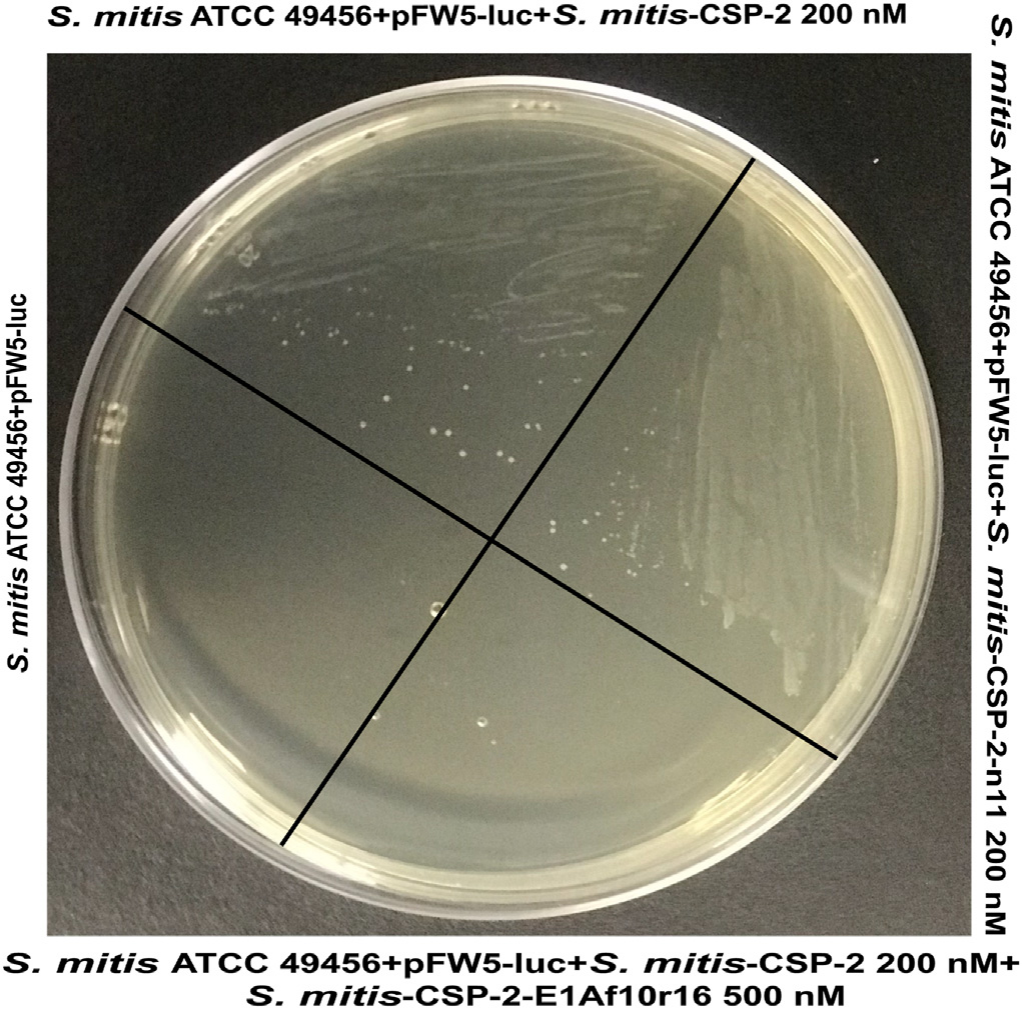
**Transformation assay of *Streptococcus mitis* ATCC 49456 in the presence of *S. mitis*-CSP-2, *S. mitis*-CSP-2-n11, and *S. mitis*-CSP-2 together with *S. mitis*-CSP-2-E1Af10r16.** The ability of ATCC 49456 to internalize a spectinomycin-resistance plasmid (pFW5-luc) was evaluated following treatment with *S. mitis*-CSP-2, *S. mitis*-CSP-2-n11, and *S. mitis*-CSP-2 + *S. mitis*-CSP-2-E1Af10r16. Following treatment with *S. mitis*-CSP-2 or *S. mitis*-CSP-2-n11, ATCC 49456 was able to internalize spectinomycin resistance, as can be seen by the number of transformants (*top* and *right*, respectively). In contrast, ATCC 49456 was unable to internalize spectinomycin resistance following treatment with *S. mitis*-CSP-2 + *S. mitis*-CSP-2-E1Af10r16, as determined by the lack of apparent transformants (*bottom*). The incubation of *S. mitis* ATCC 49456 with the pFW5-luc plasmid without the addition of synthetic CSP was used as a negative control (*left*). The experiment was repeated three times in triplicate for a total of nine experiments. See the Supporting information for full experimental details. CSP, competence-stimulating peptide.

The first phenotype evaluated was competence induction. To this end, an antibiotic resistance transformation assay was conducted (see the Experimental procedures section for details), where a spectinomycin resistance plasmid (pFW5-luc) was introduced to WT *S. mitis* ATCC 49456 and the effectiveness of *S. mitis*-CSP-2, a lead *S. mitis*-CSP-2–based QS activator (*S. mitis*-CSP-2-n11), and a lead *S. mitis*-CSP-2–based inhibitor (*S. mitis*-CSP-2-E1Af10r16) in modulating this phenotype was evaluated. Following a 4-h incubation period with the plasmid and native or synthetic CSPs, the bacteria were spread-plated on Todd-Hewitt + Yeast extract (THY) agar plates containing 5% horse serum and spectinomycin at a final concentration of 200 μg/ml, and competence induction was assessed through colony formation. *S. mitis* ATCC 49456 incubated with the pFW5-luc plasmid alone (no exogenous CSP addition) resulted in no apparent transformants (Fig. 7, left). As expected, incubation with the native *S. mitis*-CSP-2 resulted in many transformants, suggestive of competence induction (Fig. 7, top). Our lead synthetic activator, *S. mitis*-CSP-2-n11, was also able to effectively induce competence, leading to many transformants (Fig. 7, right). Conversely, the addition of native *S. mitis*-CSP-2 together with our lead inhibitor, *S. mitis*-CSP-2-E1Af10r16, did not yield any observed transformants, suggesting that our lead inhibitor was able to block *S. mitis*-CSP-2–mediated competence induction (Fig. 7, bottom).

**Figure 8:**
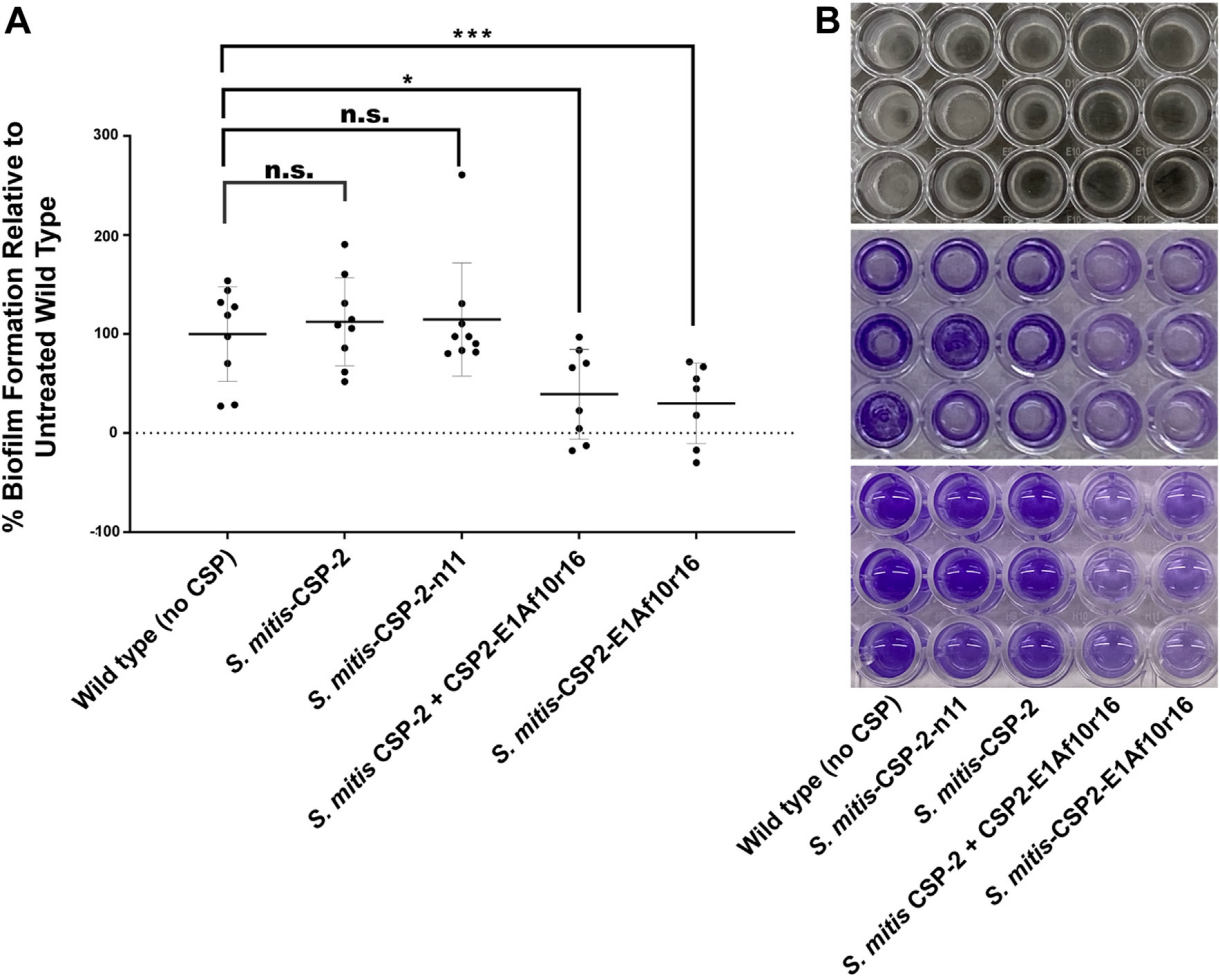
**Biofilm formation of *Streptococcus mitis* ATCC 49456 in the presence of *S. mitis*-CSP-2, *S. mitis*-CSP-2-n11, *S. mitis*-CSP-2 together with *S. mitis*-CSP-2-E1Af10r16 and *S. mitis*-CSP-2-E1Af10r16 alone.***A*, biofilm quantification following treatment with the different CSP analogs, as determined *via* the crystal violet assay. The mean (±S.D.) was as follows: WT, 100% ± 48%; *S. mitis*-CSP-2, 112% ± 44%; *S. mitis*-CSP-2-n11, 115% ± 57%; *S. mitis*-CSP-2 + *S. mitis*-CSP-2-E1Af10r16, 39% ± 45%; and *S. mitis*-CSP-2-E1Af10r16, 30% ± 41%. *B*, representative images of the crystal violet biofilm quantification assay exhibiting the dried biofilms at the bottom of the wells of a 96-well plate (*top*), the dried biofilms following the crystal violet staining (*middle*), and the stained wells after the biofilm and crystal violet were dissolved in 30% acetic acid for quantification (*bottom*). The lead inhibitor, *S. mitis*-CSP-2-E1Af10r16, with or without exogenous addition of the native *S. mitis*-CSP-2 signal, was found to decrease the amount of biofilm formed compared to the untreated control. Statistical significance was determined using a one-way ANOVA with Bonferroni’s correction; n.s., not significant; ∗*p* ≤ 0.1; ∗∗∗*p* ≤ 0.001. The experiment was repeated three times in triplicate for a total of nine experiments. See the Supporting information for full experimental details. CSP, competence-stimulating peptide.

Next, we sought to determine whether biofilm formation, a trait associated with enhanced bacterial pathogenesis, was also under the control of the *S. mitis* competence regulon. To this end, we conducted a crystal violet biofilm quantification assay (see Supporting information for experimental details) in the presence of the native *S. mitis*-CSP-2, our lead activator, *S. mitis*-CSP-2-n11, our lead inhibitor, *S. mitis*-CSP-2-E1Af10r16, or *S. mitis*-CSP-2 together with our lead inhibitor, *S. mitis*-CSP-2-E1Af10r16. Our results revealed that exogenous addition of either the native *S. mitis*-CSP-2 or the lead activator did not significantly alter the amount of biofilm formed compared to untreated *S. mitis* ATCC 49456 (Fig. 8). Contrary, regardless of whether exogenous *S. mitis*-CSP-2 was included or not, inclusion of the lead inhibitor, *S. mitis*-CSP-2-E1Af10r16, at a concentration 5-fold greater than the IC_50_ value (IC_50_ = 87.3 nM, Table 5) resulted in a statistically significant decrease in the amount of biofilm formed than the untreated control (Fig. 8). These results demonstrate the ability of our lead inhibitor to dramatically reduce *S. mitis* biofilms, even when a sufficient concentration of native *S. mitis*-CSP-2 signal is present to induce a QS response.

Lastly, since our RNA-seq analysis revealed the upregulation of genes involved in bacteriocin production, a trait often coupled with competence, we wanted to evaluate whether the produced bacteriocins are cytotoxic. Thus, we set out to assess the cytotoxicity of both the lead *S. mitis-*CSP-2 analogs and *S. mitis*, following treatment with our lead QS modulators. *S. mitis* predominantly colonizes and persists in the human mouth and other mucosal surfaces and was found to have the ability to induce the production of oral mucosal antibodies. These unique biological features categorize *S. mitis* as a potential mucosal vaccine or therapeutic delivery vector (10). Since previously it was reported that many streptococci species produce an array of bacteriocin, many of which also act as virulence factors associated with cytotoxicity (39, 40), we evaluated the potential toxicity of *S. mitis* and the lead *S. mitis-*CSP-2 analogs (native signal, *S. mitis*-CSP-2; lead activator, *S. mitis*-CSP-2-n11; and lead inhibitor, *S. mitis*-CSP-2-E1Af10r16) toward mammalian cells. To this end, we conducted hemolysis assays against defibrinated rabbit red blood cells in both THY media alone and THY media containing the *S. mitis* ATCC 49456 strain. Our results indicate that *S. mitis* as well as all the tested peptides are nontoxic, exhibiting the same degree of hemolysis as the media-only negative control (Fig. S25). These results highlight the potential utility of *S. mitis* as a therapeutic delivery vehicle that does not elicit any apparent cytotoxicity.

## Discussion

It has become evident that bacteria utilize population size-dependent QS pathways to coordinate different phenotypes, including processes involved in pathogenicity. As such, delineating QS circuits, including their regulatory roles, could be used to study bacterial sociality and to develop novel antivirulence therapeutic strategies. In this work, we set out to study the competence regulon QS circuitry in *S. mitis*. Our analysis involved confirming the identity of the native CSP signal utilized by *S. mitis* to assess its population density and initiate the QS response, evaluating the SARs that govern CSP:ComD interactions and lead to ComD and consequently, QS activation and determining the regulatory roles of the competence regulon QS circuitry in initiating potentially pathogenic phenotypes. Our analysis revealed important SAR insights of the CSP signal and facilitated the development of novel CSP-based QS modulators, both enhanced QS activators, and potent competitive inhibitors. Our analysis further demonstrated the involvement of the competence regulon QS circuitry in regulating competence development and biofilm formation, as well as the ability of the CSP-based tools developed in this study to either enhance or attenuate these phenotypes. The CSP-based QS modulators identified in this study can thus be utilized to evaluate the effects temporal QS modulation has on the ability of *S. mitis* to inhabit its natural niche.

It is also now clear that bacteria utilize QS not only for intraspecies communication but also to mediate interspecies communication and interactions. Through the evaluation of the ability of the *S. pneumoniae* and *S. mitis* native CSP signals to modulate the ComD receptor, and thus QS response, of the other species, we found that the native *S. mitis* CSP signal, *S. mitis*-CSP-2, can modulate the QS response in *S. pneumoniae* but that the native CSP signals in *S. pneumoniae* are not effective modulators of QS response in *S. mitis*. It would be interesting to speculate which species would benefit from this potential crosstalk: whether it would be the receiver species, *S. pneumoniae*, who eavesdrops into *S. mitis* population size and at high *S. mitis* population density releases virulence determinants to eliminate the competition and acquire potentially useful genetic material; or whether it would be the producer species, *S. mitis*, who intentionally triggers the competence regulon QS circuitry in *S. pneumoniae* to take advantage of the highly pathogenic nature of pneumococcus and the associated production and release of a myriad of virulence determinant by *S. pneumoniae* to effectively acquire genetic material from the environment. To test these hypotheses in complex bacterial communities or in *in vivo* settings, temporal regulation of QS in either species, or both species, would be needed. As such, the *S. mitis*-CSP-2–based QS modulator capable of activating both the pneumococcus ComD receptors and the *S. mitis* ComD-2 receptor with high potencies (*S. mitis*-CSP-2-N7II8F; EC_50_ values in the low nanomolar range against all three receptors) that was identified in this study, to the best of our knowledge, the first example of a CSP-based multispecies QS activator, would be of great value to study this potential crosstalk between a commensal (*S. mitis*) and pathogenic (*S. pneumoniae*) species. Such studies are ongoing in our laboratory and will be reported in due course.

From a translational standpoint, we envision that the chemical tools developed in this study could be utilized in the future to shift the balance between *S. pneumoniae* and *S. mitis*, allowing *S. mitis* (a commensal species) to outcompete and replace pathogenic *S. pneumoniae*, thereby reducing *S. pneumoniae*–related infections and improving human health. The specificity and lack of toxicity associated with native and synthetic CSPs, along with their ability to modulate bacterial behavior and fitness without directly killing them, make this class of antivirulence compounds an attractive alternative to traditional antibiotics.

## Experimental procedures

### General

The peptide analogs were synthesized using standard Fmoc solid-phase peptide synthesis protocols (41), followed by purification using RP-HPLC to >95% purity. Masses of purified peptides were confirmed by mass spectrometry. See Tables S2–S5 for peptide masses and purities. See the Supporting information for full experimental details.

### Isolation of crude peptides from bacterial supernatants

The native *S. mitis*-CSP-2 was isolated from cell-free bacterial supernatants using previously described methods with minor modifications (29). For full experimental details, see the Supporting information.

### Development of *S. mitis* luciferase-based reporter system

The *S. mitis* luciferase-based reporter strain was constructed using previously described methods with some modifications (3). See the Supporting information for full experimental details.

### Luminescence reporter assay

#### Activation assays

The ability of synthetic *S. mitis*-CSP-2 analogs to activate the expression of *S. mitis comX* was determined using the constructed *S. mitis* luciferase reporter strain. First, bacteria from the *S. mitis* luciferase reporter strain freezer stock were streaked onto a Todd-Hewitt broth supplemented with 0.5% yeast extract (THY) agar plate containing 5% horse serum and spectinomycin at a final concentration of 200 μg/ml. The plate was incubated overnight in a CO_2_ incubator (37 °C with 5% CO_2_). Fresh colonies were transferred to Tryptic soy broth supplemented with spectinomycin at a final concentration of 200 μg/ml, and the culture was incubated at 37 °C in a 5% CO_2_-supplemented atmosphere and grown overnight (16 h) until the culture reached an absorbance at 600 nm (optical density at 600 nm [A_600_]) of 0.45. Overnight cultures of the *S. mitis* reporter strain at A_600_ 0.45 were diluted 1:10 in Tryptic soy broth and the liquid cultures were incubated in a CO_2_ incubator for 30 to 45 min, until the bacteria reached early exponential stage (A_600_ ∼ 0.2) as determined by using a plate reader. During incubation, clear-bottom white 96-well microtiter plates were prepared for the activation assays. An initial activation screening was performed at a high peptide concentration (10,000 nM) for all the *S. mitis*-CSP-2 analogs (Figs. S1–S7). For each experimental sample, a total of 2 μl of a 1 mM CSP stock solution in dimethyl sulfoxide (DMSO) was added in triplicate to the clear-bottom white 96-well microtiter plate. A total of 2 μl of DMSO was added in triplicate and served as the negative control, and 2 μl of a 1 mM stock of native *S. mitis*-CSP-2 was added in triplicate as a positive control. In the dark, 2 μl of a 15 mg/ml D-luciferin stock in distilled water was added to each well. Following the addition of synthetic CSPs and D-luciferin, 196 μl of the diluted bacterial culture was added to each well, and the plate was incubated for 30 min at 37 °C. Following incubation, the A_600_ and luminescence of each experimental well were measured. Results were reported as percent activation, which is the ratio between the luminescence (presented as relative luminescence units, RLU/A_600_) of the analog and that of the positive control. Analogs that exhibited activity >75% compared to the positive control were further evaluated using a dose-dependent assay in which peptide stock solutions were diluted with DMSO in serial dilutions (1:2,1:3, or 1:5) and assayed as described above. EC_50_ values, the concentration of the peptide to achieve a half maximal response, for each activator were calculated using GraphPad Prism (https://www.graphpad.com/). Experiments were performed in triplicate on three separate days.

#### Inhibition assays

Analogs that exhibited less than 50% activation in the initial screening were further evaluated for potential competitive inhibition of the competence regulon (Figs. S8–S13). The ability of synthetic *S. mitis*-CSP-2 analogs to competitively inhibit *comX* expression by outcompeting the native *S. mitis*-CSP-2 for the receptor-binding site was evaluated using the same assay conditions as those for the activation assays, except that for the inhibition screening, the native *S. mitis*-CSP-2 was introduced to each well at a set concentration (1 μM) that was chosen to afford complete activation of the competence regulon, as determined from dose-dependent curves created for the native *S. mitis*-CSP-2. As a positive control, 2 μl of *S. mitis*-CSP-2 and 2 μl of DMSO were added to the same well in triplicate. As a negative control, 4 μl of DMSO was added in triplicate. Then, 2 μl of 15 mg/ml D-luciferin in distilled water was added to each experimental or control well, after which 194 μl of bacteria was added, and the plate was incubated for 30 min at 37 °C. Following incubation, the A_600_ and luminescence of each experimental well were measured. Results were reported as percent activation, which is the ratio between the luminescence (presented as relative luminescence units, RLU/A_600_) of the analog and that of the positive control. Analogs that exhibited significant competitive inhibition in the initial screening were further evaluated using a dose-dependent assay where peptide stock solutions were diluted with DMSO in serial dilutions (either 1:2, 1:3, or 1:5) and assayed as described above. GraphPad Prism was used to calculate the IC_50_ values, which are the concentration of inhibitor where the response (or binding) is reduced by half. Experiments were performed in triplicate on three separate days.

### Transformation assay

A single colony of *S. mitis* ATCC 49456 was grown overnight in 5 ml of THY media (pH 7.3) at 37 °C with 5% CO_2_. Following incubation, *S. mitis* ATCC 49456 was diluted 1:100 into fresh THY media containing 5% horse serum and the culture was incubated at 37 °C in a 5% CO_2_-supplemented atmosphere for 4 to 5 h (until the culture reached an A_600_ of 0.30). Then, 100 μl of this diluted culture was inoculated to 900 μl of fresh THY media containing 5% horse serum. Following inoculation, synthetic *S. mitis*-CSP-2 (at a final concentration of 200 nM), the lead activator, *S. mitis*-CSP-2-n11 (at a final concentration of 200 nM), or *S. mitis*-CSP-2 (at a final concentration of 200 nM) together with the lead inhibitor, *S. mitis*-CSP-2-E1Af10r16 (at a final concentration of 500 nM), were added to the culture. After that, pFW5-luc (Spec^R^) plasmid at a final concentration of 1 μg/ml was added to the culture. Parallel assays without the *S. mitis*-CSP-2 or without both the *S. mitis*-CSP-2 and plasmid were used as controls to assess the indigenous competence or antibiotic resistance of *S. mitis* under the tested conditions. After 3 to 4 h of incubation at 37 °C, 200 μl of the culture was plated on THY agar containing 200 μg/ml spectinomycin and incubated at 37 °C with 5% CO_2_ for 24 to 48 h to identify positive transformants. Experiments were performed in triplicate on three separate days.

### Biofilm-formation assay

Biofilm formation was assessed using previously described protocols with minor modifications (29). See the Supporting information for full experimental details.

### Hemolysis assay

Hemolysis was assessed using previously described protocols with minor modifications (29). See the Supporting information for full experimental details.

### CD spectroscopy

CD spectra were recorded using an Aviv Biomedical CD spectrometer (model 202–01). For experimental details, see the Supporting information.

### RNA sequencing

*S. mitis* strain ATCC 49456 was streaked on THY agar plates and grown in a static incubator set to 37 °C + 5% CO_2_ for 22 to 24 h. From these plates, single colonies were used to inoculate 3 ml of THY media for overnight cultures (14–16 h of growth). The overnight cultures were then diluted 1:100 and grown to an A_600nm_ value of 0.25 to 0.3 (early log phase). Four milliliters of culture was then treated for 10 min with final concentrations of either 592 nM (4× EC_50_) *S. mitis*-CSP-2, 350 nM (4x IC_50_) *S. mitis*-CSP-2-E1Af10r16, or no treatment (control), in the 37 °C + 5% CO_2_ static incubator. Each 4 ml culture was then added to 8 ml of RNAprotect Bacteria Reagent (Qiagen) in 15 ml microcentrifuge tubes, mixed by inversion, then incubated at room temperature for 5 min. The bacterial cells were then pelleted by centrifugation at 4550 RPM and 4 °C for 10 min on an Avanti J-15R Centrifuge (Beckman Coulter). The supernatants were discarded, then the 15 ml tubes were gently tapped upside down on a paper towel to remove remaining supernatant. The tubes were recapped, then placed in liquid nitrogen for 5 min to snap-freeze the cell pellets, which were then stored in a −80 °C freezer for later RNA isolation.

RNA isolation was conducted using an RNeasy Mini Kit (Qiagen), with minor protocol modifications. First, the cell pellets were thawed on ice, then resuspended by pipetting in 100 μl of cold TE buffer. The resuspended cells were then added to 100 μl of acid-washed 0.1 mm glass beads in a 2 ml RNase-free tube and run at 4.0 m/s for 20 s on a FastPrep-24 Classic bench-top bead beating lysis system. The tubes were then centrifuged at 14,000*g* for 30 s to pellet the glass beads and remove bubbles. Next, 650 μl of buffer RLT (Qiagen) containing 1% 2-mercaptoethanol was added to each tube. The tubes were subjected to one more bead beating run at 4.0 m/s for 20 s, then centrifuged at 14,000*g* for 30 s to pellet the glass beads and remove bubbles. Between each step in these bead beating/centrifugation cycles, the tubes were kept on ice to minimize RNA degradation. From this point forward, the manufacturer protocol was followed for the Qiagen RNeasy Mini Kit.

The isolated crude RNA was then treated with DNase I, using the Turbo DNA-free kit (Invitrogen), in three 40-min treatment cycles totaling 2 h, followed by DNase inactivation following the manufacturer protocol. The integrity of the purified RNA was then verified using a Tapestation instrument (Agilent). Only samples that did not show RNA degradation and had a RIN value of >7.0 were used for RNA-seq. The subsequent Illumina library preparation and sequencing was conducted by the Nevada Genomics Center at the University of Nevada. The sequencing library preparation kit used was the Stranded Total RNA Prep with Ribo-Zero Plus kit (Illumina). Paired-end sequencing was conducted at 200 cycles (2 × 100 bp) on a NextSeq 2000 (Illumina), yielding >16M reads per sample. The RNA-seq analysis and differential gene expression analysis was conducted using CLC Genomics Workbench 21 (Qiagen). The RNA-seq data were collected from three biological replicates. Raw sequencing reads data can be obtained from NCBI (BioProject ID: PRJNA1022158).

## Data availability

All data are presented in the article. Raw sequencing reads data can be obtained from NCBI (BioProject ID: PRJNA1022158).

## Supporting information

This article contains supporting information (2).

## Conflict of interest

The authors declare that they have no conflicts of interest with the contents of this article.
