# Supplementary material for: Developing multispecies quorum-sensing modulators based on the Streptococcus mitis competence-stimulating peptide
Source: J Biol Chem. 2023 Nov 10;299(12):105448. doi: 10.1016/j.jbc.2023.105448 (PMC10714334; doi:10.1016/j.jbc.2023.105448)
Supplement: Supporting Information Tables S1–S5 and Figures S1–S25 [file mmc1.pdf]

Developing Multi-Species Quorum Sensing Modulators Based on the *Streptococcus mitis* Competence-Stimulating Peptide

Tahmina A. Milly, Clay P. Renshaw, and Yftah Tal-Gan*

Department of Chemistry, University of Nevada, Reno, 1664 North Virginia Street, Reno, Nevada, 89557, United States

* To whom correspondence should be addressed. ytalgan@unr.edu

Supporting Information

Full experimental methods……………………………………………………………S-2

HPLC traces for *S. mitis*-CSP-2 analogs…..……………………………………………… S-9

MS and HPLC data for *S. mitis*-CSP-2 analogs…………………………………………S-29

Primary reporter assay data ………………………………………………………..S-31

Agonism and antagonism dose response curves………………………………………...S-38

Circular dichroism (CD) spectra…………………………………………......................S-49

MS/MS analysis of extracted and synthetic *S. mitis*-CSP-2………………………………..S-57

Reference……………………………………………………………………………...…S-59

Full Experimental Methods

Chemical Reagents and Instrumentation:

All chemical reagents and solvents were purchased from Chem-Impex or Sigma-Aldrich and used without further purification. Water (18 MΩ) was purified using a Thermo Scientific Smart2- Pure Pro UV/UF 16 LPH water purification system. Solid-Phase resin was purchased from Chem-Impex. Reversed-phase high-performance liquid chromatography (RP-HPLC) was performed using a Shimadzu system equipped with a CBM-20A communications bus module, two LC-20AT pumps, an SIL-20A auto sampler, an SPD-20A UV/VIS detector, a CTO-20A column oven, and an FRC-10A fraction collector. All RP-HPLC solvents (18 MΩ water and HPLC-grade acetonitrile (ACN)) contained 0.1% trifluoroacetic acid (TFA). Matrix-assisted laser desorption ionization time-of-flight mass spectrometry (MALDI-TOF MS) data were obtained on a Bruker Microflex spectrometer equipped with a 60 Hz nitrogen laser and a reflectron. In positive ion mode, the acceleration voltage on Ion Source 1 was 19.01 kV. Exact mass (EM) data were obtained on an Agilent Technologies 6230 TOF LC/MS spectrometer. The samples were sprayed with a capillary voltage of 3500 V and the electrospray ionization (ESI) source parameters were as follows: gas temperature of 325 °C at a drying gas flow rate of 8 L/min at a pressure of 35 psi.

Solid Phase Peptide Synthesis:

All the *S. mitis*-CSP-2 analogs were synthesized using standard Fluorenyl methoxycarbonyl (Fmoc)-based solid-phase peptide synthesis (SPPS) procedures on preloaded Fmoc-L-Arg (Pbf) Wang resin (0.305 mmol g^{-1}) by using a Liberty 1 automated peptide synthesizer (CEM Corporation). The resin (0.1 g) was first swelled by suspension in *N*, *N*-dimethylformamide (DMF) for 15 min at room temperature and then drained. Fmoc removal was accomplished with treatment of the resin by 5 mL of 20% piperidine in DMF (90 s, 90 °C) followed by another 5 mL of 20% piperidine in DMF (90 s, 90 °C). The resin was then washed with DMF (3 X 5 mL) after each deprotection cycle. To couple each amino acid, Fmoc-protected amino acids (5 equiv. relative to the overall loading of the resin) were dissolved in DMF (5 mL) and mixed with 2-(1H-benzotriazol-1-yl)-1,1,3,3- tetramethyluronium hexafluorophosphate (HBTU; 5 equiv.) and diisopropylethylamine (DIPEA; 5 equiv.). All amino acids were coupled for 5 min (30 W, 75 °C). Arginine residues present within the sequence were double coupled. Following completion of the coupling reaction, the resin was drained and washed with DMF (2 X 5 mL). This process was repeated until the desired peptide sequence was obtained.

Peptide cleavage from solid support:

Following coupling of the final residue, the resin was washed three times with 2 mL DCM with manual shaking for 1 min. The resin was washed with 2 mL diethyl ether and dried under nitrogen stream for 3 min and then transferred into a 15 mL falcon tube. A 3 mL solution of 2.5% 18 MΩ water and 2.5% triisopropylsilane (TIPS) in 95% trifluoroacetic acid (TFA) for every 0.1 g of resin was added and the tube was shaken for 3 h at 200 rpm. Following completion of the cleavage reaction, the resin was filtered using a cotton plug in a polypropylene syringe and washed with a

small amount of TFA. The filtrate was collected in a 50 mL polypropylene centrifuge tube, and a cooled solution of diethyl ether:hexane (1:1, 45 mL, -20 °C) was added to the tube. To precipitate the crude peptide, the tube was kept in a freezer for 10 min at -20 °C. The pellet of the crude peptide was obtained by centrifugation of the 50 mL tube in a Beckman Coulter Allegra 6 centrifuge equipped with a GH3.8 rotor at 3000 RPM for 5 min. Following centrifugation, the 1:1 ether:hexane solution was removed, and the solid peptide product was re-dissolved in 1:1 ACN:water, frozen in a dry ice-acetone bath, and then lyophilized for a minimum of 24 h.

Peptide Purification by HPLC:

Crude peptides were purified using RP-HPLC. The crude peptide was dissolved in ACN: H_2O (1:4; volume of ACN in water depends on the solubility of the peptide) and purified in 4 mL portions on a Phenomenex Kinetex 5 μm C18 semi-preparative column (10 mm x 250 mm, 110 Å) with a flow rate of 5 mL min^{-1}; mobile phase A = 18 MΩ water + 0.1% TFA and mobile phase B = ACN + 0.1% TFA. Initially, crude peptides were purified with a linear gradient from 5% to 40% ACN in 46 min. After identifying the relative retention time of these peptides, a second preparatory run was performed using a 10% ACN gradient centered on the ACN concentration where peptide elution was observed, for example a 15% to 25% ACN gradient for a peptide that eluted at 20% ACN during the first run. These conditions were typically sufficient to purify the peptides to ≥ 95%. Fraction purity was determined through analysis on a Phenomenex Kinetex 5 μm analytic C18 column (4.6 mm x 250 mm, 110 Å). Purities were determined by integration of peaks with UV detection at 220 nm. The gradient used for analytical analysis was from 5% to 95% ACN over 24 min. Following purification, peptides were frozen with a dry ice-acetone bath, and then lyophilized for a minimum of 24 h. Before the final masses and yields of purified peptides were determined, peptides were dissolved in 25% acetic acid in up to 1:1 ACN:water to allow removal of any residual TFA. The solution was then frozen and lyophilized for at least 24 h before peptide DMSO stocks were made for bioassays.

Peptide Verification with Mass Spectrometry:

Following purification of crude peptide, peaks were verified to contain the desired peptide mass by MALDI-TOF MS. Samples were prepared using 1 μL α-Cyano-4-hydroxycinammic acid (10 μg) in 1:1 water:ACN as a matrix and 1 μL of the desired peptide fraction. Final verification of the peptide mass was conducted by obtaining their exact masses with a high-resolution ESI-TOF MS (**Tables S-2 to S-5**). The instrument was calibrated before each run and an internal reference mass standard was used.

Isolation of Crude Peptides from Bacterial Supernatants:

Overnight *S. mitis* ATCC culture (200 mL) was centrifuged at 4,500 rpm for 10 min. The supernatants were fractionated and filtered through a sterile 0.22-μm polyether sulfone (PES) filter into sterile 50 ml centrifuge tubes. Ammonium sulfate was added to the supernatants to afford a 40% (wt/vol) concentration and the solutions were mixed by inversion until the ammonium sulfate was completely dissolved. The solution was stored at 4 °C for 1 hour, followed by centrifugation at 4,500 rpm for 15 min. The supernatants were then carefully decanted from the centrifuged tubes and the remaining pellet was dissolved in 10 mL of ddH_2O:ACN (1:1), after which all of the

fractions were combined into a single centrifuge tube and freeze-dried. The lyophilized material was then purified by RP-HPLC. Following purification, the mass of the extracted peptide was verified using a high-resolution ESI-TOF MS and through MS/MS analysis.

MS/MS analysis:

In 1% Formic Acid in 1:1 H_2O:Methanol (mass spectrometry grade), either the extracted or synthetic *S. mitis*-CSP-2 peptide was dissolved to a final concentration of ~0.5-1.0 µM. To remove any fine particles, the solutions were filter sterilized using 0.45 µm syringe filters. For MS and MS/MS analysis, a ThermoFisher™ Orbitrap Fusion™ Tribrid™ Mass Spectrometer was used. From the MS data, the two highest intensity peaks (MH^{+3} and MH^{+4} for the extracted peptide; MH^{+4} & MH^{+5} for the synthetic peptide) were isolated using quadrupole isolation and fragmented using higher energy collisionally activated dissociation (HCD). The Orbitrap™ detector was used at a resolution of 60,000 for both peptides. To determine the HCD energy for optimal peptide fragmentation, an HCD energy scan from 0-50% was performed (data not shown) using 5% intervals. For the final MS/MS fragmentation, the HCD energy was set to 43% (MH^{+3}) and 42% (MH^{+4}) for the extracted peptide and 41% (MH^{+4}) and 24% (MH^{+5}) for the synthetic peptide. All MS and MS/MS data were copied to Microsoft Excel and analyzed using Origin Pro.

Development of *S. mitis* ATCC 49456 reporter strain:

Bacterial gDNA extraction: An isolated fresh single colony of *S. mitis* ATCC 49456 was picked into a sterilized cultural tube containing 5 mL of THY media (pH 7.3) and the culture was incubated in a CO_2 incubator overnight (16 hours). Following incubation, 1.5 mL of pure culture broth was added to a sterile 1.5 mL microcentrifuge tube. Cells were pelleted by centrifugation at 10,000 rpm for 1 min. Following centrifugation, the supernatant was discarded, and 0.5 mL sterile 1x phosphate buffered saline (PBS) (137 mM NaCl, 2.7 mM KCl, 10 mM Na_2HPO_4, 1.8 mM KH_2PO_4; pH adjusted to 7.2 - 7.4) was added to the tube containing the bacterial cell pellet. The pellet was then resuspended using 5-sec pulses with a vortex mixer set at 4,000 rpm. The cells were pelleted again under the same centrifugation conditions as above. The supernatant was discarded, and the process was repeated twice more for a total of three times. After the PBS washes, 0.5 mL of sterile distilled water (dH_2O) was added to the bacterial pellet, and it was resuspended by pulse vortex mixing at 4,000 rpm. Using the same conditions, the cells were pelleted. The supernatant was discarded, and 200 µL of sterile distilled water (dH_2O) was added to the tube. The pellet was again resuspended by vortex mixing at 4,000 rpm, incubated in a standard heat block set at 95 °C for 5 min, and immediately placed in a −80 °C freezer for 10 min. The tubes were then kept at room temperature to thaw completely and gently mixed with a vortex mixer set at 4,000 rpm using 5-sec pulses, followed by centrifugation at 5,000 rpm for 60 sec. The supernatant was then transferred to a new, sterile, 0.6 mL microcentrifuge tube and stored at −20 °C.

***S. mitis* ATCC 49456 *comX* promoter amplification:** The promoter region (911 bp) of the *S. mitis* ATCC 49456 *comX* gene was amplified from extracted bacteria gDNA using the primer pair SmATCCComXfwd and SmATCCComXrev (**Table S-1**) on an Eppendorf Mastercycler Gradient 5331 PCR machine. For *comX* PCR amplification, total reaction volume of 25 µL was prepared containing 12.5 µL Hot Start Taq Master Mix (VWR), 2.5 µL template DNA, 2.5 µL of 1 µM

primer SmATCCComXfwd, 2.5 μL of 1 μM primer SmATCCComXrev, and 5 μL nuclease free water. PCR amplification consisted of the following steps: 3 min initial denaturation at 95 °C to activate the polymerase, followed by 35 cycles of 30 sec denaturation at 95 °C, 1 min of annealing at 59 °C, and 1 min of extension at 72 °C, followed by a final extension at 72 °C for 7 min. Following amplification, PCR products were run on a 1% agarose gel to verify the presence of the *comX* PCR amplicon containing ends homologous to the restriction sites for *BamHI* and *NheI* (911 bp). Once verified, PCR products were purified using a E.Z.N.A Cycle Pure Kit (Omega Bio-Tek).

Table S-1. Primers used for the design of the *S. mitis* ATCC 49456 luciferase reporter

Primer	Sequence
SmATCCComXfwd	aaagctagcAGCTGCTTTAGTCGCTGCTC[a]
SmATCCComXrev	aaaggatccCAATCCCCTGGACTTCTTCA[b]

[a]*BamHI* restriction site underlined. [b]*NheI* restriction site underlined

Restriction digestion: The plasmid pFW5-luc (Spec^R) was extracted and purified from *Escherichia coli* using a GenElute Plasmid Miniprep Kit (Sigma). Both pFW5-luc and the PCR amplified *comX* promoter amplicon were then restriction digested using 1 μg DNA, 5 μL 10X CutSmart Buffer (NEB), 1 μL *NheI-HF* (NEB), 1 μL *BamHI-HF* (NEB) and nuclease free water to bring the total reaction volume up to 50 μL. The restriction digestion was carried out for 15 min at 37 °C, followed by heat inactivation of the restriction enzymes at 80 °C for 20 min. Following restriction digestion, digested products were run on a 1% agarose gel to verify the presence of bands with a different size than undigested plasmid or PCR amplified *comX* promoter, respectively. After the verification of the correct amplicon, digested product was purified using a E.Z.N.A Cycle Pure Kit (Omega Bio-Tek).

Ligation and cloning: Ligation of purified restriction digested pFW5-luc (Spec^R) and PCR amplified *comX* promoter was carried out in a microcentrifuge tube placed on ice. Ligation was carried out at an insert to vector ratio of 3:1. Ligation mixture was prepared with the addition of 2 μL of 10X T4 DNA ligase reaction buffer (NEB), 1 μL of T4 DNA ligase (NEB), and nuclease free water to bring the total reaction volume up to 20 μL. Then the ligation mixture was gently mixed by pipetting up and down, and the ligation reaction of the insert and vector was performed at 16 °C overnight. After the completion of ligation reaction, heat inactivation of the ligated construct was performed at 65 °C for 10 min. Following that, the ligated construct was transformed into competent *E. coli*. Competent *E. coli* were removed from a -80 °C freezer, thawed at room temperature, and immediately placed on ice for 10 min. Ligated construct to be transformed was added at a concentration of 10 ng per 50 μL of competent cells, with the total volume of added DNA not exceeding 5% that of the competent cells. Tubes containing the competent *E. coli* and construct DNA were gently inverted several times to mix contents to homogeneity, then the tubes were placed on ice for 30 min. Next, the tubes were placed in a 42 °C heat bath for 90 sec, and immediately transferred to an ice bath for 2 min. Following that, 400 μL of SOC media (2% tryptone, 0.5% yeast extract, 10 mM NaCl, 2.5 mM KCl, 10 mM $MgCl_2$, 10 mM $MgSO_4$, and 20 mM glucose) was added to the tube, which was then transferred to a 37 °C shaking incubator set at 200 rpm for 45 min. The entire content of the tube was then transferred to

a LB agar plate containing 100 μg/mL spectinomycin. The plate was then incubated overnight at 37 °C and was checked for the presence of positive transformants the following day.

Construct transformation: A positive *E. coli* transformant was inoculated into 5 mL of LB media containing 100 μg/mL spectinomycin, which was then grown overnight at 37 °C with shaking at 200 rpm. Purified plasmid construct was extracted from the *E. coli* overnight culture using a GenElute Plasmid Miniprep Kit (Sigma). A small amount of the extracted plasmid was subjected to restriction digestion by *BamHI-HF* and *NheI-HF* using the above conditions and the presence of the correct vector and insert was verified. Successful constructs were identified as containing two bands corresponding to the approximate size of the vector and insert when run on a 1% agarose gel. Following that, a single colony of *S. mitis* ATCC 49456 was inoculated into 5 mL THY media and grown overnight (16 h) at 37 °C with 5% CO_2. Then the overnight culture was diluted 1:100 in fresh THY media and was incubated at 37 °C in a 5% CO_2-supplemented atmosphere for 4-5 h until the culture reached an absorbance at 600 nm (optical density at 600 nm [OD_{600}]) of 0.30. Following incubation, 100 µL of culture was transferred to 900 µL of THY media and then synthetic *S. mitis*-CSP-2 at a final concentration of 200 nM and plasmid construct at a final concentration of 1 µg/mL were added to the diluted culture, and the bacteria were subjected to another 3 to 4 h growth at 37 °C. After 3 to 4 h of incubation at 37 °C, 200 μL of the culture was plated on THY agar containing 200 μg/mL spectinomycin and incubated at 37 °C with 5% CO_2 for 24 to 48 h to identify positive transformants. A single colony was then picked and grown for 16 h in 5 mL TSB media containing 200 μg/mL spectinomycin at 37 °C with 5% CO_2. Following overnight growth, the *S. mitis* culture was diluted 1:10 in fresh TSB media, and the presence of the reporter plasmid was validated by both sequencing of the *comX* promoter region and the observation of luminescence following treatment with exogenous *S. mitis*-CSP-2 (10,000 nM) and D-luciferin.

Biofilm Formation Assay:

A single colony of *S. mitis* ATCC 49456 was grown for 16 hours in 5 mL THY media (pH 7.3) at 37 °C with 5% CO_2. Following incubation, *S. mitis* ATCC 49456 was diluted 1:10 in fresh THY media containing 1% D-glucose, and 196 μL of culture was added in triplicate to a 96-well microtiter plate. Two μL of *S. mitis*-CSP-2 (at a final concentration of 200 nM), the lead activator, *S. mitis*-CSP-2-n11 (at a final concentration of 200 nM), or *S. mitis*-CSP-2 (at a final concentration of 200 nM) together with the lead inhibitor, *S. mitis*-CSP-2-E1Af10r16 (at a final concentration of 500 nM) in 2 μL of DMSO were added to the culture. For background subtraction, a set of wells containing only 196 μL of THY media containing 1% D-glucose and 4 μL of DMSO (no bacteria) was included. The plate was then incubated for 24 hours at 37 °C with 5% CO_2. Following 24 h incubation, the optical density at 600 nm (OD_{600}) was recorded. The contents of all wells were carefully decanted by shaking the plate gently over a glass basin. Experimental wells were then gently washed with 100 μL 1x PBS. The 96 well microtiter plate was then incubated at 55 °C for 2.5 h to heat fix bacterial biofilms to the bottom of the well. Two hundred μL of a 0.05% crystal violet solution was then added to each well, and the solution was kept at room temperature for 5 min. The wells were then carefully decanted and washed with 200 μL distilled water, and a total of 200 μL of a 30% (v/v) acetic acid in distilled water was added to the wells. The plate was placed

in shaker at 150 rpm for 15 min at 37 °C, and experimental wells were then further diluted 1:5 in distilled water. The absorbance at 595 nm (OD_{595}) was then measured for each well. Each OD_{595} value was divided by its corresponding OD_{600} values. Experiments were performed in triplicate on three separate days. Data are presented as the percent biofilm formation relative to wild type untreated with exogenous *S. mitis*-CSP-2. Results are expressed as the mean +/- the standard deviation of three independent experiments.

Hemolysis Assay:

A single colony of *S. mitis* ATCC 49456 was grown for 16 h at 37 °C with 5% CO_2 in 5 mL THY media (pH 7.3). Overnight cultures were then diluted 1:10 in fresh THY media. Samples were prepared in clear bottom 96-well microtiter plates. Experimental samples were prepared by adding 196 μL of the 1:10 diluted culture and 2 μL of a 1 mM *S. mitis*-CSP-2 and the lead *S. mitis*-CSP-2 analogs (lead activator, *S. mitis*-CSP-2-n11, and lead inhibitor, *S. mitis*-CSP-2-E1Af10r16), or 196 μL of fresh THY media and 2 μL of a 1 mM *S. mitis*-CSP-2/ *S. mitis*-CSP-2 analog stock. Two μL of DMSO was added to each well. A positive control was prepared by adding 2 μL of a 1% Triton X solution and 2 μL of DMSO in 196 μL THY media, and negative controls were prepared by adding 4 μL DMSO in either 196 μL 1:10 diluted culture or 196 μL THY media. The 96-well microtiter plate was then incubated for 2 h at 37 °C with 5% CO_2, after which the optical density at 600 nm (OD_{600}) was recorded and hemolysis was assessed. A 1 mL portion of defibrinated rabbit red blood cells (VWR International) was aliquoted into a sterile 1.5 mL microcentrifuge tube, and centrifuged at 2,000 rpm for 2 min. Following centrifugation, the top layer was aliquoted off, and red blood cells were gently washed with sterile1 mL 1X PBS. This process was repeated for a total of three times, until following centrifugation the top layer was mostly clear. Washed red blood cells were resuspended in sterile 1 mL 1X PBS solution, and a 15 μL aliquot was added to each well of the 96-well microtiter plate. The plate was then incubated for 30 min at 37 °C. Following incubation, the plate was centrifuged for 4 min at 4 °C at 1600 rpm. Experimental wells were then diluted 1:5 in distilled water to prevent saturation of the detector by the positive control. The absorbance at 420 nm (OD_{420}) was then measured for each well. Experiments were performed in triplicate on three separate days. Data are presented as the percent hemolysis relative to the .01% Triton X positive control. Results are expressed as the mean +/- the standard deviation of three independent experiments.

Circular Dichroism (CD) Spectroscopy:

CD spectra were recorded using an Aviv Biomedical CD spectrometer (model 202-01). All the measurements were performed with a peptide concentration of 200 μM in 1X PBS buffer (137 mM NaCl, 2.7 mM KCl, 10 mM Na_2HPO_4, 1.8 mM KH_2PO_4; pH was adjusted to 7.4) with 0% or 20% TFE. Measurements were performed at 25 °C using a quartz cuvette (Starna Cells) with a pathlength of 0.1 cm. Samples were scanned once at 3 nm min^{-1} with a bandwidth of 1 nm and a response time of 20 sec over a wavelength range (195 to 260 nm). Single scans were acquired and corrected for their respective solvent concentrations and then converted to mean residue ellipticity (MRE) values using the following equation:

$$\mathrm{MRE} = \left(\frac{\theta}{10 \times c \times l}\right)/n$$

θ is the observed ellipticity in millidegrees, c is the molar peptide concentration, l is the pathlength in centimeters, and n is the number of residues in the peptide sequence. Percent helicity (f_H) was calculated for all peptide analogs using the following equation:

$$f_H = \frac{[\theta_{222}]}{[\theta_{\infty}]_{222}\left(1 - \frac{x}{n}\right)}$$

$[\theta_{222}]$ is the mean residue ellipticity of the peptide at 222 nm, $[\theta_{\infty}]_{222}$ is the assumed mean residue ellipticity for a peptide with 100% helicity (−44,000 deg cm^2 $dmol^{-1}$), n is the number of residues within the tested peptide sequence, and x is an empirical correction for end effects.

<u>HPLC Traces for *S. mitis*-CSP-2 analogs</u>

S. mitis-CSP-2 (synthesized)

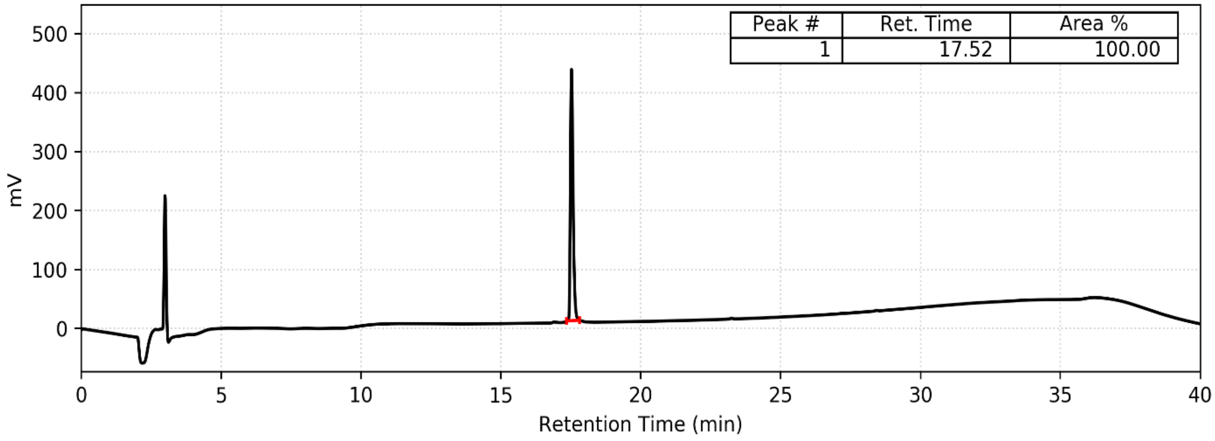

S. mitis-CSP-2 (extracted)

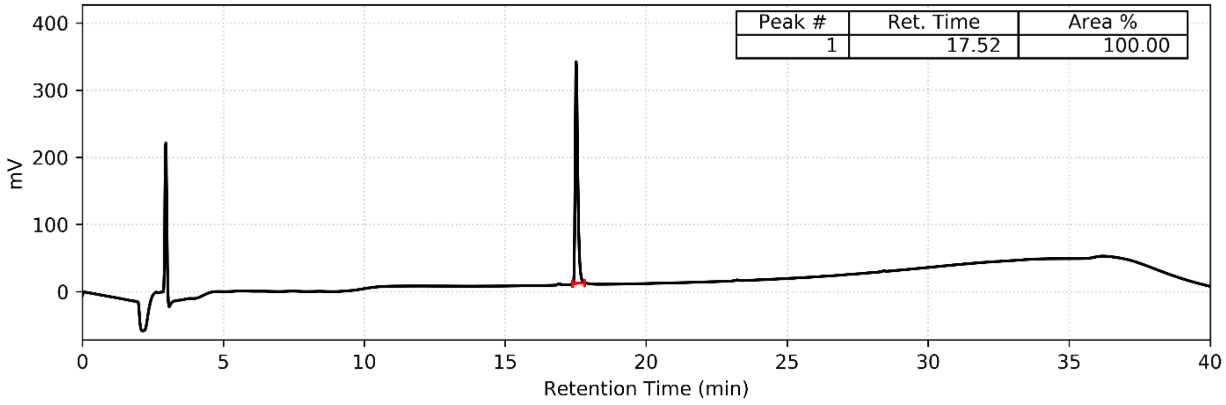

S. mitis-CSP-2-E1A

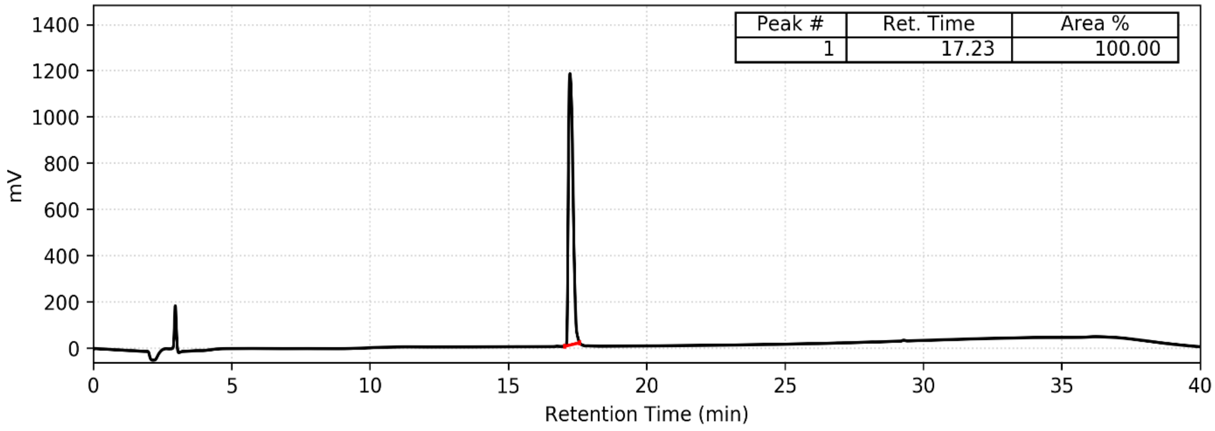

S. mitis-CSP-2-I2A

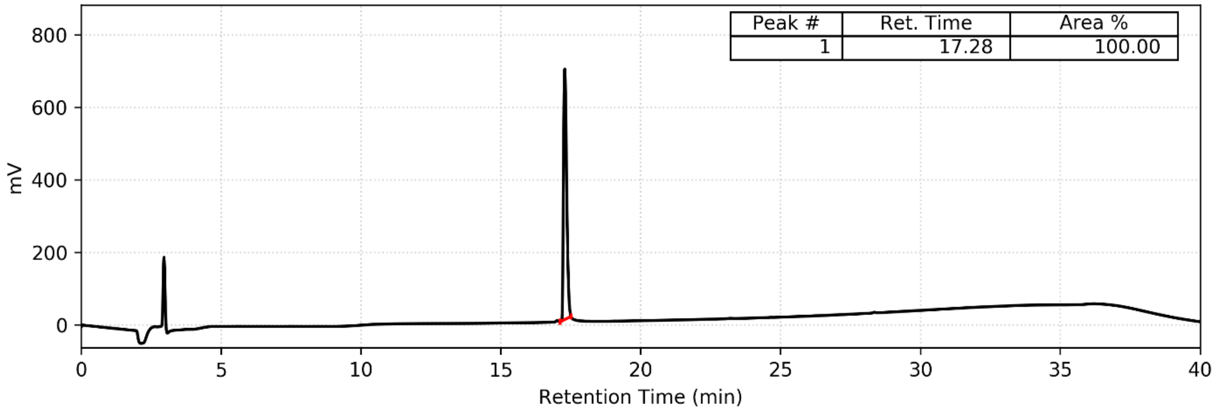

Peak #	Ret. Time	Area %
1	17.28	100.00

S. mitis-CSP-2-R3A

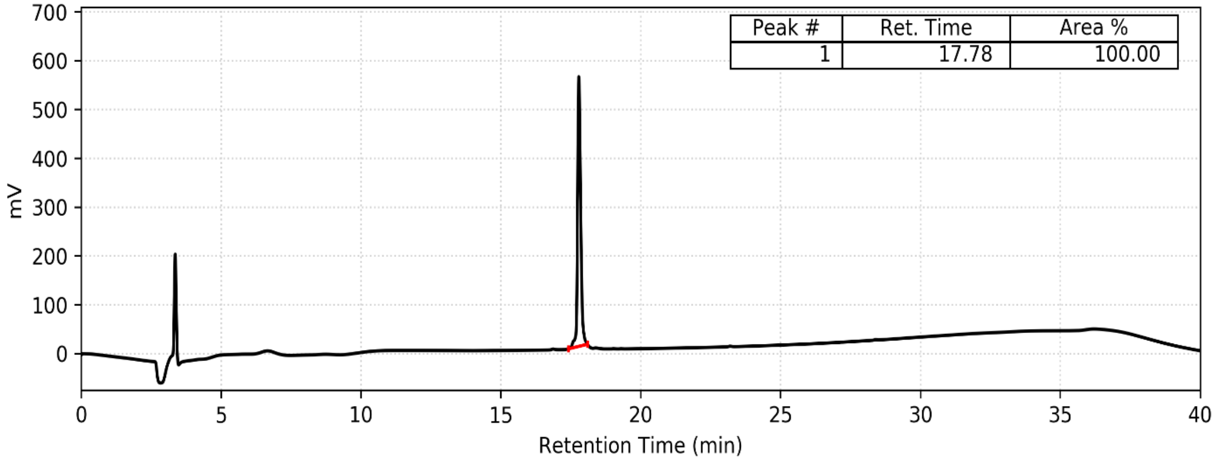

Peak #	Ret. Time	Area %
1	17.78	100.00

S. mitis-CSP-2-Q4A

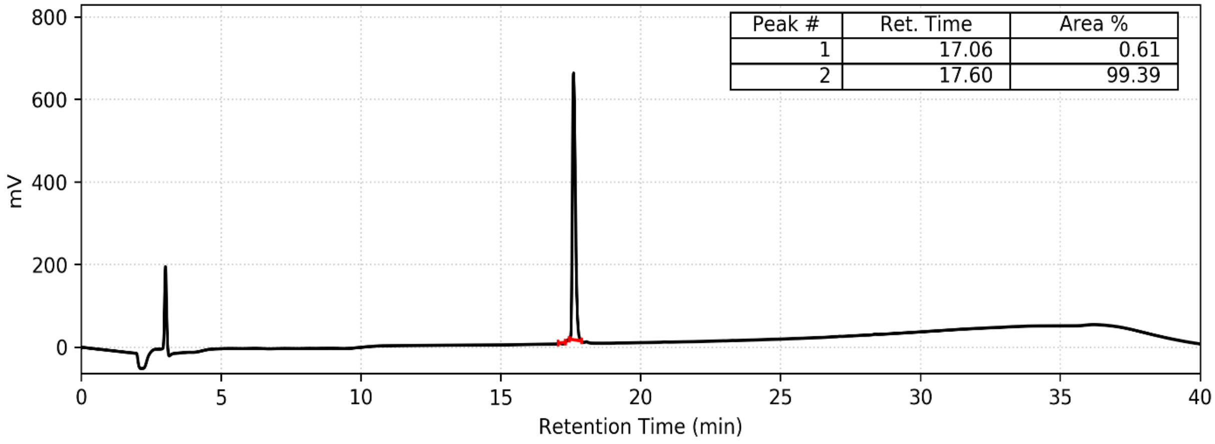

Peak #	Ret. Time	Area %
1	17.06	0.61
2	17.60	99.39

S. mitis-CSP-2-T5A

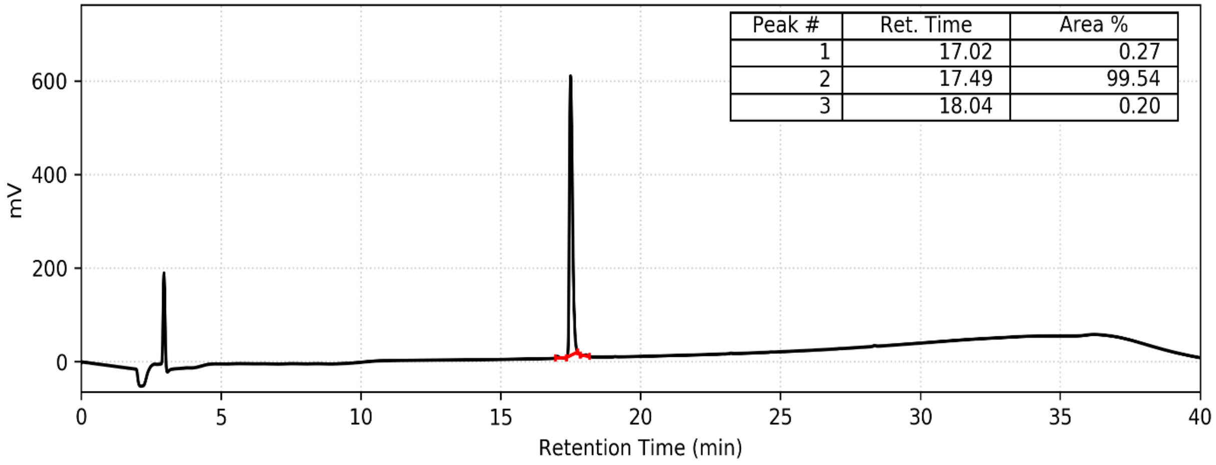

S. mitis-CSP-2-H6A

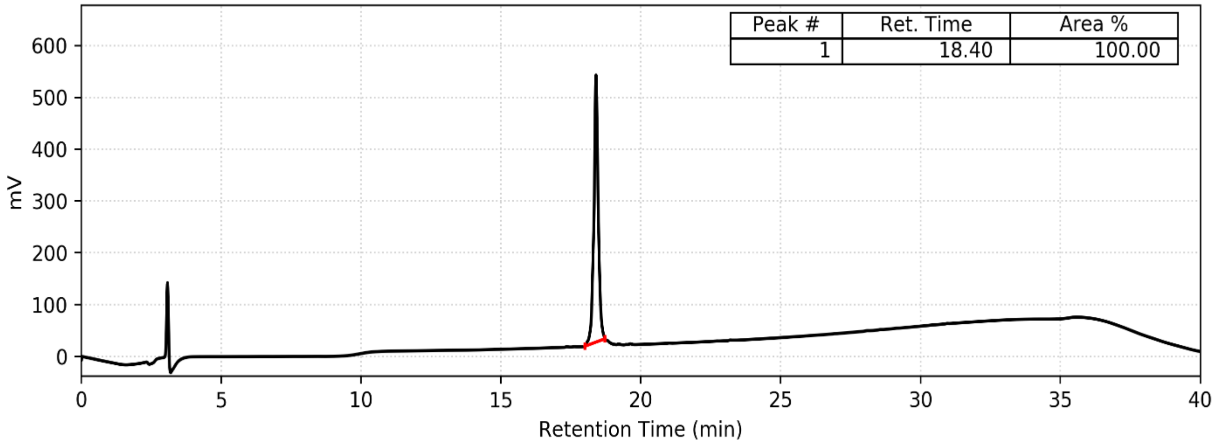

S. mitis-CSP-2-N7A

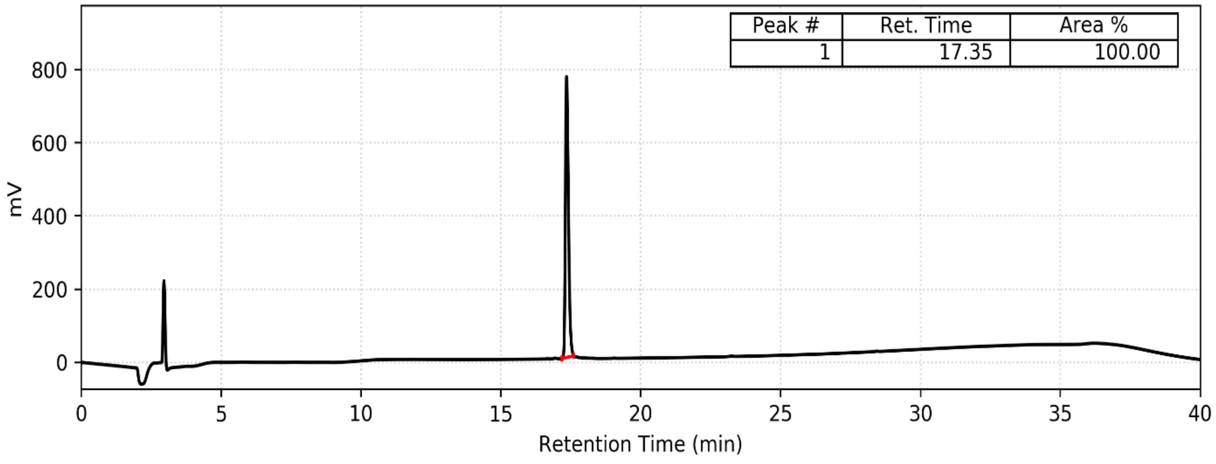

S. mitis-CSP-2-I8A

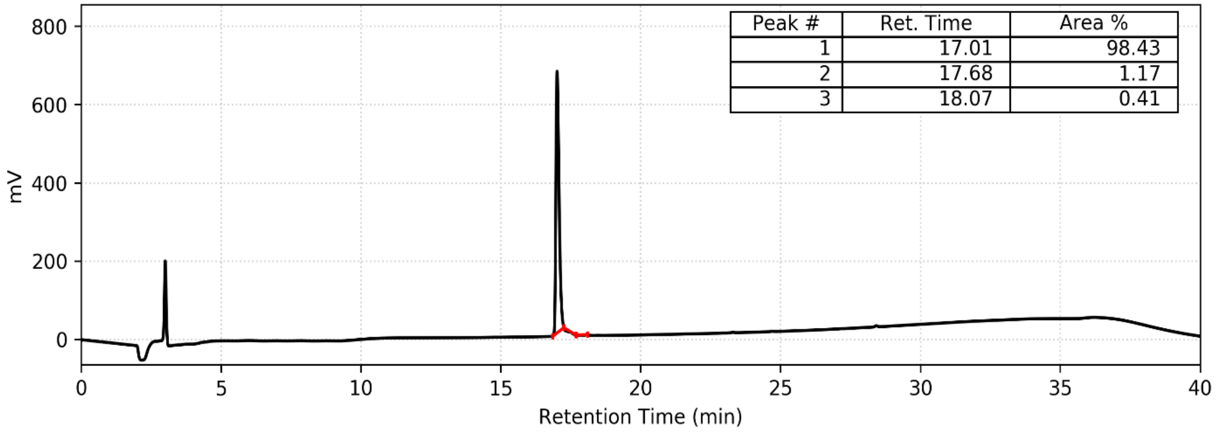

S. mitis-CSP-2-F9A

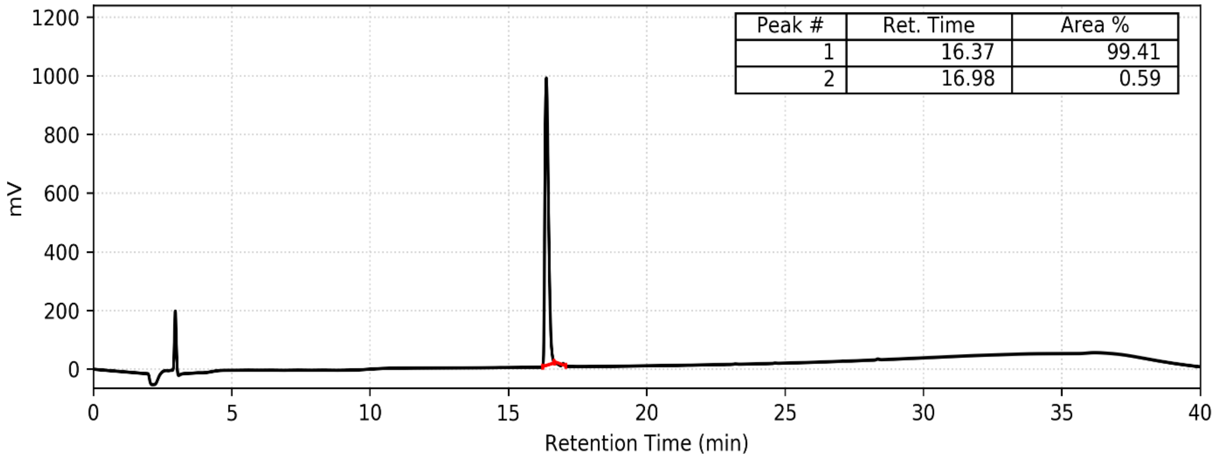

S. mitis-CSP-2-F10A

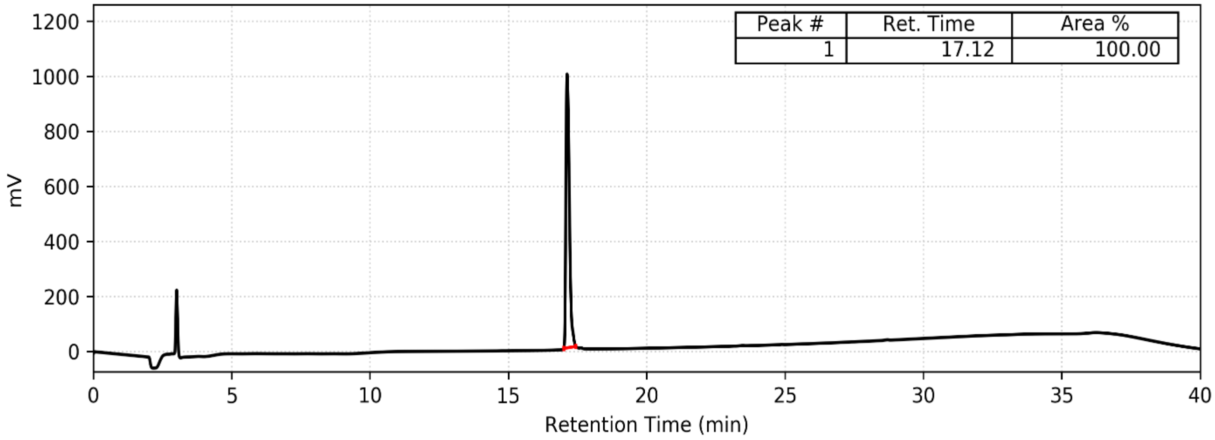

S. mitis-CSP-2-N11A

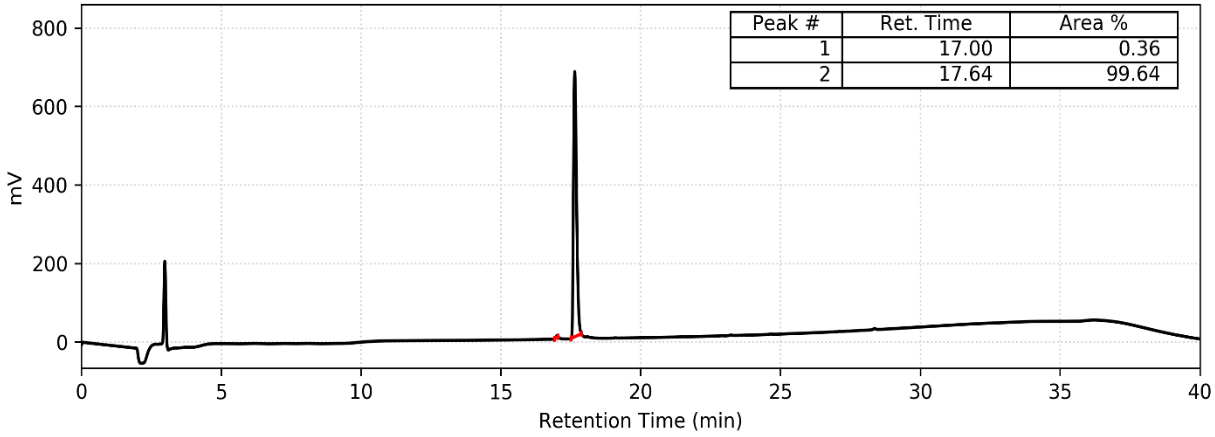

S. mitis-CSP-2-F12A

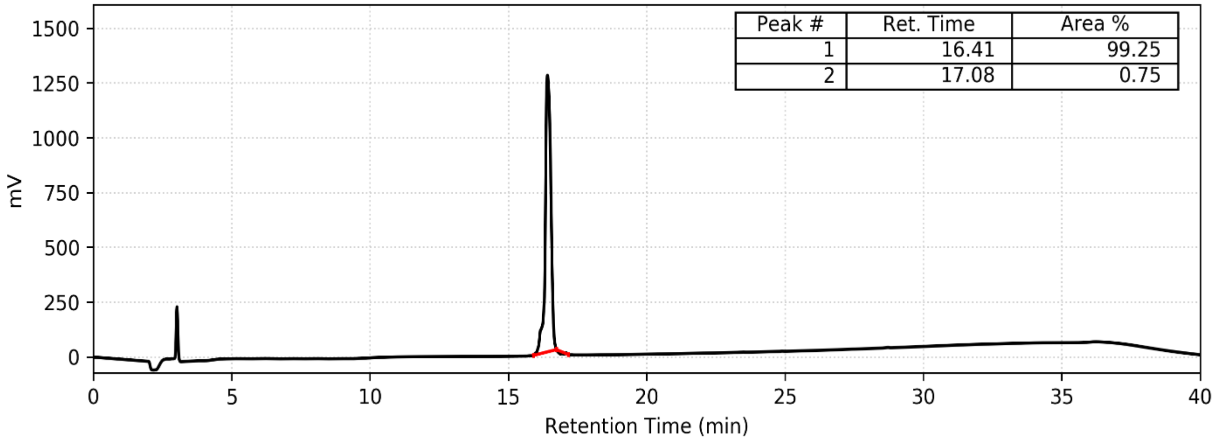

S. mitis-CSP-2-F13A

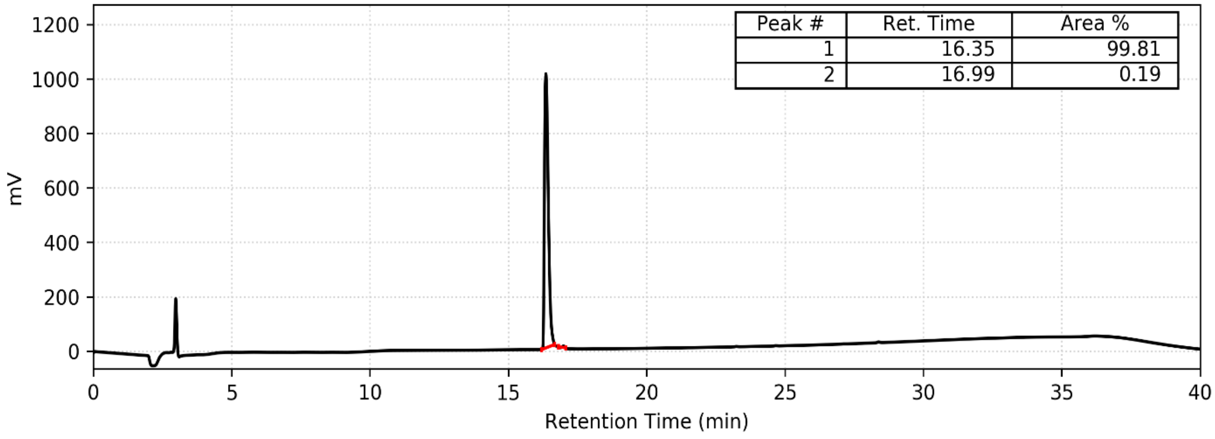

S. mitis-CSP-2-K14A

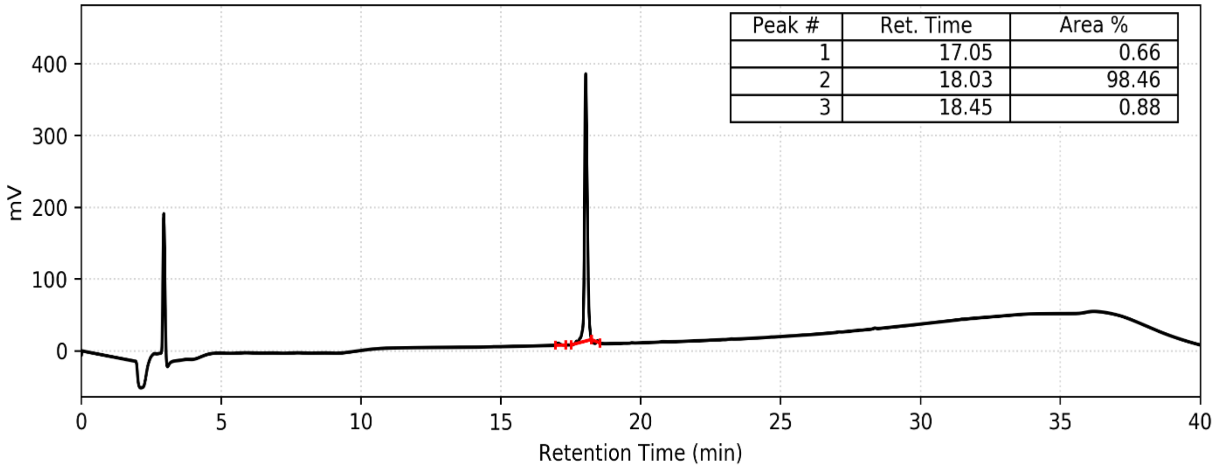

S. mitis-CSP-2-R15A

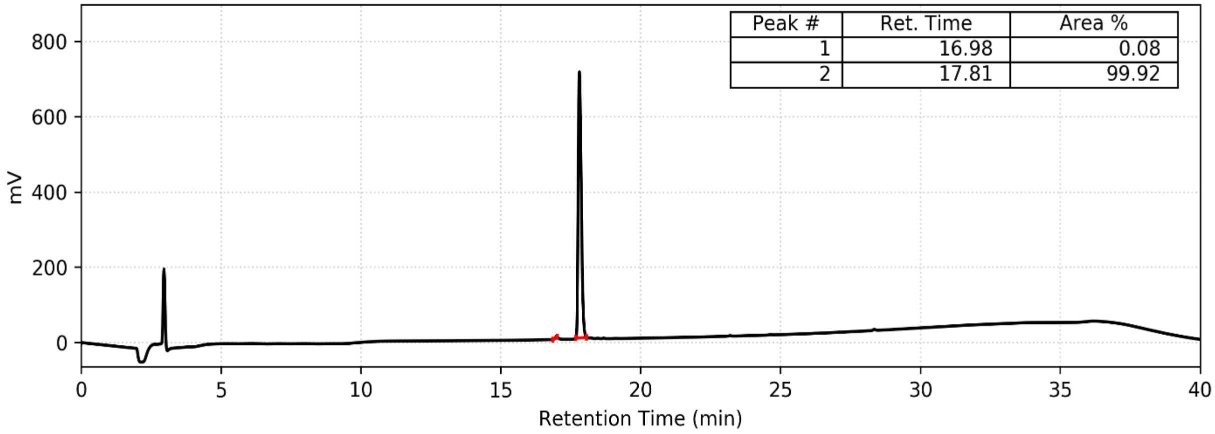

S. mitis-CSP-2-R16A

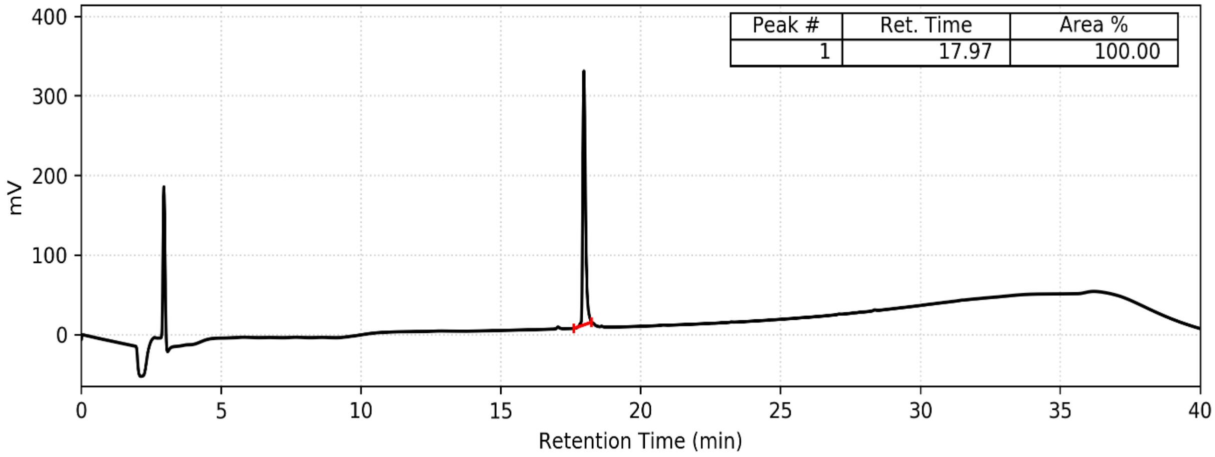

S. mitis-CSP-2-e1

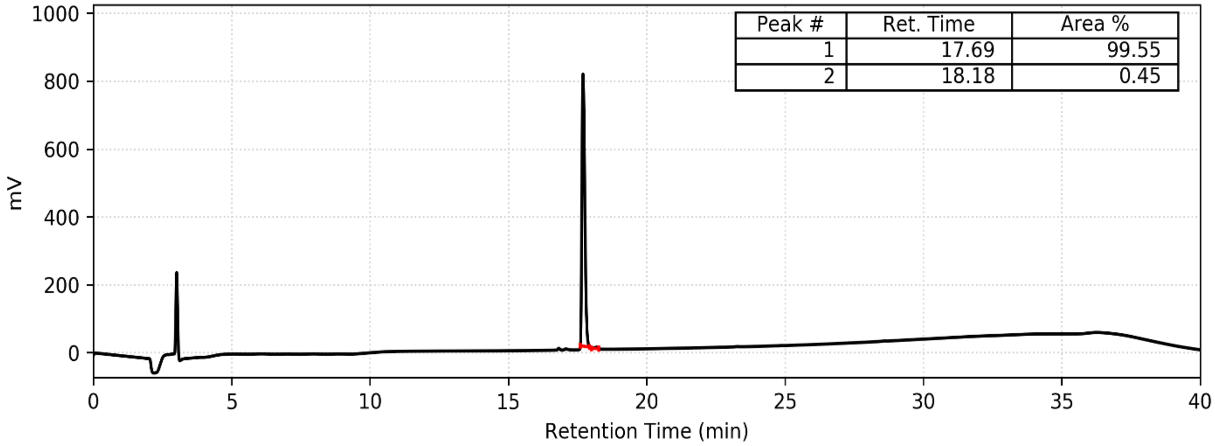

S. mitis-CSP-2-i2

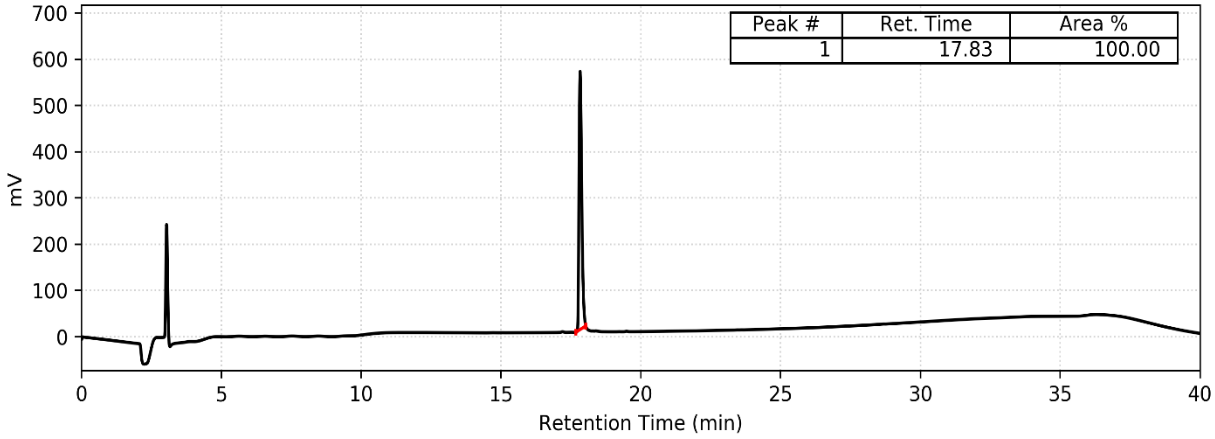

S. mitis-CSP-2-r3

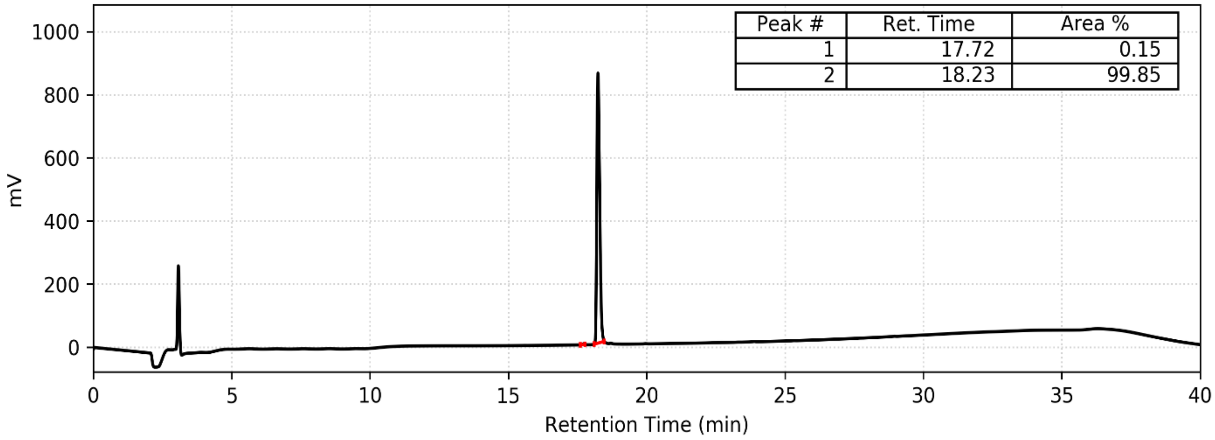

S. mitis-CSP-2-q4

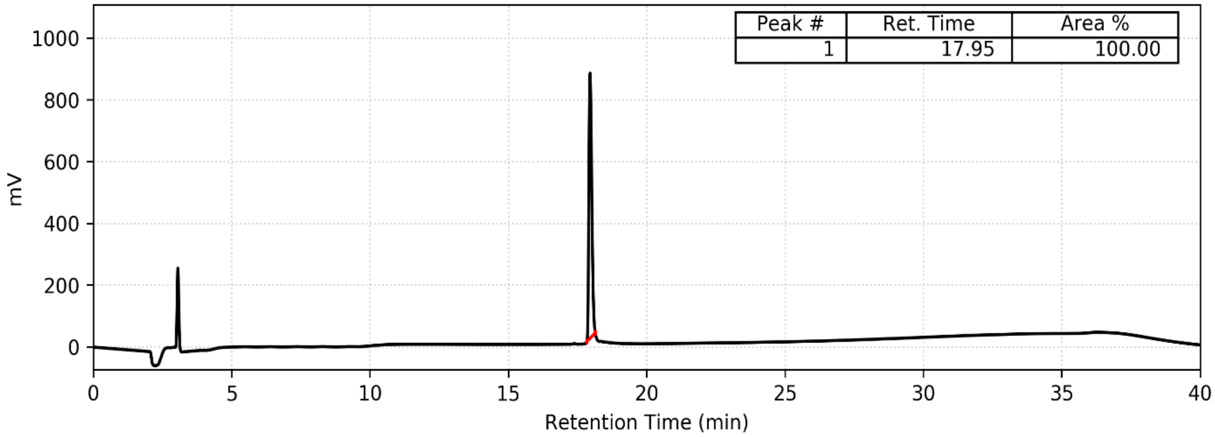

Peak #	Ret. Time	Area %
1	17.95	100.00

S. mitis-CSP-2-t5

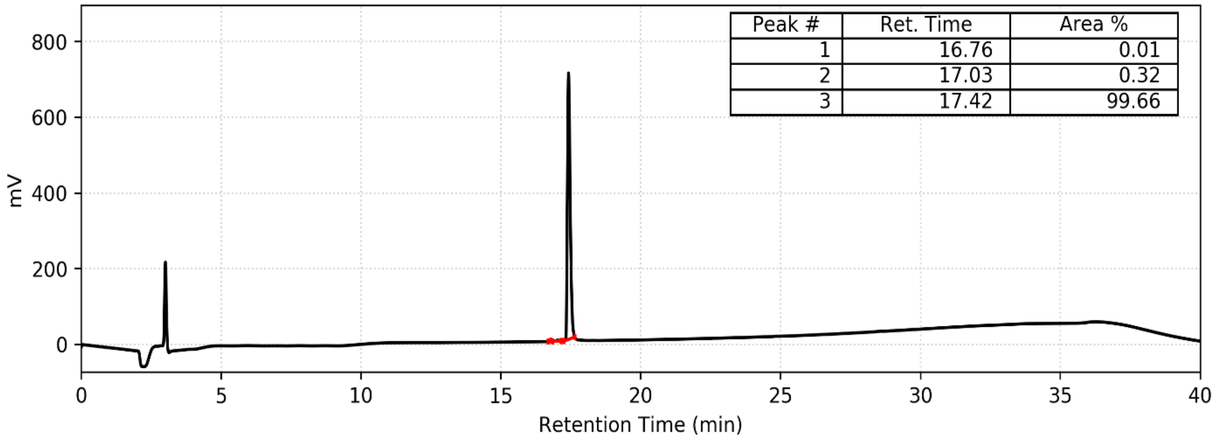

Peak #	Ret. Time	Area %
1	16.76	0.01
2	17.03	0.32
3	17.42	99.66

S. mitis-CSP-2-h6

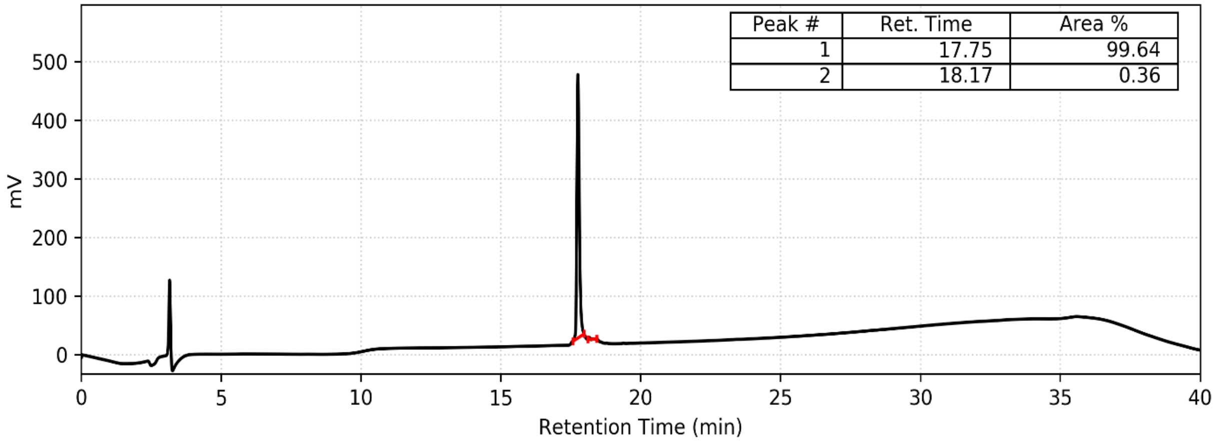

Peak #	Ret. Time	Area %
1	17.75	99.64
2	18.17	0.36

S. mitis-CSP-2-n7

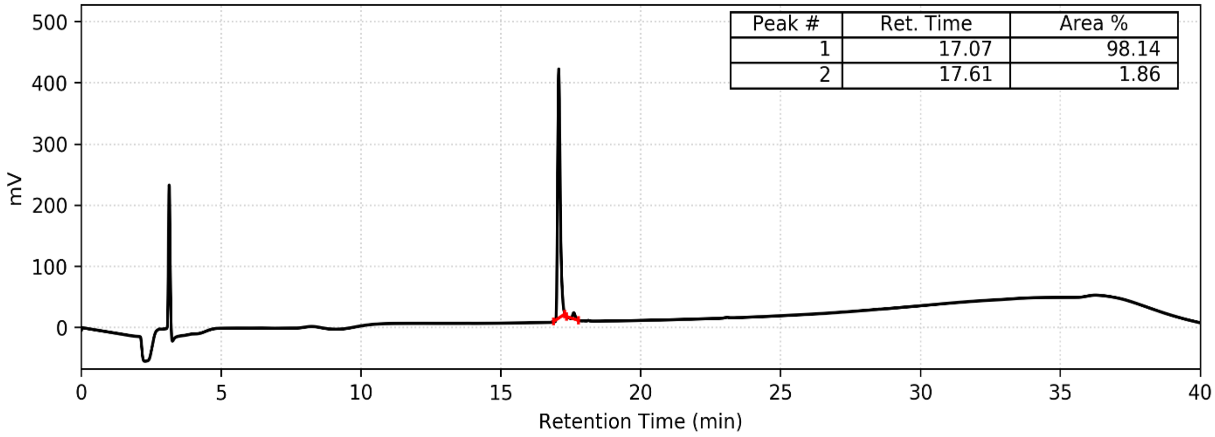

Peak #	Ret. Time	Area %
1	17.07	98.14
2	17.61	1.86

S. mitis-CSP-2-i8

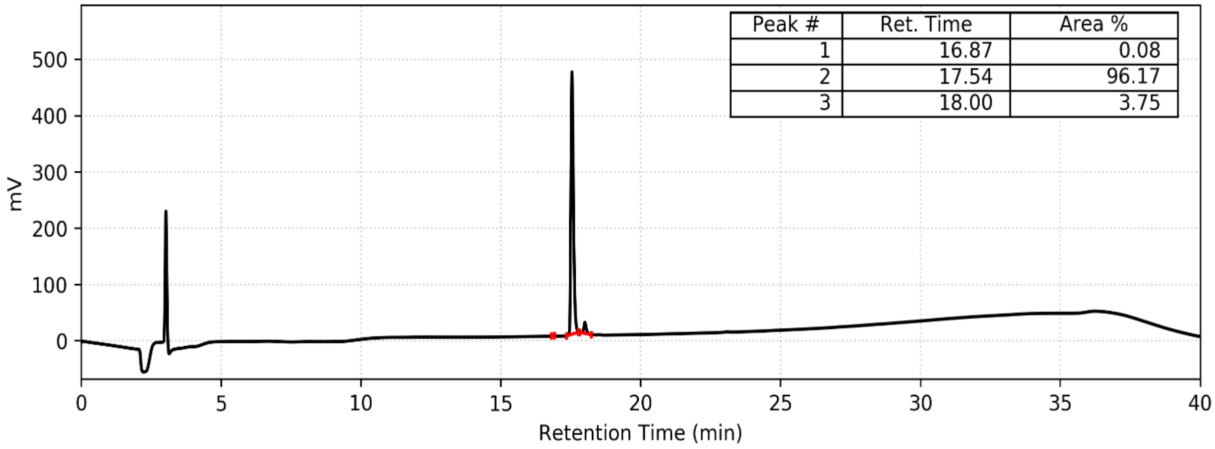

Peak #	Ret. Time	Area %
1	16.87	0.08
2	17.54	96.17
3	18.00	3.75

S. mitis-CSP-2-f9

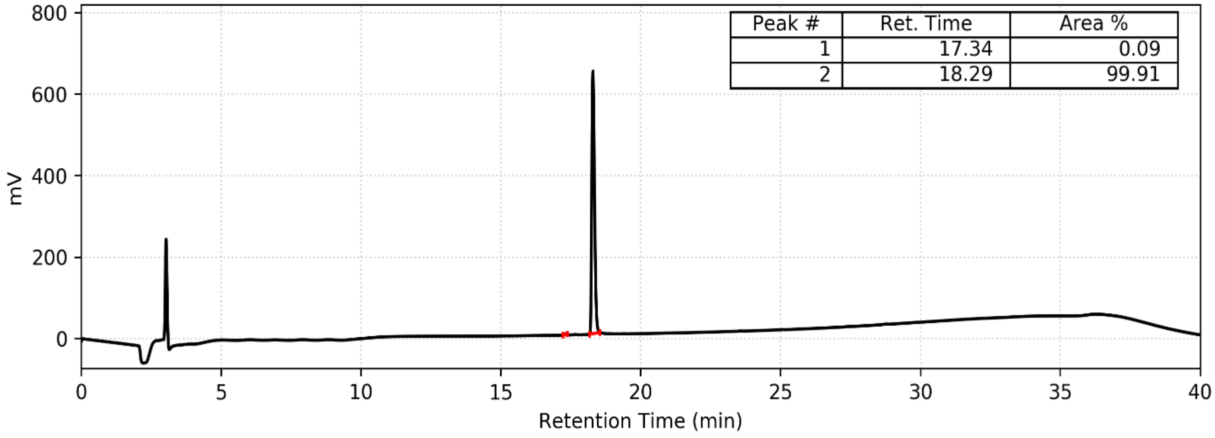

Peak #	Ret. Time	Area %
1	17.34	0.09
2	18.29	99.91

S. mitis-CSP-2-f10

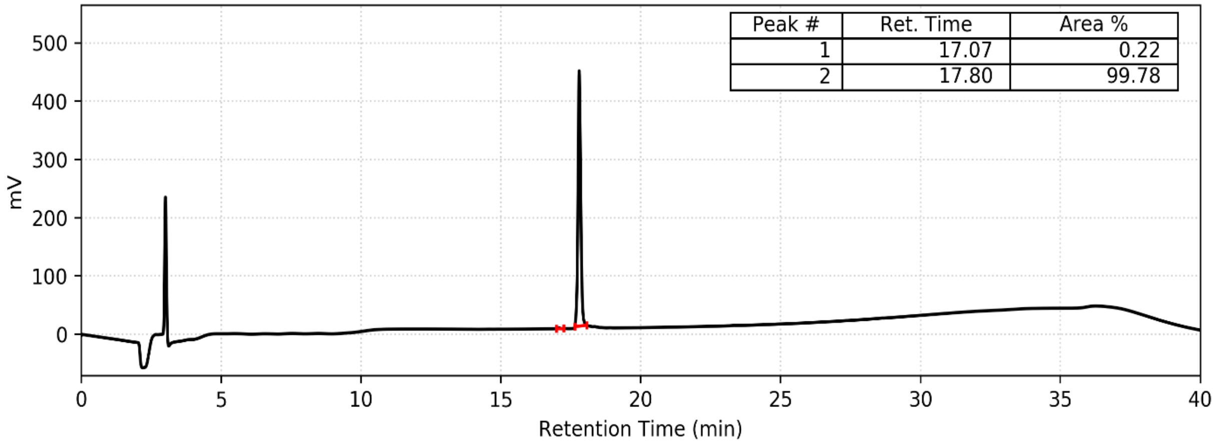

Peak #	Ret. Time	Area %
1	17.07	0.22
2	17.80	99.78

S. mitis-CSP-2-n11

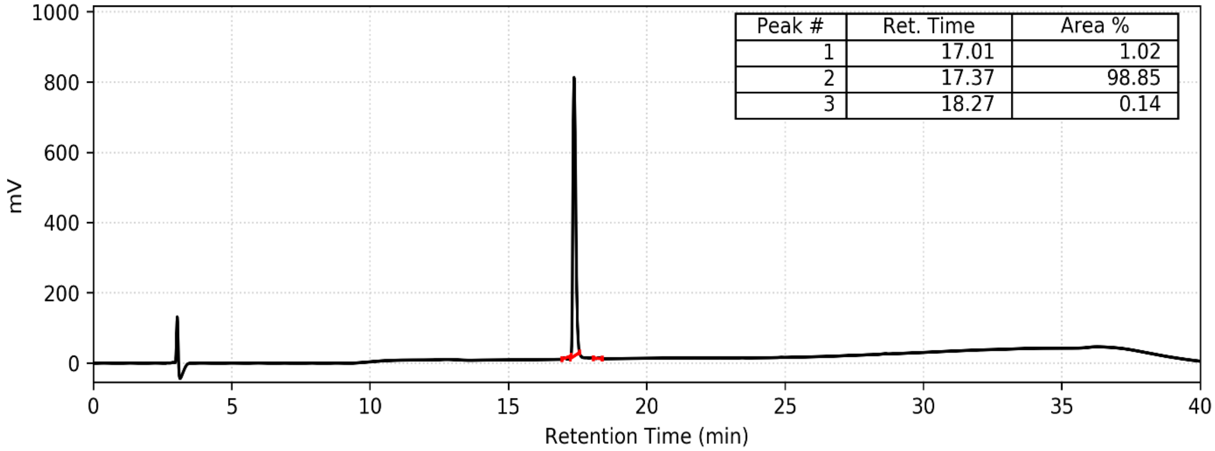

Peak #	Ret. Time	Area %
1	17.01	1.02
2	17.37	98.85
3	18.27	0.14

S. mitis-CSP-2-f12

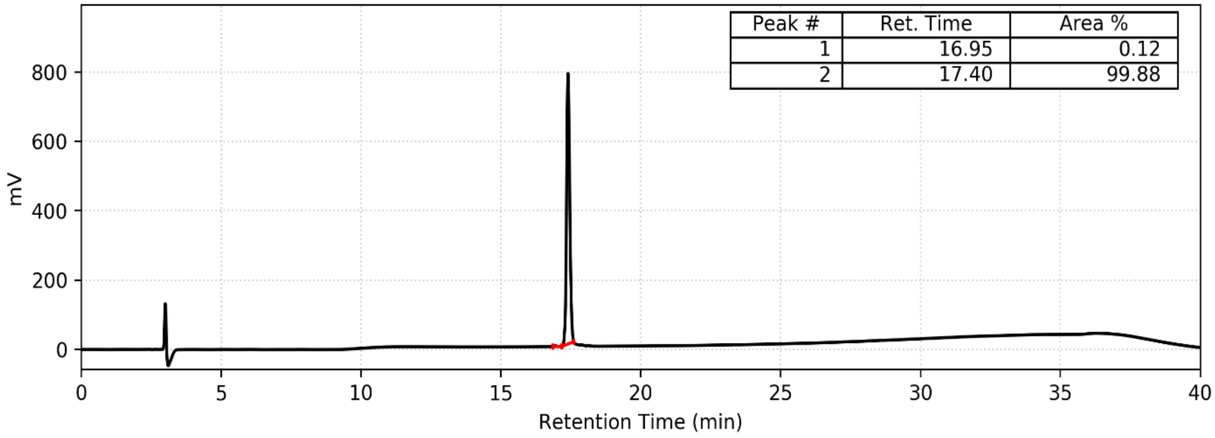

Peak #	Ret. Time	Area %
1	16.95	0.12
2	17.40	99.88

S. mitis-CSP-2-f13

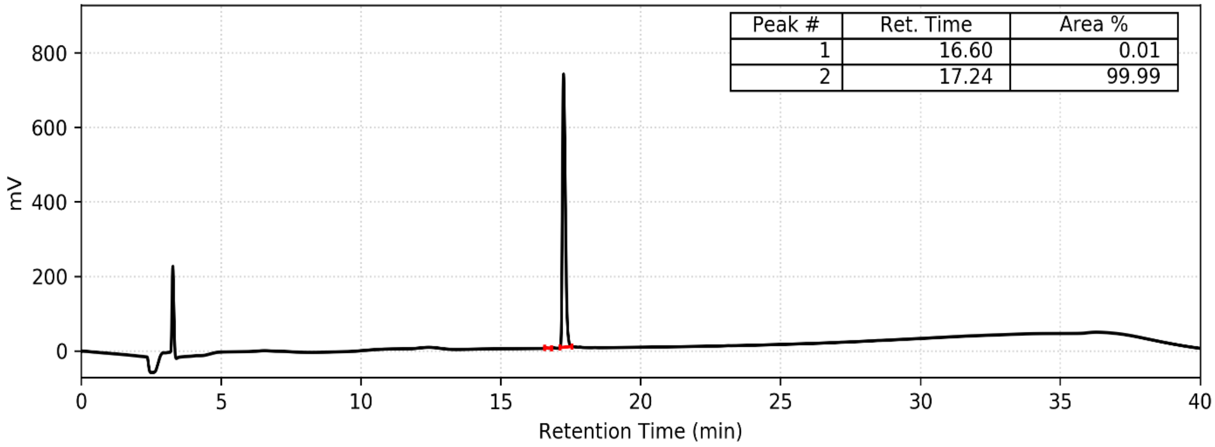

S. mitis-CSP-2-k14

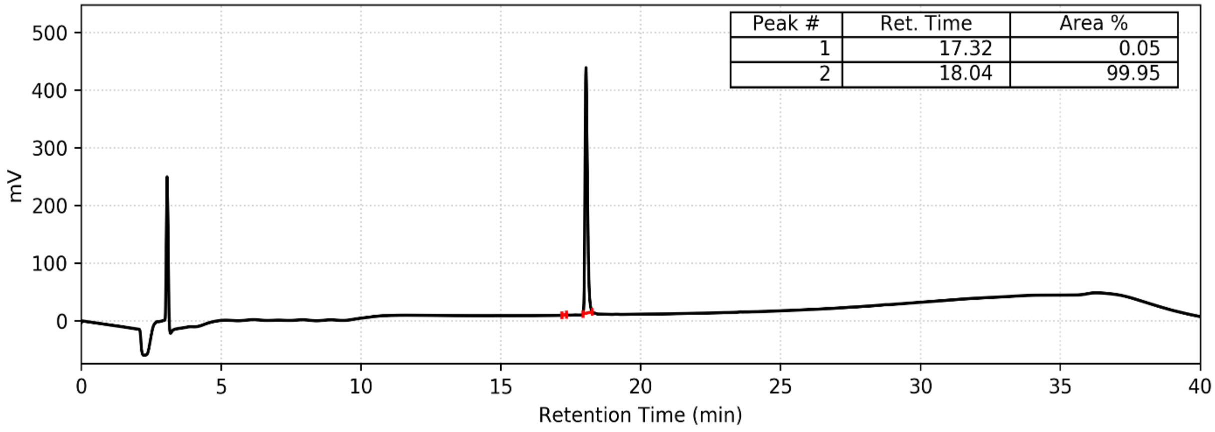

S. mitis-CSP-2-r15

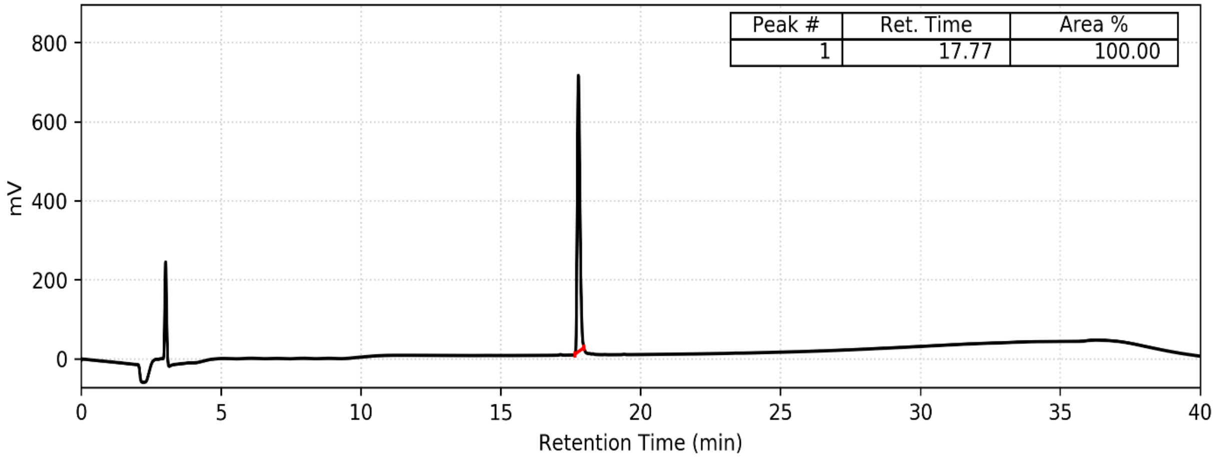

S. mitis-CSP-2-r16

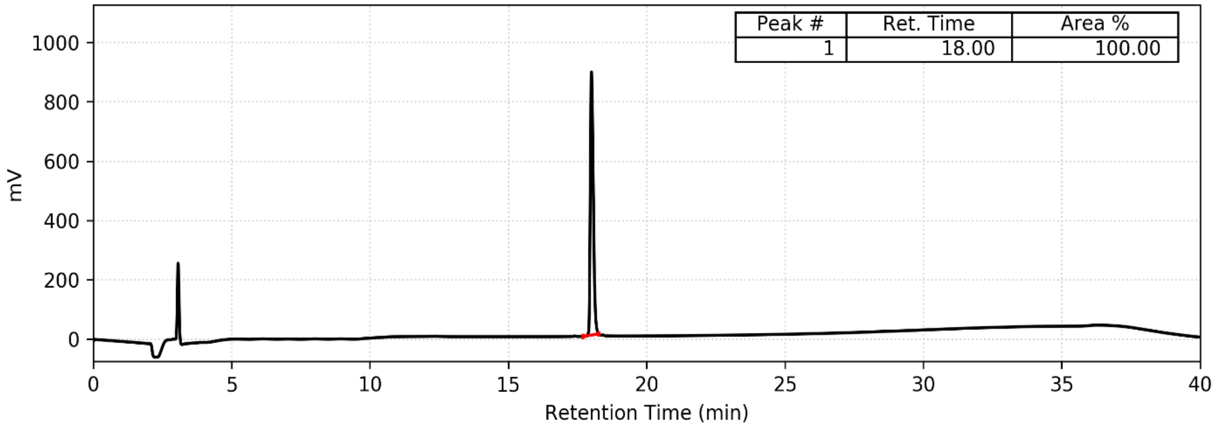

S. mitis-CSP-2-des-E1

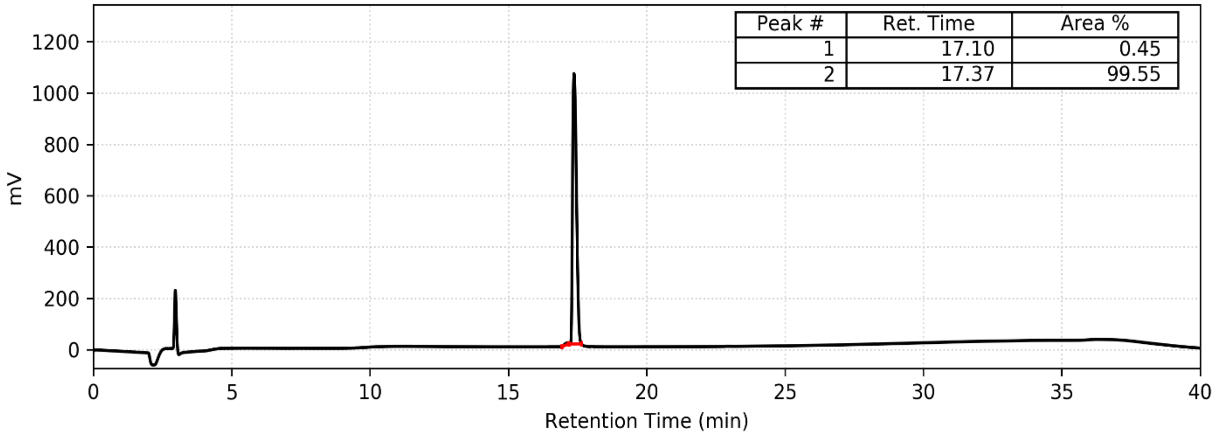

S. mitis-CSP-2-des-E1I2

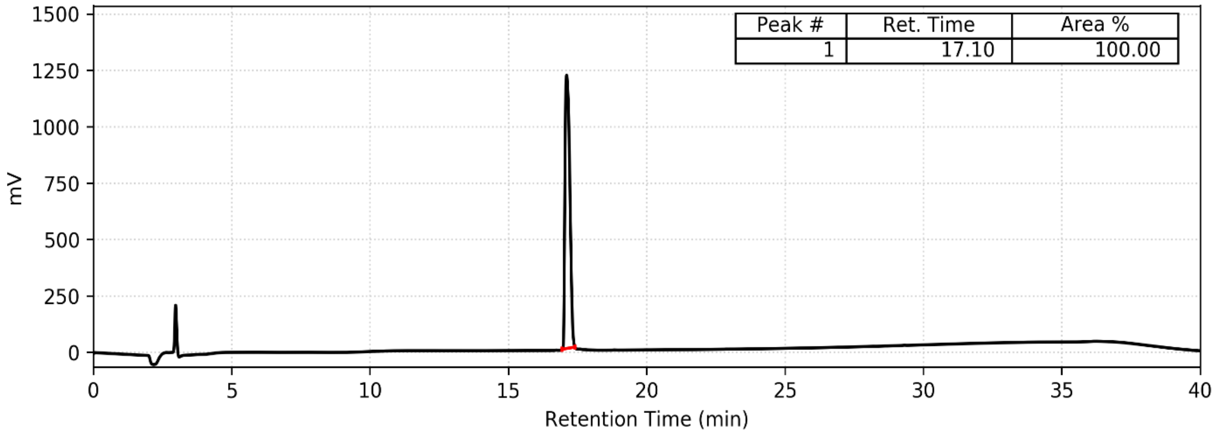

S. mitis-CSP-2-des-E1I2R3

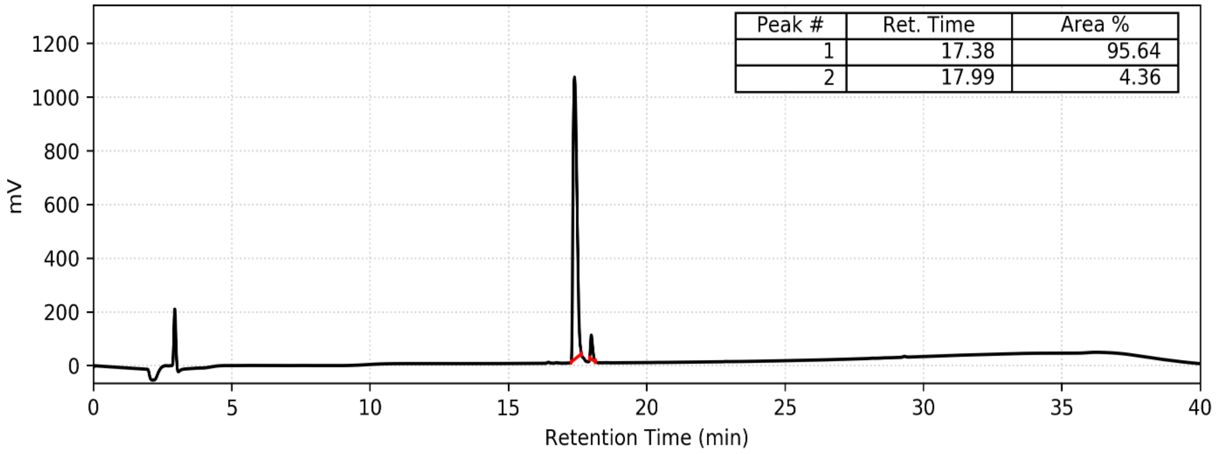

S. mitis-CSP-2-des-R16

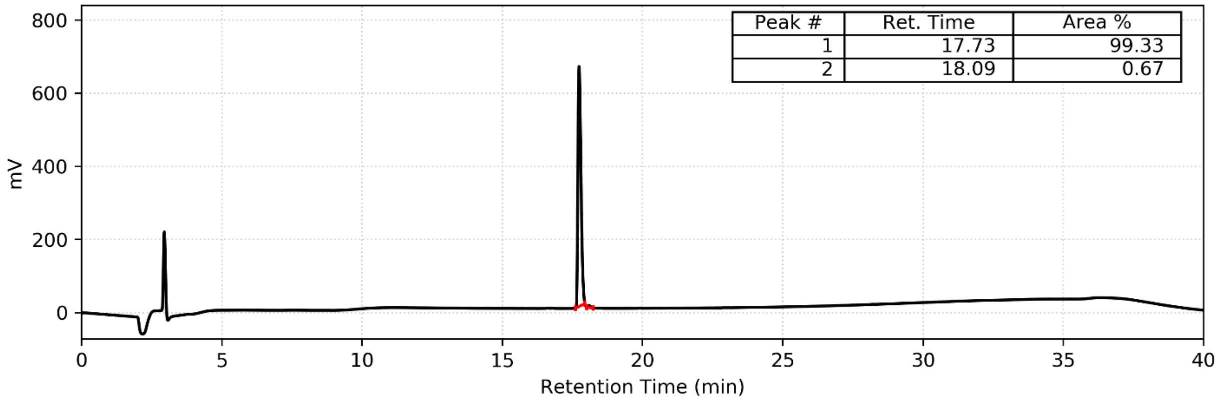

S. mitis-CSP-2-des-R15R16

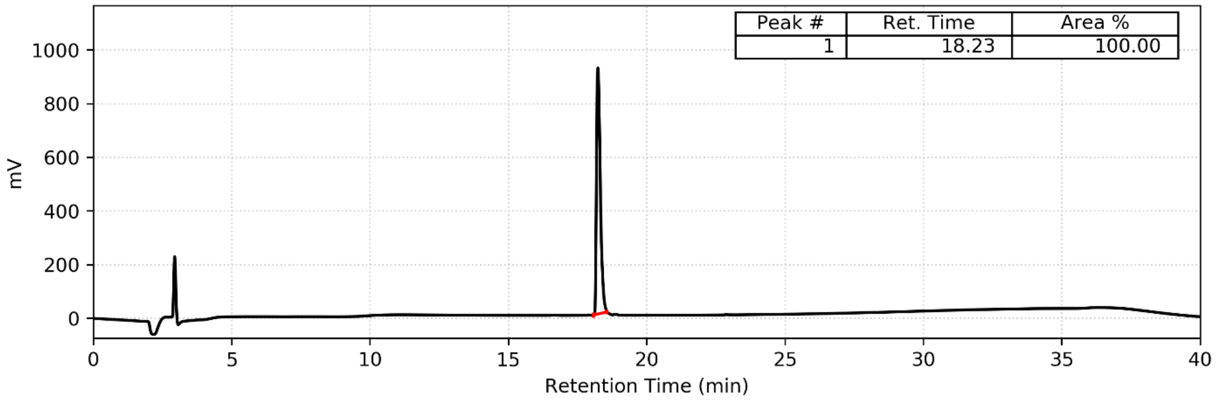

S. mitis-CSP-2-des-K14R15R16

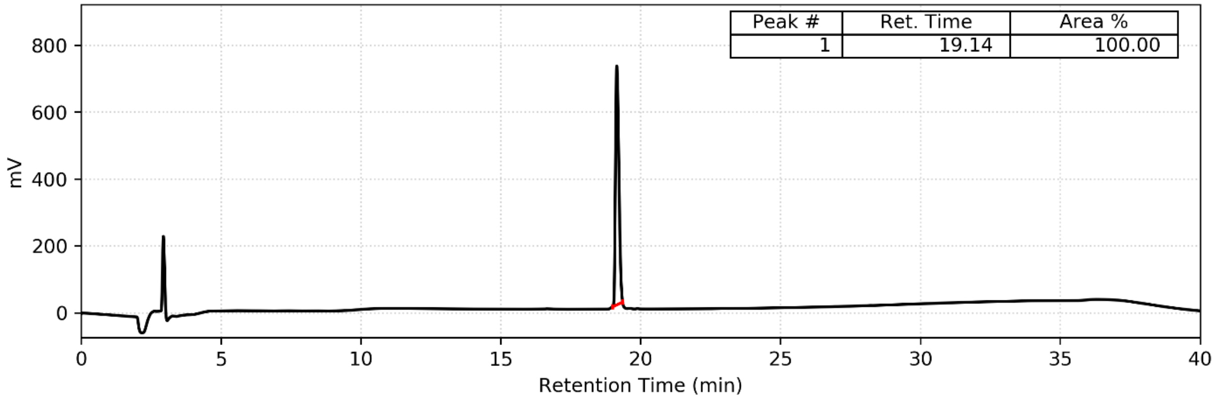

S. mitis-CSP-2-E1AN7A

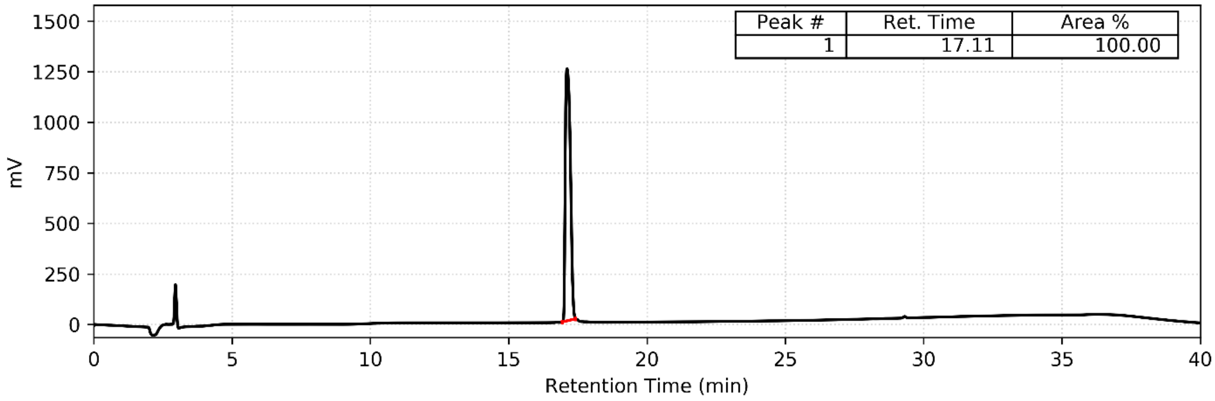

S. mitis-CSP-2-E1AN11A

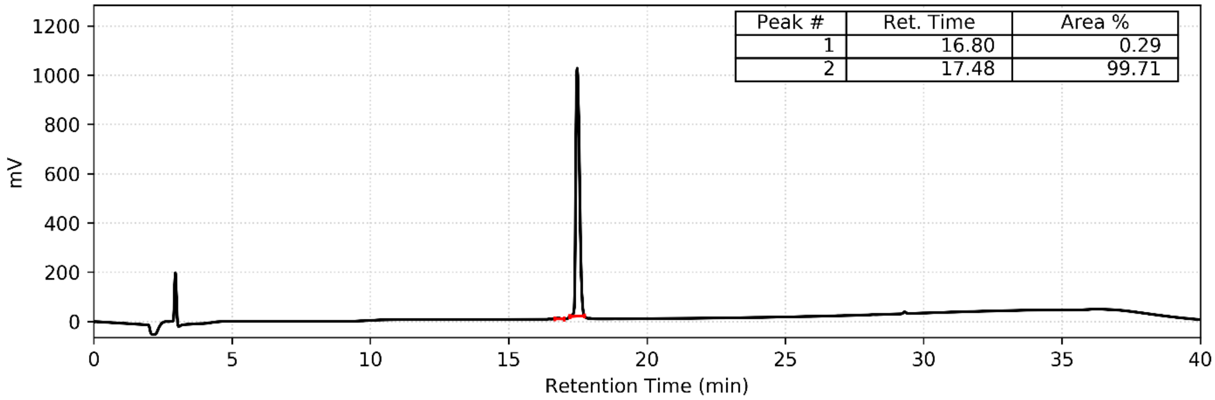

S. mitis-CSP-2-E1AF12A

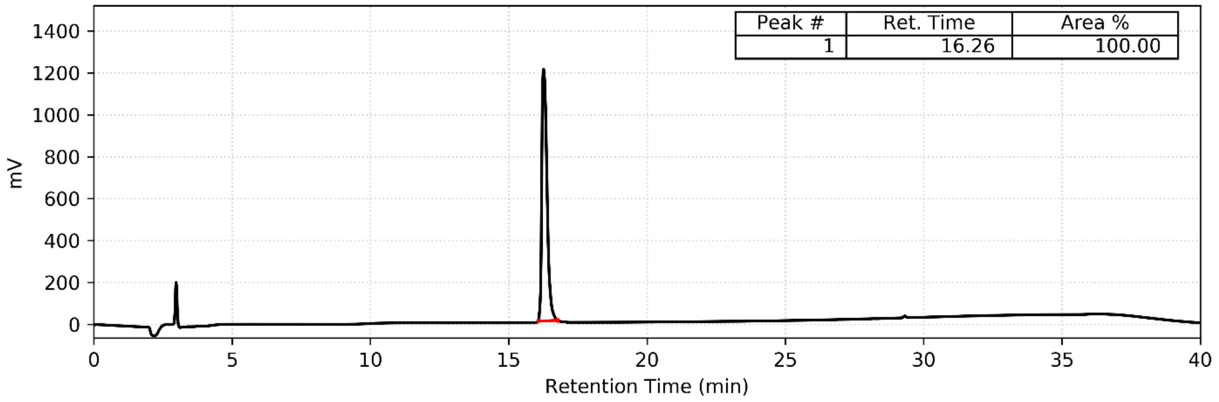

S. mitis-CSP-2-E1AF13A

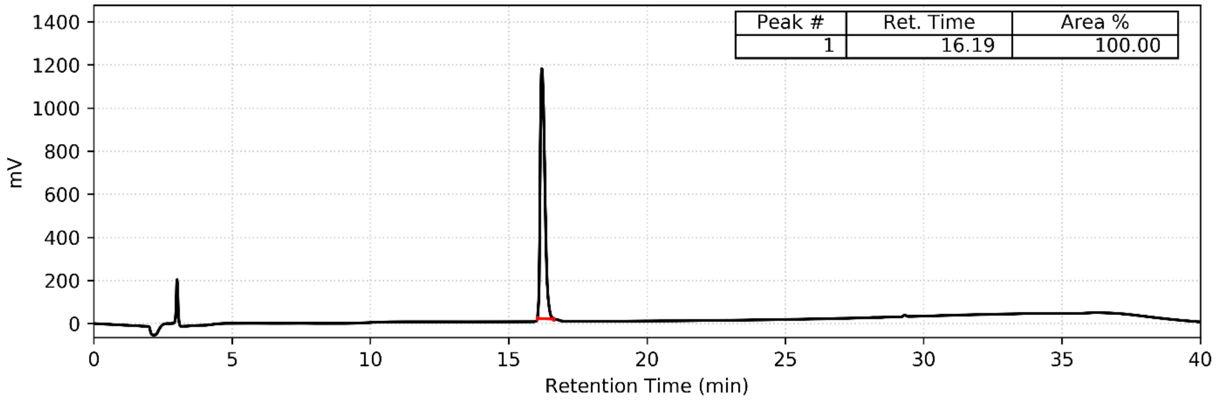

S. mitis-CSP-2-E1Ai8

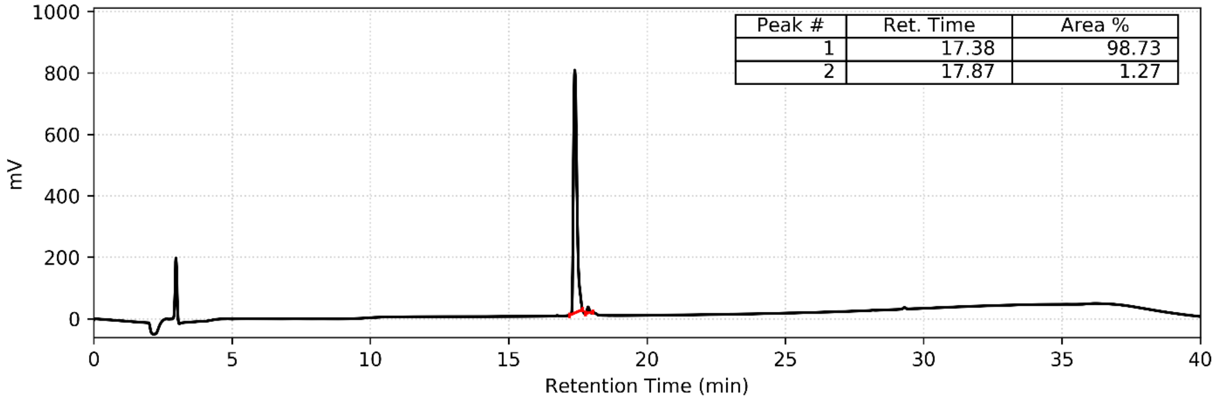

S. mitis-CSP-2-E1Af10

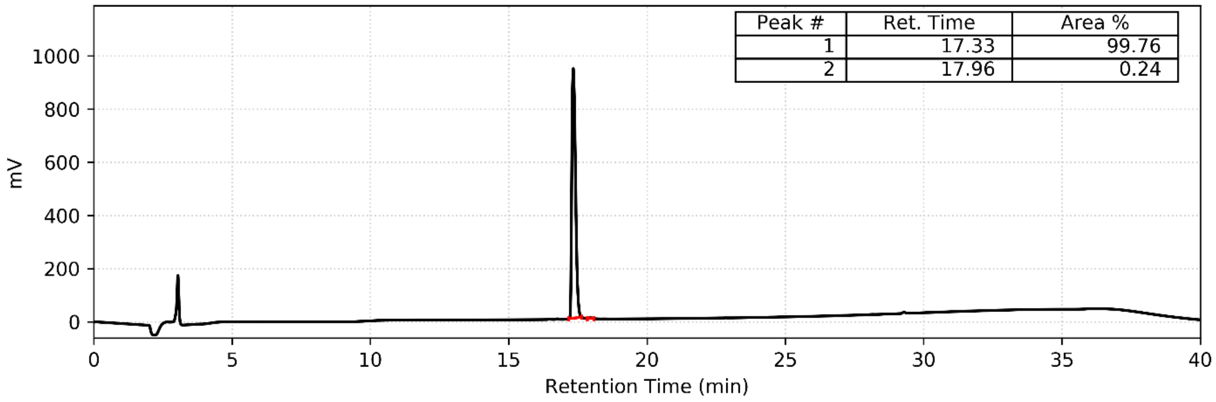

S. mitis-CSP-2-E1An11

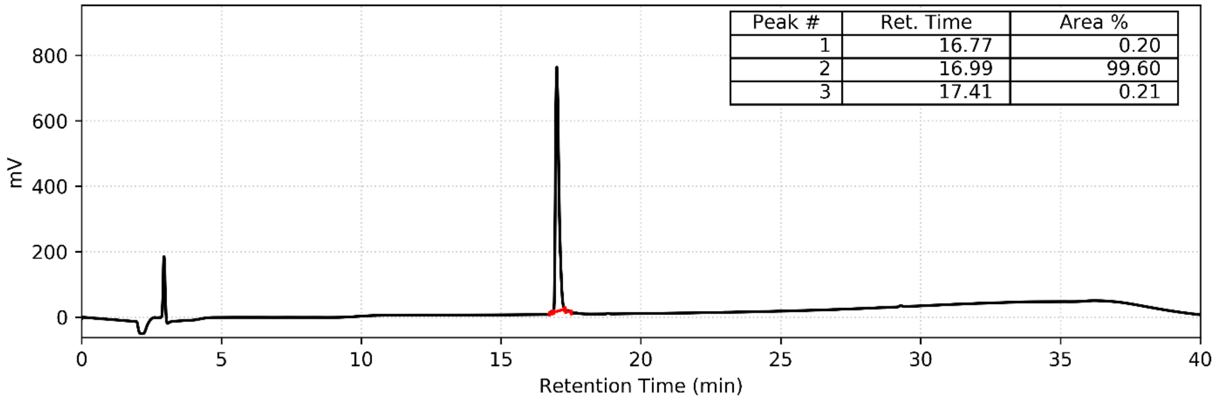

S. mitis-CSP-2-E1Ak14

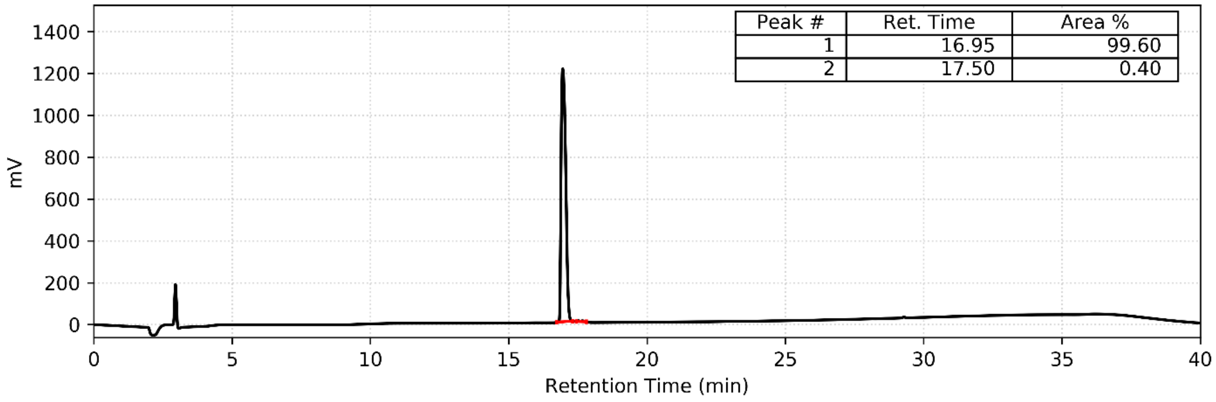

S. mitis-CSP-2-E1Ar16

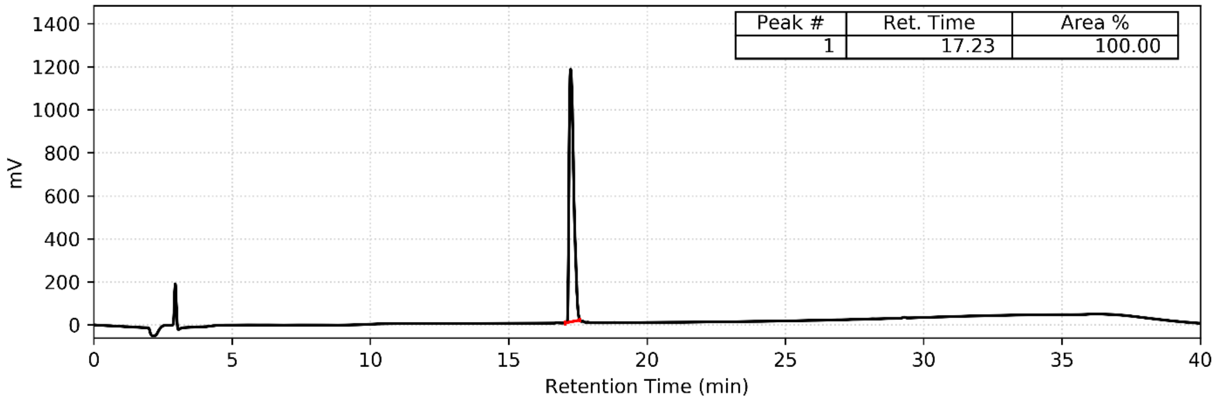

S. mitis-CSP-2-E1A-des-R16

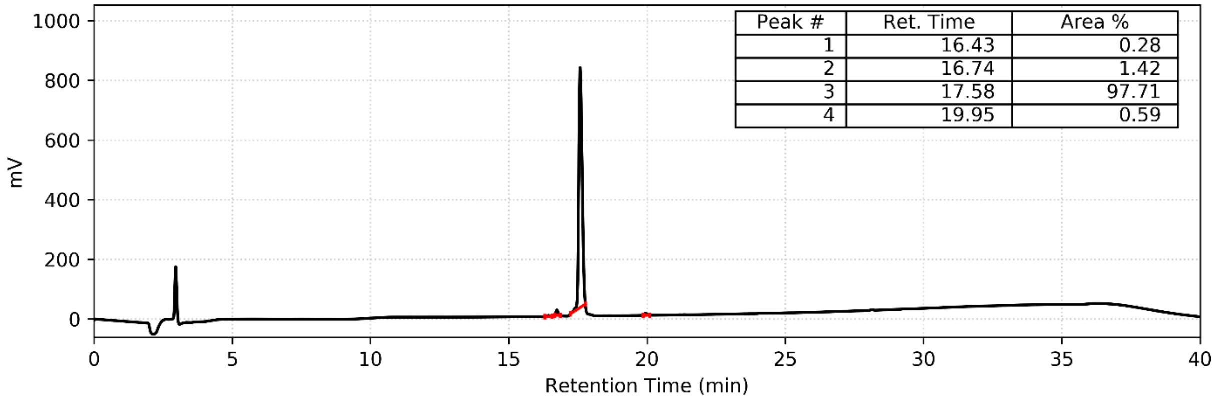

S. mitis-CSP-2-E1AN7Af10

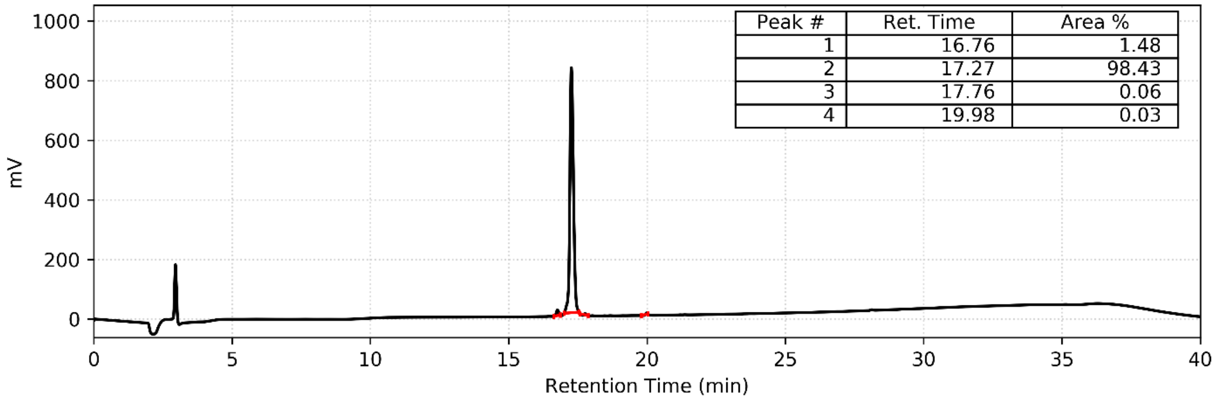

S. mitis-CSP-2-E1AN7An11

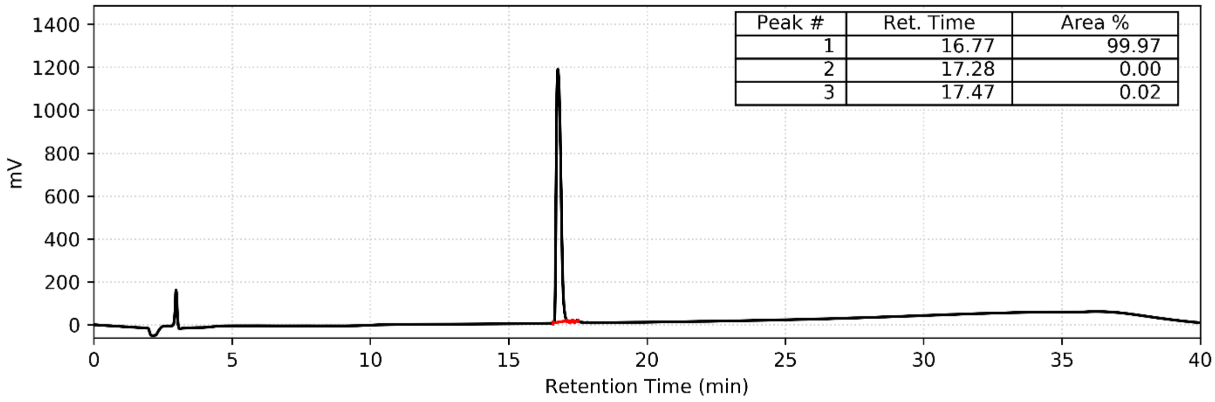

S. mitis-CSP-2-E1AN7Ak14

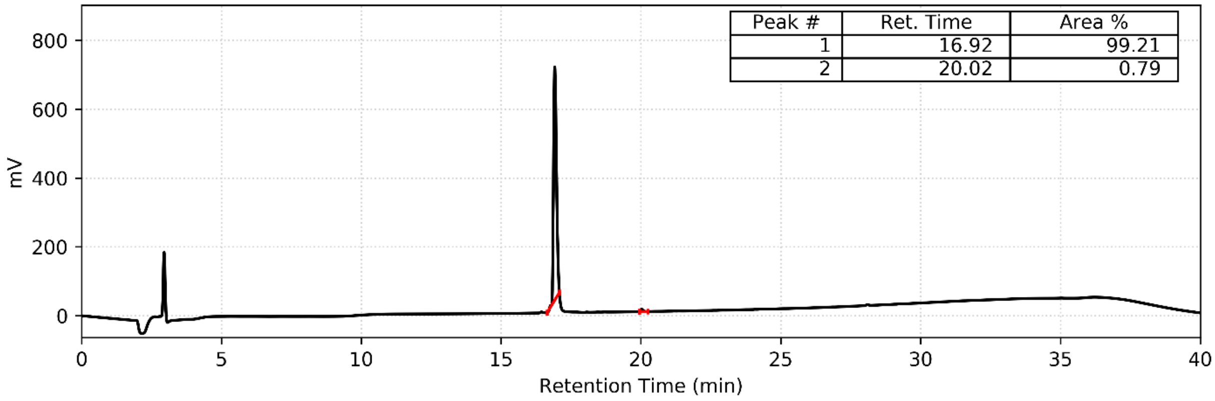

S. mitis-CSP-2-E1AN7Ar16

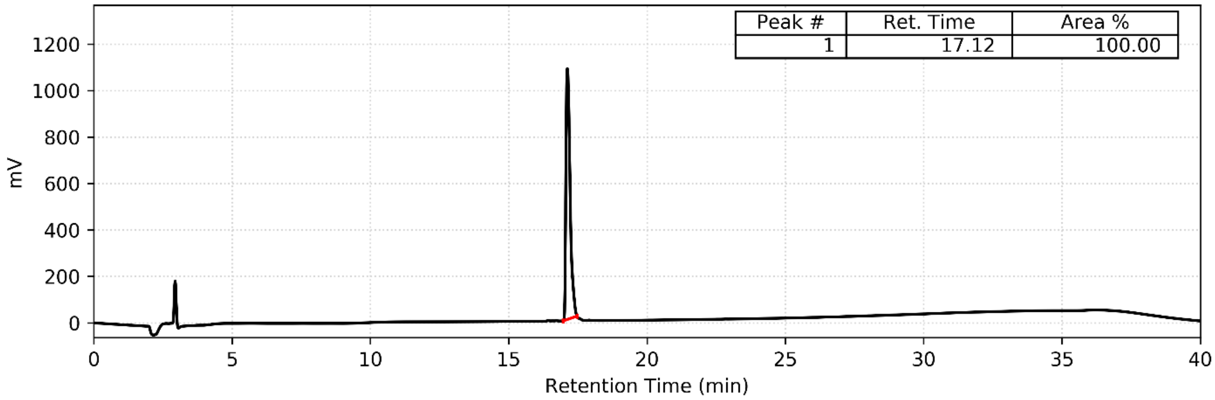

S. mitis-CSP-2-E1Af10n11

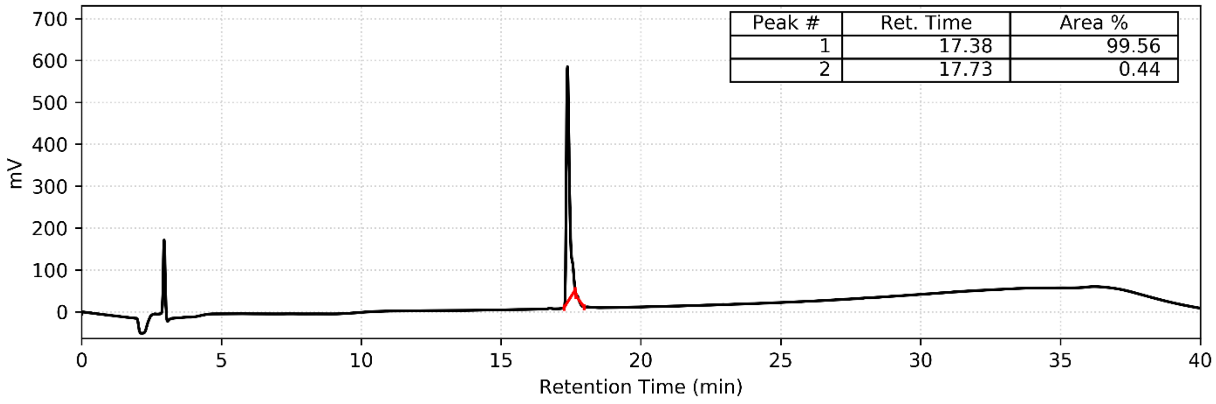

S. mitis-CSP-2-E1Af10k14

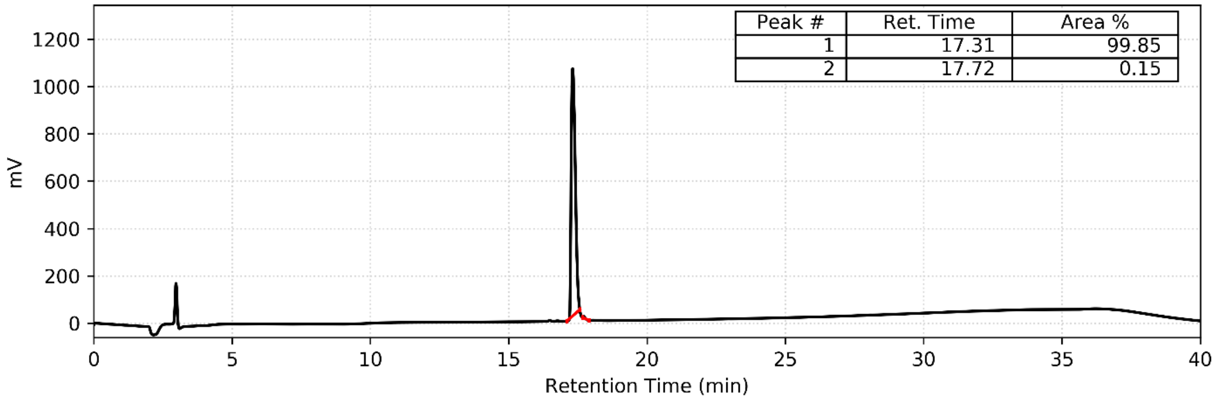

S. mitis-CSP-2-E1Af10r16

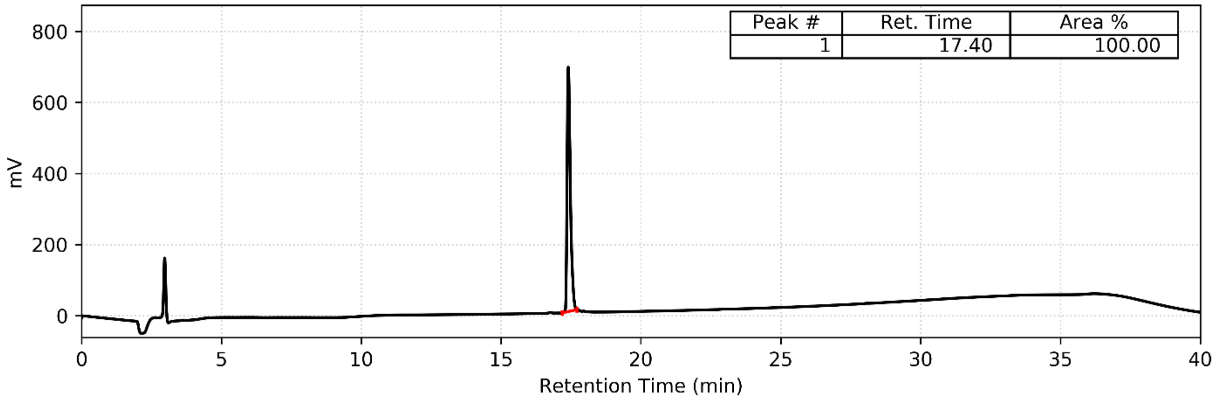

S. mitis-CSP-2-E1An11k14

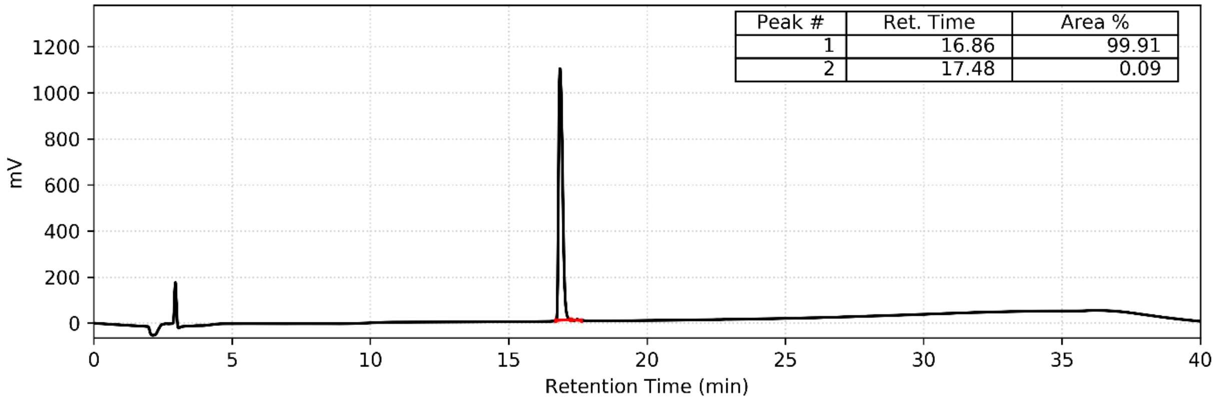

Peak #	Ret. Time	Area %
1	16.86	99.91
2	17.48	0.09

S. mitis-CSP-2-E1An11r16

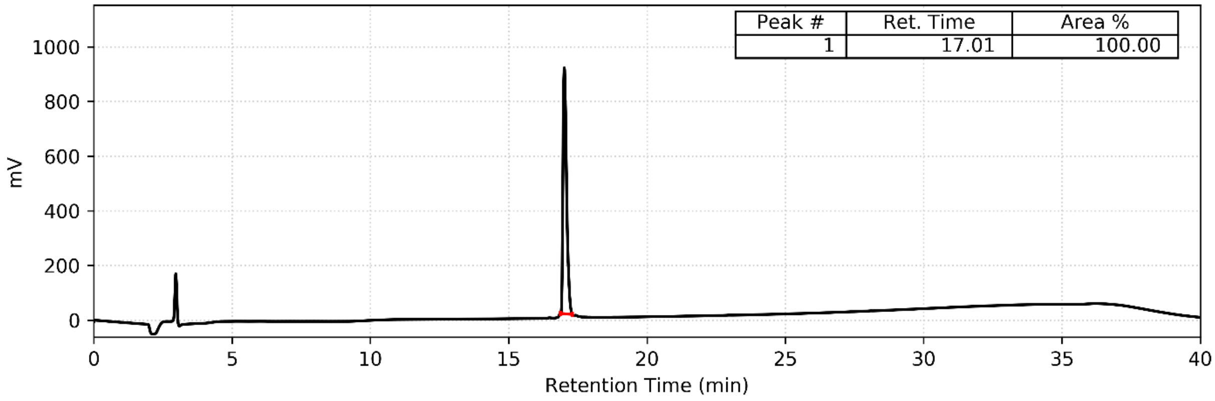

Peak #	Ret. Time	Area %
1	17.01	100.00

S. mitis-CSP-2-E1Ak14r16

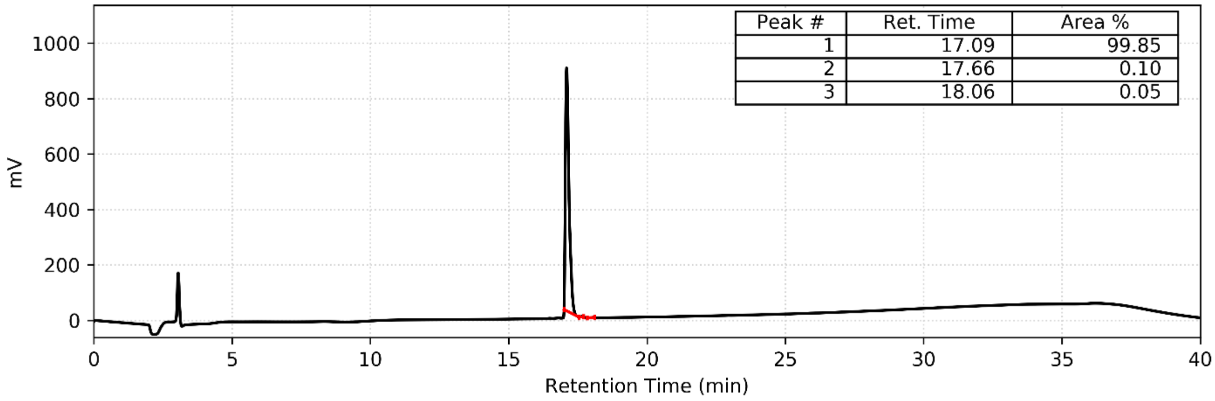

Peak #	Ret. Time	Area %
1	17.09	99.85
2	17.66	0.10
3	18.06	0.05

MS and HPLC data for *S. mitis*-CSP-2 analogs

Table S-2. MS and HPLC data for *S. mitis*-CSP-2 alanine-screen analogs.

Compound Name	Calc. EM MH_2^{2+}	Obs. EM MH_2^{2+}	Purity (%)
S. mitis-CSP-2 (synthesized)	1077.5808	1077.5829	≥99
S. mitis-CSP-2 (extracted)	1077.5808	1077.5833	≥99
S. mitis-CSP-2-E1A	1048.5781	1048.5754	≥99
S. mitis-CSP-2-I2A	1056.5573	1056.5587	≥99
S. mitis-CSP-2-R3A	1035.0488	1035.0520	≥99
S. mitis-CSP-2-Q4A	1049.0701	1049.0711	≥99
S. mitis-CSP-2-T5A	1062.5755	1062.5787	≥99
S. mitis-CSP-2-H6A	1044.5699	1044.5712	≥99
S. mitis-CSP-2-N7A	1056.0779	1056.0822	≥99
S. mitis-CSP-2-I8A	1056.5573	1056.5610	≥98
S. mitis-CSP-2-F9A	1039.5651	1039.5666	≥99
S. mitis-CSP-2-F10A	1039.5651	1039.5665	≥99
S. mitis-CSP-2-N11A	1056.0779	1056.0797	≥99
S. mitis-CSP-2-F12A	1039.5651	1039.5674	≥99
S. mitis-CSP-2-F13A	1039.5651	1039.5674	≥99
S. mitis-CSP-2-K14A	1049.0519	1049.0558	≥98
S. mitis-CSP-2-R15A	1035.0488	1035.0519	≥99
S. mitis-CSP-2-R16A	1035.0488	1035.0507	≥99

EM = Exact Mass. See the methods above.

Table S-3. MS and HPLC data for *S. mitis*-CSP-2 D-amino acid scan analogs.

Compound Name	Calc. EM MH_2^{2+}	Obs. EM MH_2^{2+}	Purity (%)
S. mitis-CSP-2-e1	1077.5808	1077.5802	≥99
S. mitis-CSP-2-i2	1077.5808	1077.5807	≥99
S. mitis-CSP-2-r3	1077.5808	1077.5835	≥99
S. mitis-CSP-2-q4	1077.5808	1077.5834	≥99
S. mitis-CSP-2-t5	1077.5808	1077.5806	≥99
S. mitis-CSP-2-h6	1077.5808	1077.5793	≥99
S. mitis-CSP-2-n7	1077.5808	1077.5803	≥98
S. mitis-CSP-2-i8	1077.5808	1077.5785	≥96
S. mitis-CSP-2-f9	1077.5808	1077.5787	≥99
S. mitis-CSP-2-f10	1077.5808	1077.5795	≥99
S. mitis-CSP-2-n11	1077.5808	1077.5814	≥98
S. mitis-CSP-2-f12	1077.5808	1077.5800	≥99
S. mitis-CSP-2-f13	1077.5808	1077.5820	≥99
S. mitis-CSP-2-k14	1077.5808	1077.5831	≥99
S. mitis-CSP-2-r15	1077.5808	1077.5832	≥99
S. mitis-CSP-2-r16	1077.5808	1077.5845	≥99

EM = Exact Mass. See the methods above.

Table S-4. MS and HPLC data for *S. mitis*-CSP-2 truncated analogs.

Compound Name	Calc. EM MH_2^{2+}	Obs. EM MH_2^{2+}	Purity (%)
S. mitis-CSP-2-des-E1	1013.0595	1013.0624	≥99
S. mitis-CSP-2-des-E1I2	956.5175	956.5205	≥99
S. mitis-CSP-2-des-E1I2R3	878.4669	878.4683	≥95
S. mitis-CSP-2-des-R16	999.5303	999.5330	≥99
S. mitis-CSP-2-des-R15R16	921.4797	921.4823	≥99
S. mitis-CSP-2-des-K14R15R16	856.9308	856.9336	≥99

EM = Exact Mass. See the methods above.

Table S-5. MS and HPLC data for *S. mitis*-CSP-2-E1A modification analogs.

Compound Name	Calc. EM MH_2^{2+}	Obs. EM MH_2^{2+}	Purity (%)
S. mitis-CSP-2-E1AN7A	1027.0752	1027.0790	≥99
S. mitis-CSP-2-E1AN11A	1027.0752	1027.0784	≥99
S. mitis-CSP-2-E1AF12A	1010.5624	1010.5655	≥99
S. mitis-CSP-2-E1AF13A	1010.5624	1010.5651	≥99
S. mitis-CSP-2-E1Ai8	1048.5781	1048.5808	≥98
S. mitis-CSP-2-E1Af10	1048.5781	1048.5812	≥99
S. mitis-CSP-2-E1An11	1048.5781	1048.5790	≥99
S. mitis-CSP-2-E1Ak14	1048.5781	1048.5788	≥99
S. mitis-CSP-2-E1Ar16	1048.5781	1048.5790	≥99
S. mitis-CSP-2-E1A-des-R16	970.5275	970.5297	≥97
S. mitis-CSP-2-E1AN7Af10	1027.0752	1027.0769	≥98
S. mitis-CSP-2-E1AN7An11	1027.0752	1027.0755	≥99
S. mitis-CSP-2-E1AN7Ak14	1027.0752	1027.0778	≥99
S. mitis-CSP-2-E1AN7Ar16	1027.0752	1027.0767	≥99
S. mitis-CSP-2-E1Af10n11	1048.5781	1048.5782	≥99
S. mitis-CSP-2-E1Af10k14	1048.5781	1048.5780	≥99
S. mitis-CSP-2-E1Af10r16	1048.5781	1048.5780	≥99
S. mitis-CSP-2-E1An11k14	1048.5781	1048.5774	≥99
S. mitis-CSP-2-E1An11r16	1048.5781	1048.5782	≥99
S. mitis-CSP-2-E1Ak14r16	1048.5781	1048.5782	≥99

EM = Exact Mass. See the methods above.

Primary reporter gene assay data

Agonism assays were performed at a 10 µM concentration of synthetic CSP. *S. mitis*-CSP-2 was used as the positive control (100%) while DMSO as the negative control (0%). For all figures, *comX* expression is presented as relative luminescence units (RLU) divided by measured cultural optical density (OD). Percent (%) ComD activation was measured by normalizing the RLU/OD value obtained for each peptide to that of the native *S. mitis*-CSP-2. The results are averages from three replicates and representative of three independent experiments. Error bars represent the standard error of the mean of nine values.

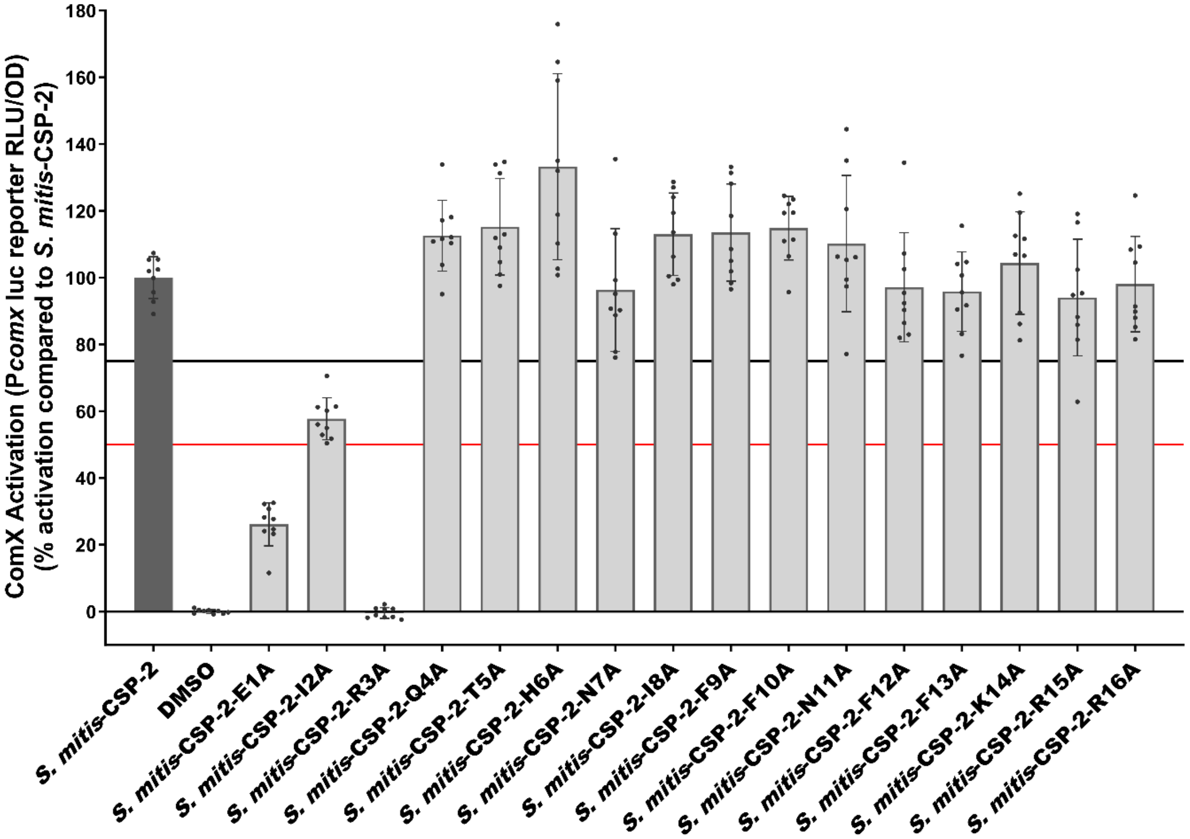

Figure S-1. Primary agonism screening assay data for the *S. mitis*-CSP-2 alanine scan analogs. Peptides that exhibited over 75% activation were further evaluated to determine their EC_{50} values while peptides that exhibited less than 50% activation were evaluated as potential competitive inhibitors.

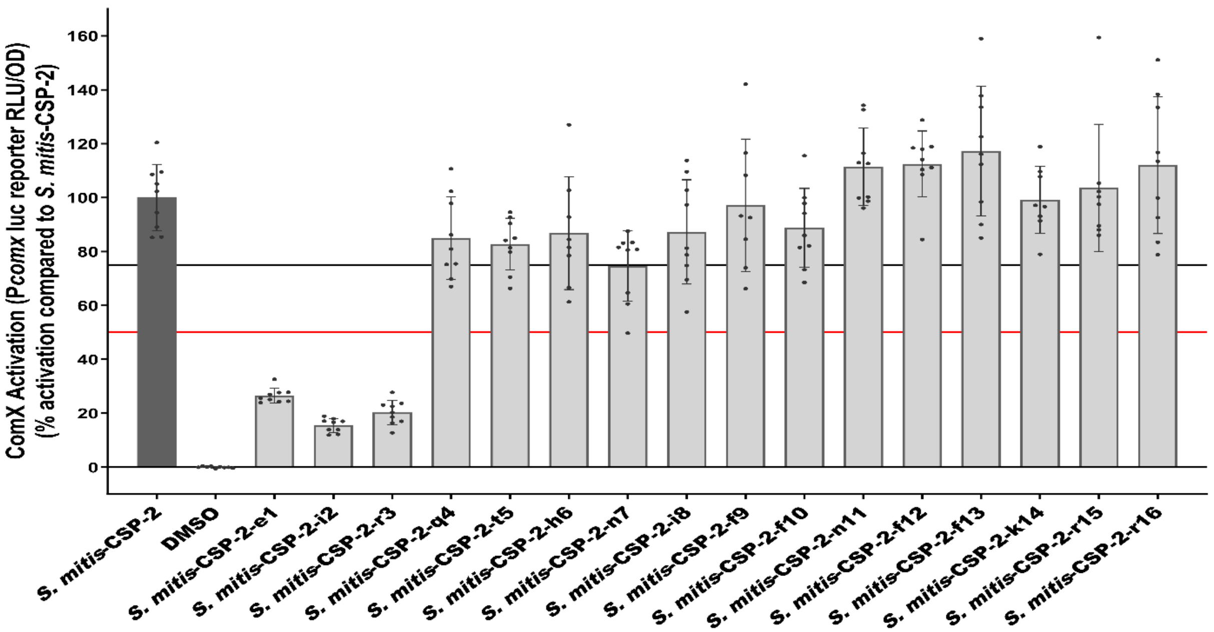

Figure S-2. Primary agonism screening assay data for the *S. mitis*-CSP-2 D-amino acid scan library. Peptides that exhibited over 75% activation were further evaluated to determine their EC_{50} values while peptides that exhibited less than 50% activation were evaluated as potential competitive inhibitors.

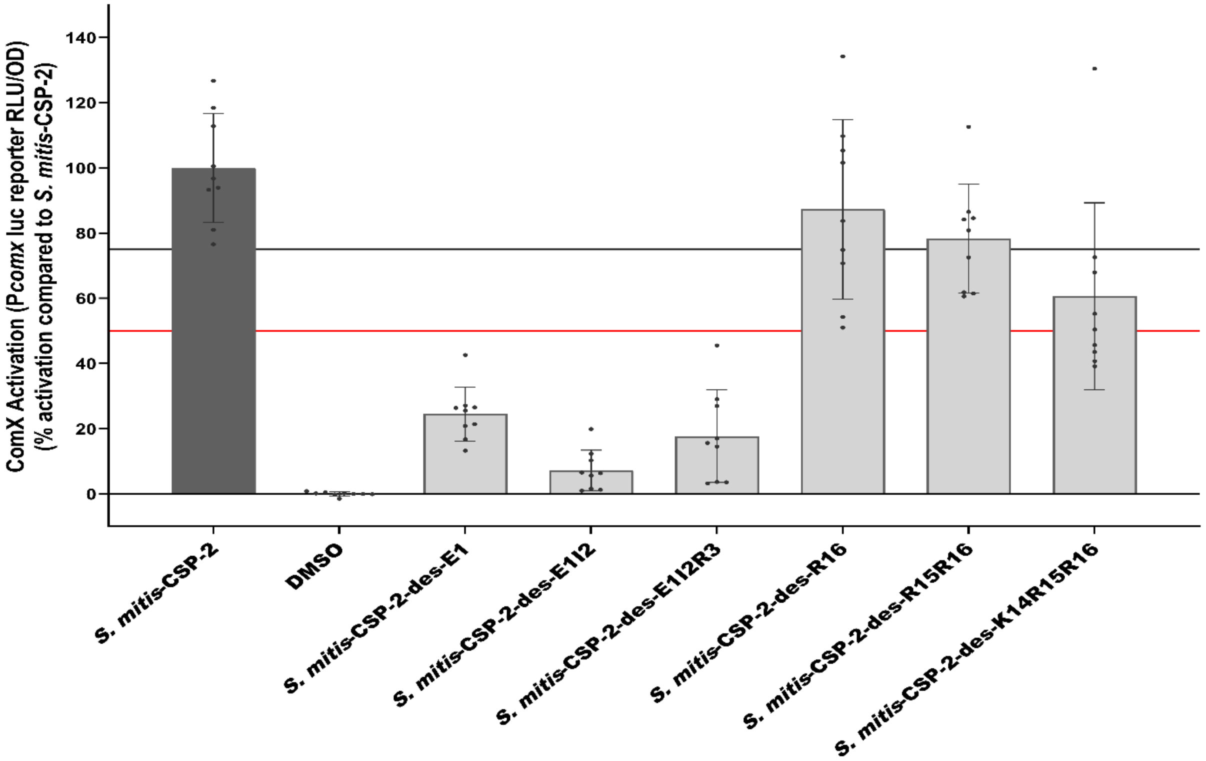

Figure S-3. Primary agonism screening assay data for the *S. mitis*-CSP-2 truncated analogs. Peptides that exhibited over 75% activation were further evaluated to determine their EC_{50} values while peptides that exhibited less than 50% activation were evaluated as potential competitive inhibitors.

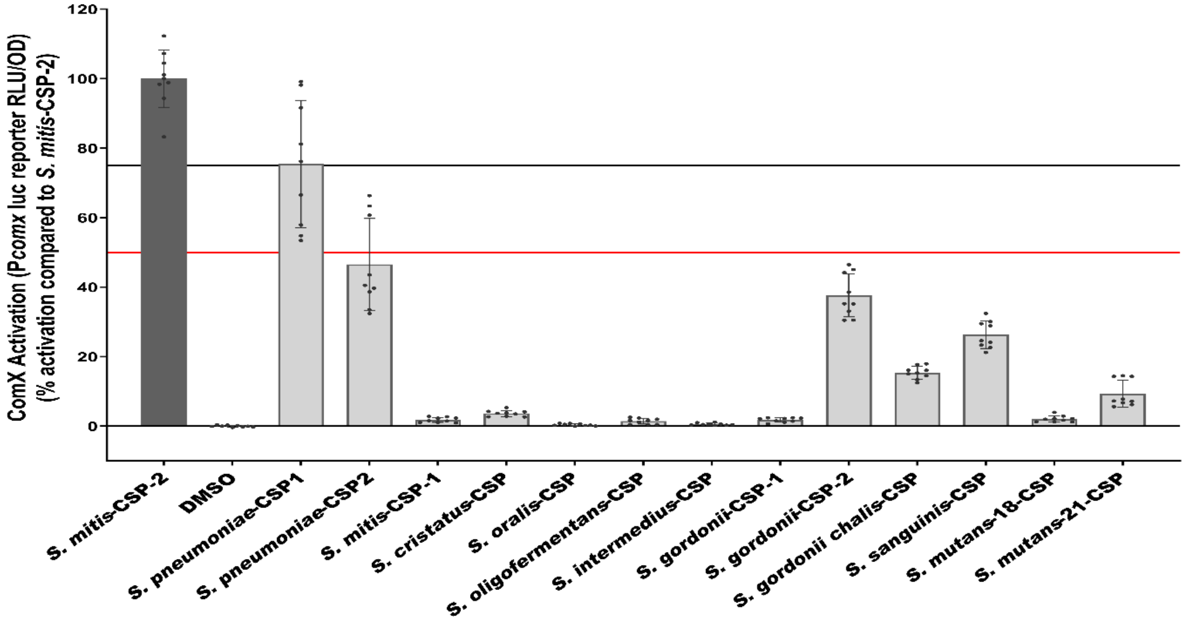

Figure S-4. Primary agonism screening assay data for the synthetic *Streptococci* native CSP pheromones.[1] *S. pneumoniae* CSP1, which exhibited over 75% activation, was further evaluated to determine its EC_{50} value, while peptides that exhibited less than 50% activation were evaluated as potential competitive inhibitors.

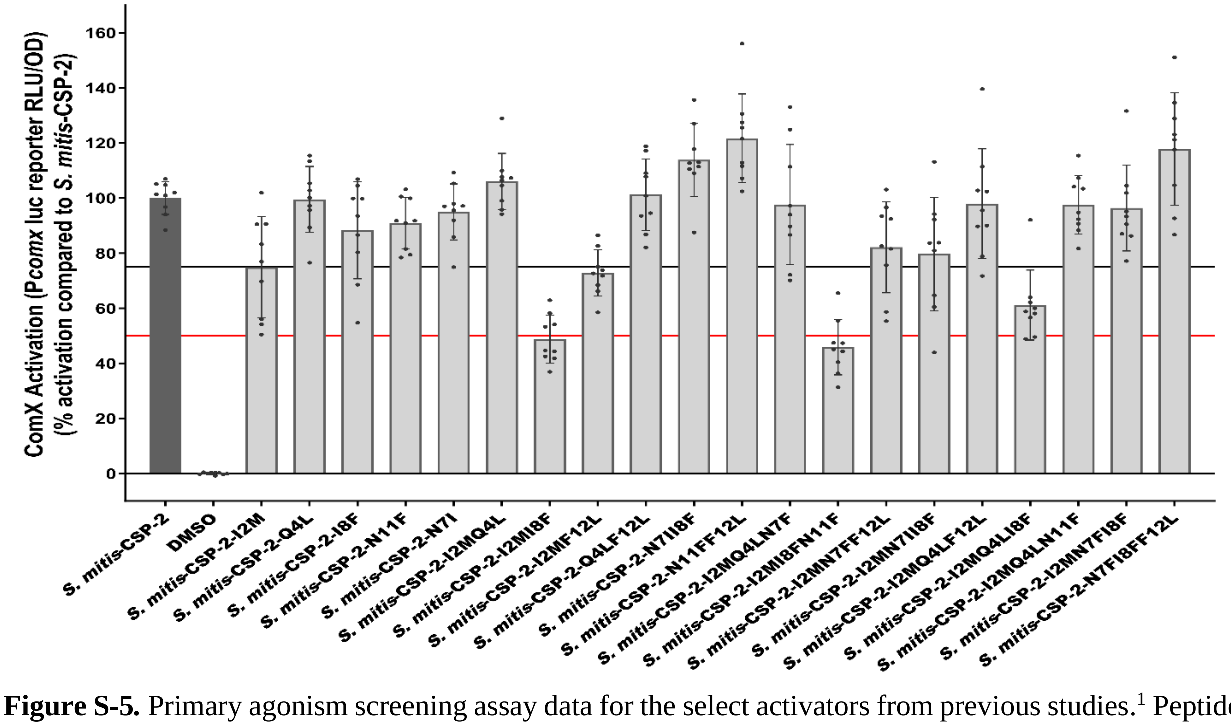

Figure S-5. Primary agonism screening assay data for the select activators from previous studies.[1] Peptides that exhibited over 75% activation were further evaluated to determine their EC_{50} values while peptides that exhibited less than 50% activation were evaluated as potential competitive inhibitors.

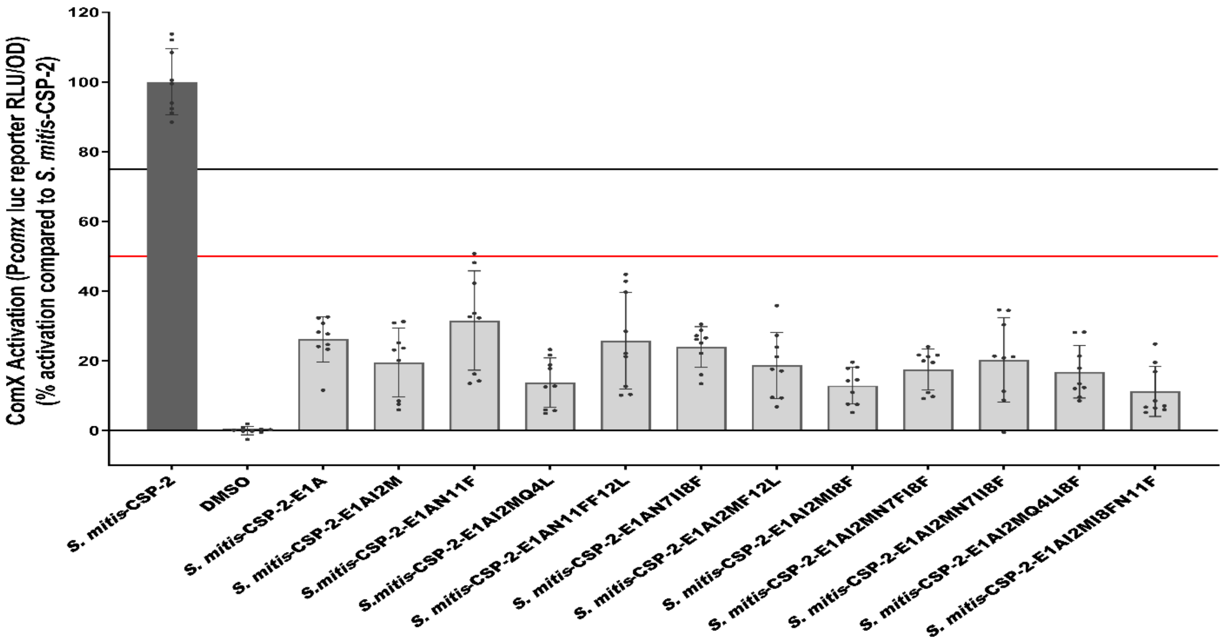

Figure S-6. Primary agonism screening assay data for the *S. mitis*-CSP-2-E1A mutant analogs from previous studies.[1] None of the peptides exhibited activation of the ComD receptor and peptides that exhibited less than 50% activation were evaluated as potential competitive inhibitors.

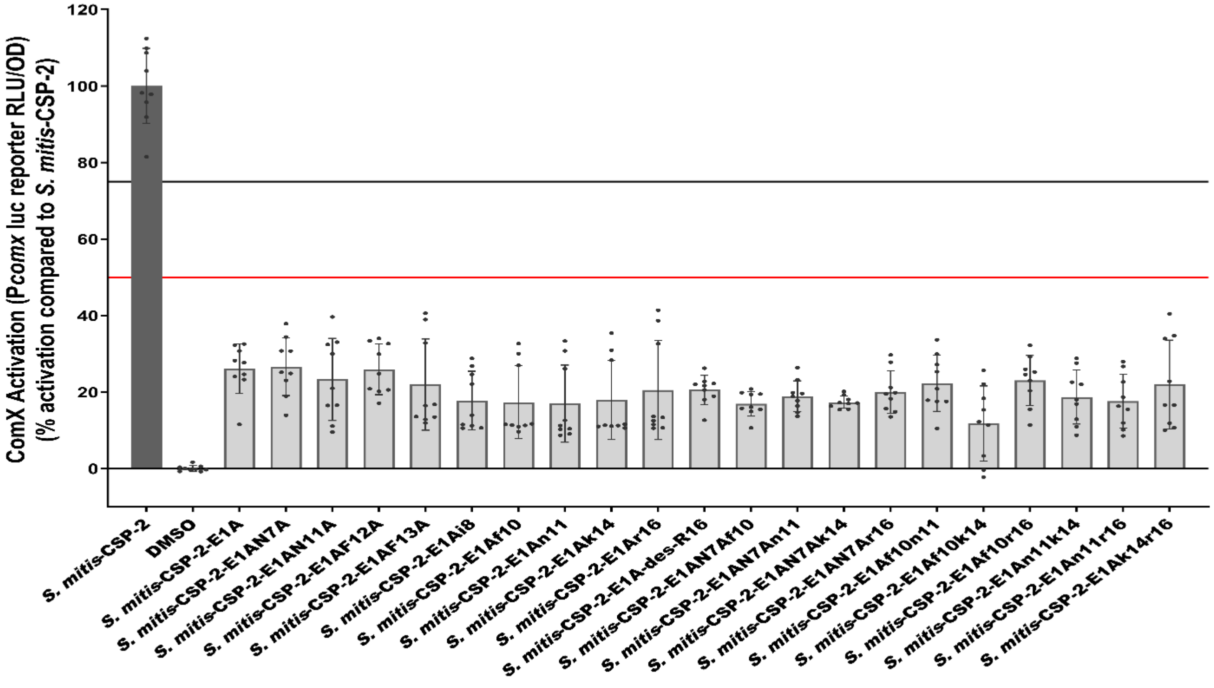

Figure S-7. Primary agonism screening assay data for the *S. mitis*-CSP-2-E1A modification analogs. None of the peptides exhibited activation of the ComD receptor and peptides that exhibited less than 50% activation were evaluated as potential competitive inhibitors.

Antagonism assays were performed at 10 µM concentration of peptides against 1 µM concentration of *S. mitis*-CSP-2. *S. mitis*-CSP-2 (1 µM) was used as the positive control (100%) while DMSO as the negative control (0%). For all figures, *comX* expression is presented as relative luminescence units (RLU) divided by measured cultural optical density (OD). Percent (%) ComD activation was measured by normalizing the RLU/OD value obtained for each peptide to that of the native *S. mitis*-CSP-2. The results are averages from three replicates and representative of three independent experiments. Error bars represent the standard error of the mean of nine values.

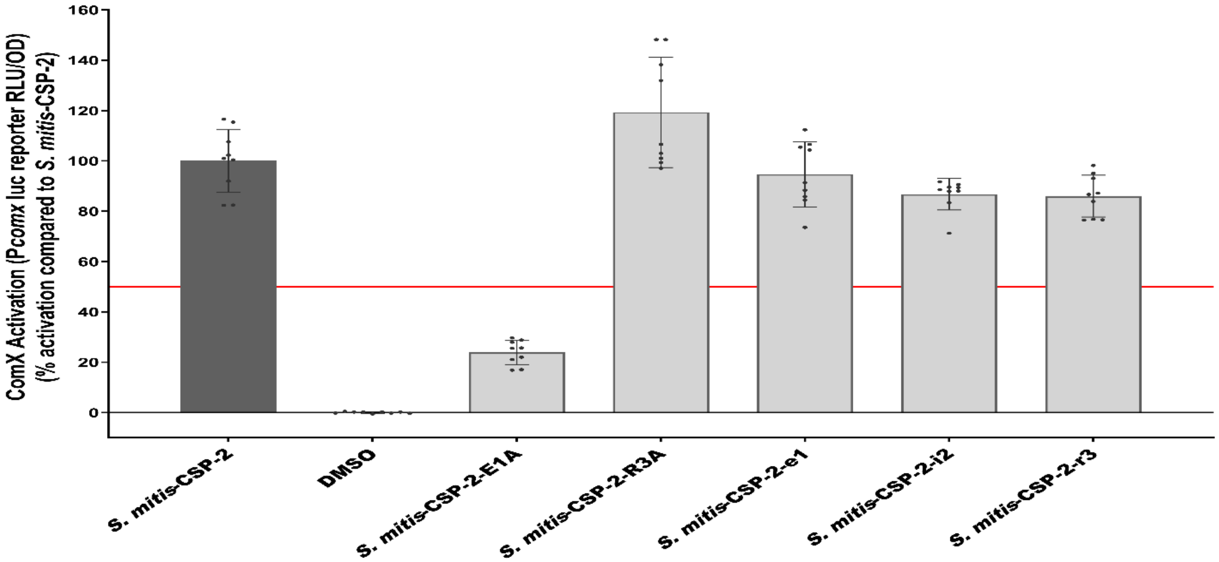

Figure S-8. Primary antagonism screening assay data for the *S. mitis*-CSP-2 alanine scan and D-amino acid scan analogs. *S. mitis*-CSP-2-E1A, which exhibited less than 50% activation, was further evaluated to determine its IC_{50} value.

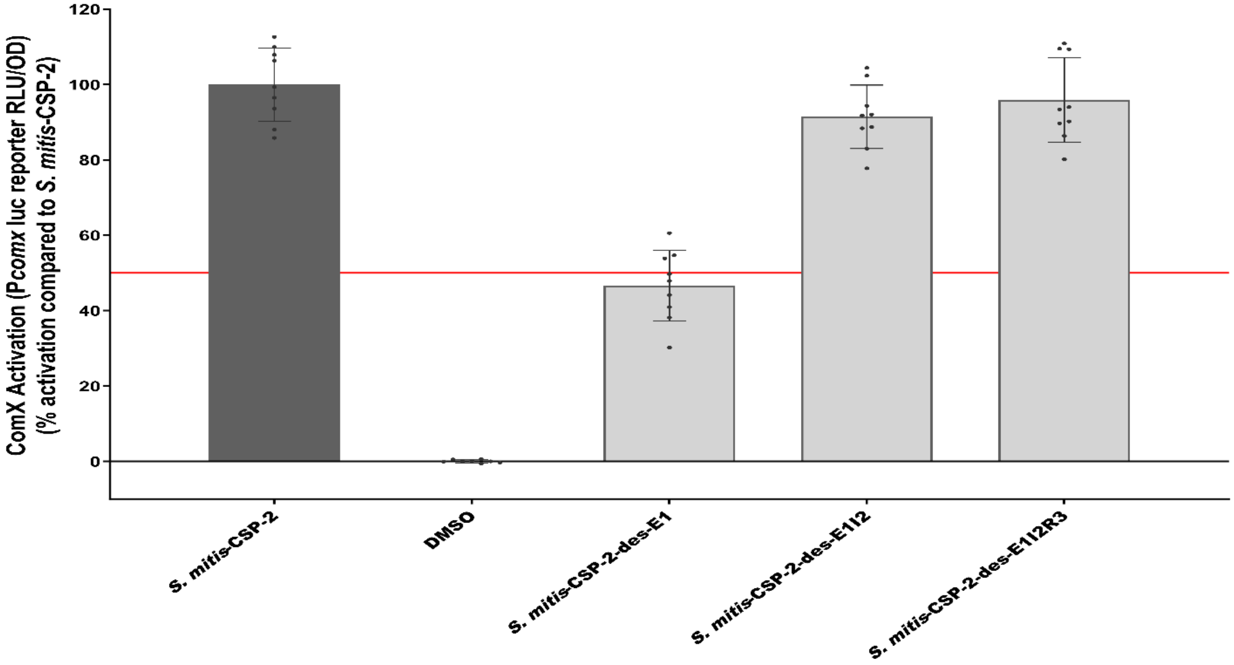

Figure S-9. Primary antagonism screening assay data for the *S. mitis*-CSP-2 truncated analogs. *S. mitis*-CSP-2-des-E1, which exhibited less than 50% activation was further evaluated to determine its IC_{50} value.

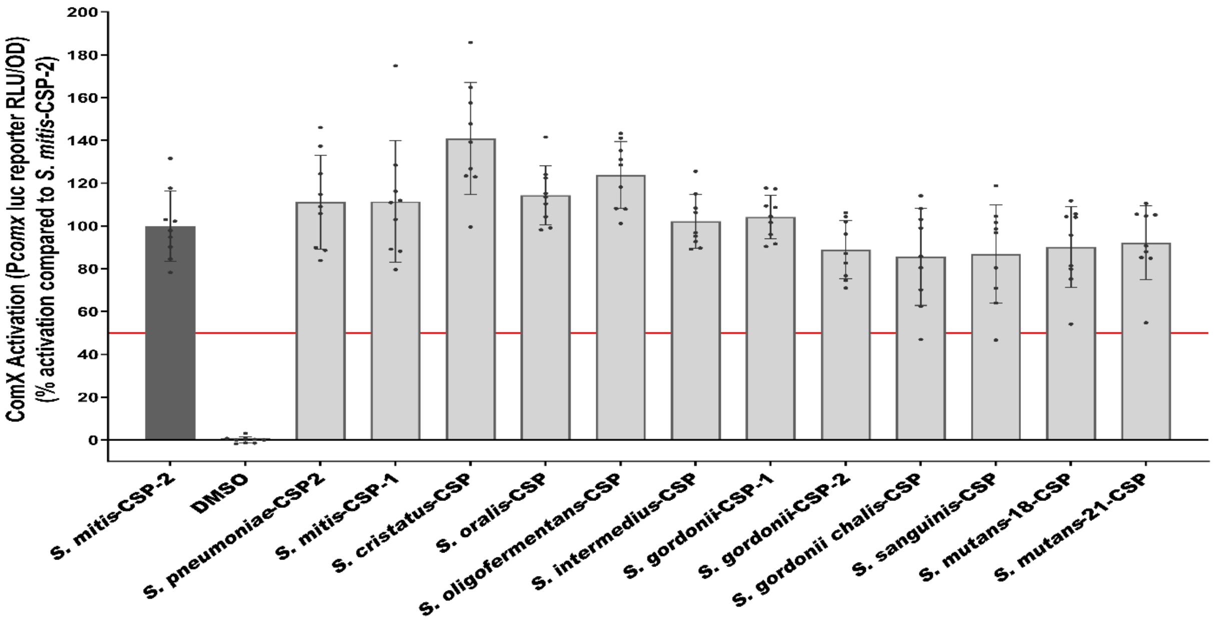

Figure S-10. Primary antagonism screening assay data for the synthetic *Streptococci* native CSP pheromones.[1] None of the peptides exhibited inhibition of the ComD receptor.

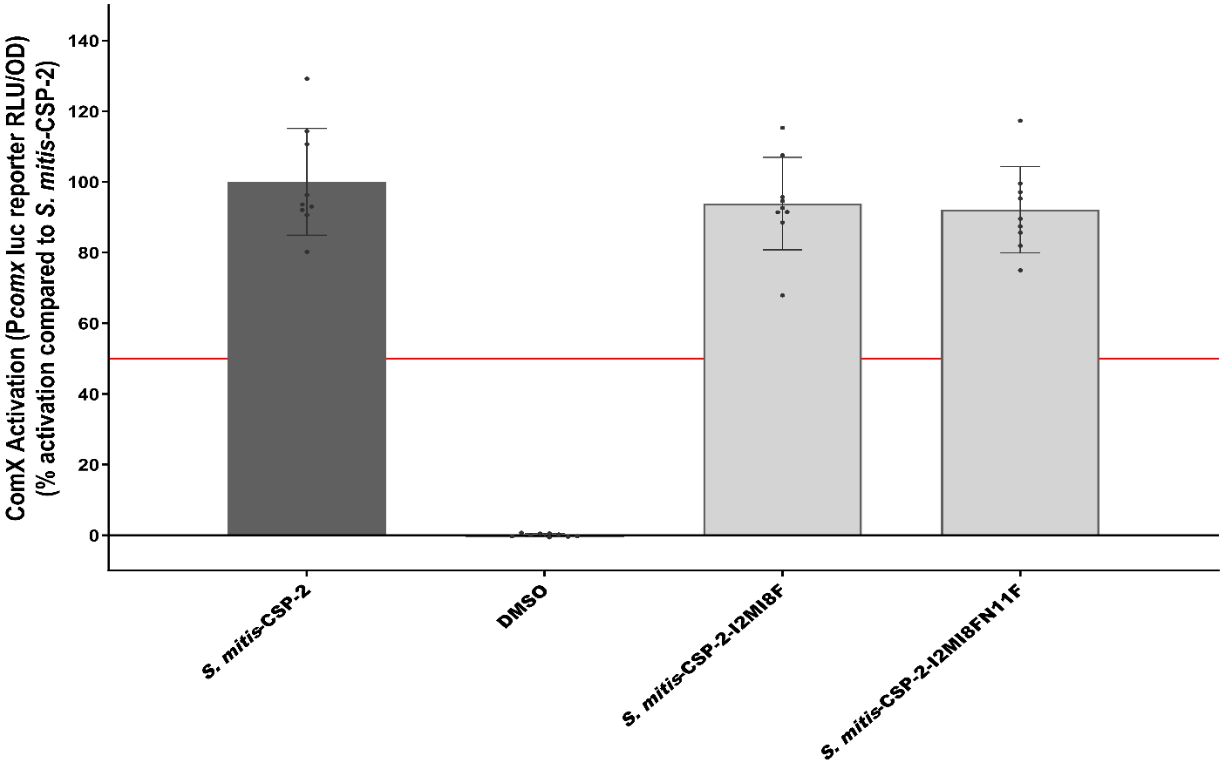

Figure S-11. Primary antagonism screening assay data for the select activators from previous studies.[1] None of the peptides exhibited inhibition of the ComD receptor.

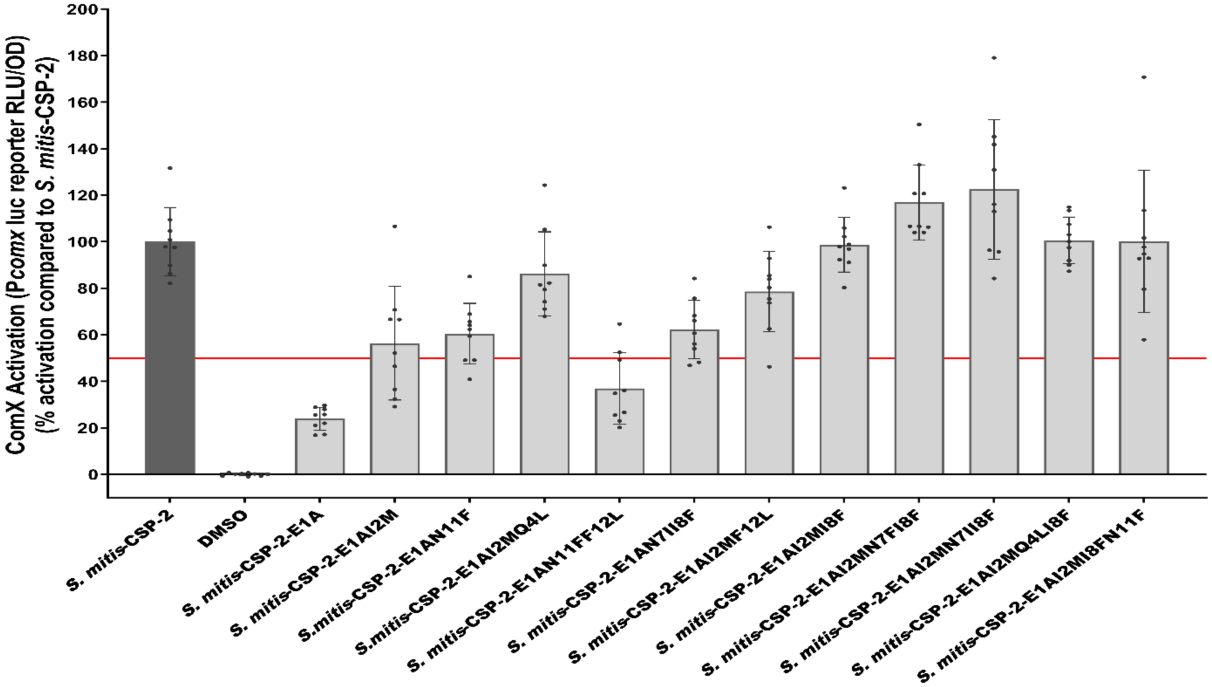

Figure S-12. Primary antagonism screening assay data for the *S. mitis*-CSP-2-E1A mutant analogs from previous studies.[1] Peptides that exhibited less than 50% activation were further evaluated to determine their IC_{50} values.

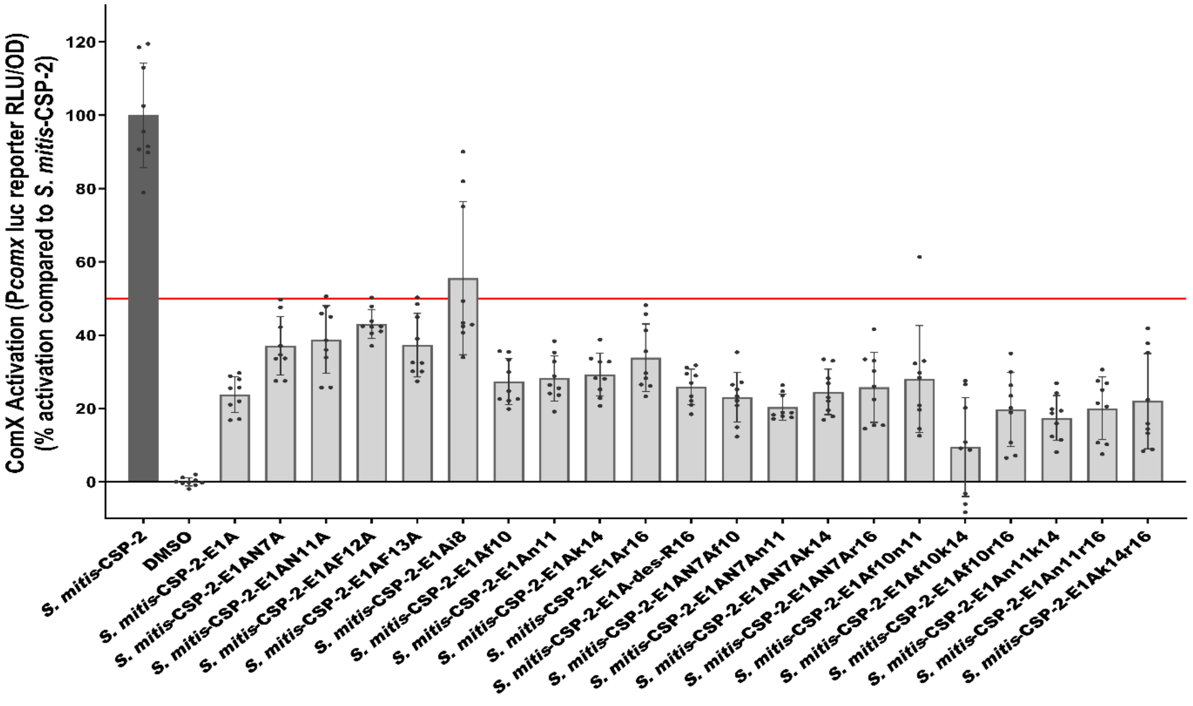

Figure S-13. Primary antagonism screening assay data for the *S. mitis*-CSP-2-E1A modification analogs. Peptides that exhibited less than 50% activation were further evaluated to determine their IC_{50} values.

Agonism and antagonism dose response curves

Synthetic CSP analogs were screened to determine their EC_{50} or IC_{50} values over varying concentrations in the *S. mitis comX* reporter type strain, $ATCC_{49456}$ P_{comX} luc::spc. Each dose response experiment was performed in triplicate on three separate occasions (i.e., experiments (Exp.) #1-3; shown for each peptide below). Error bars indicate standard error of the mean of triplicate values. In each plot, the peptide as well as its EC_{50} or IC_{50} value (in nM) and 95% confidence interval (95% CI) values (in nM), are indicated at top left.

Activation dose response curves

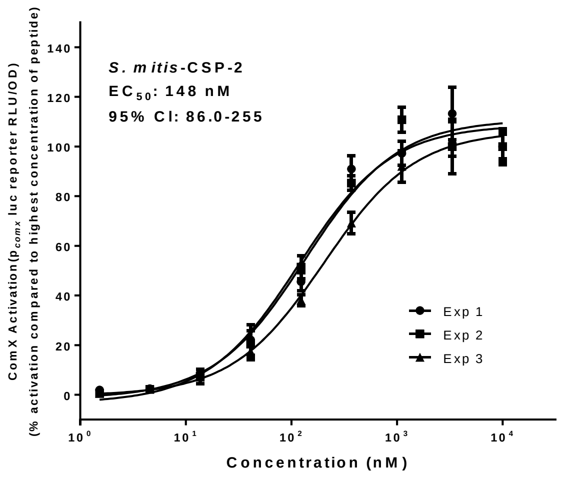

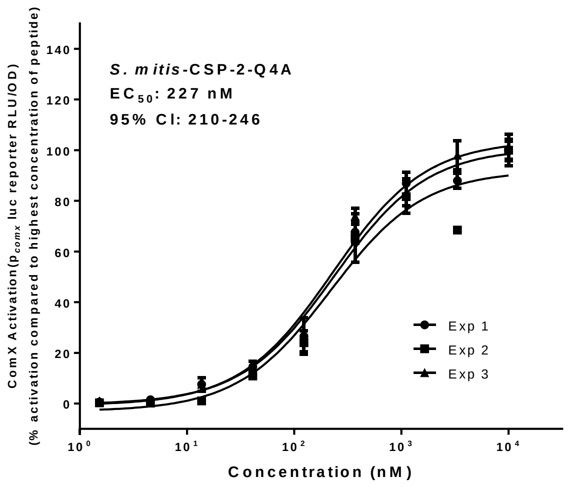

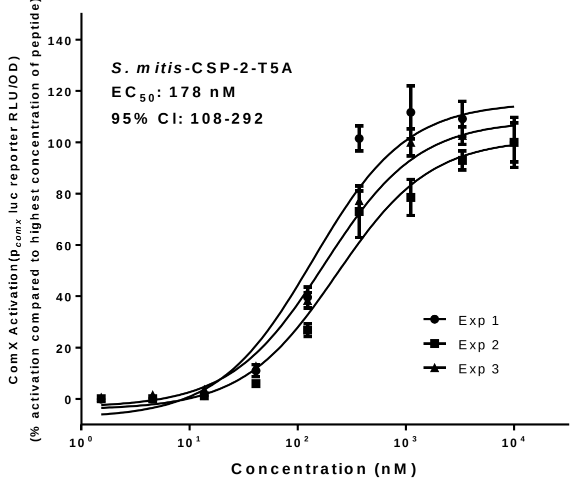

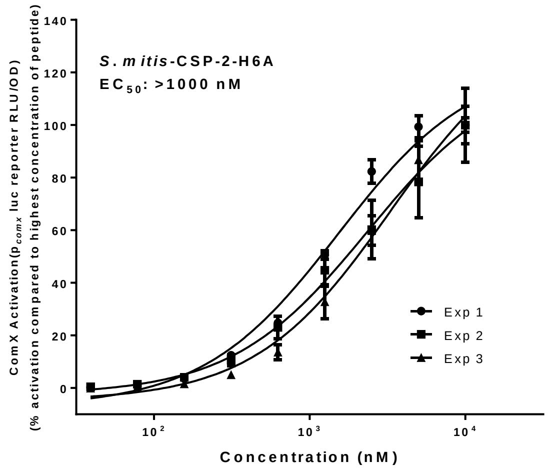

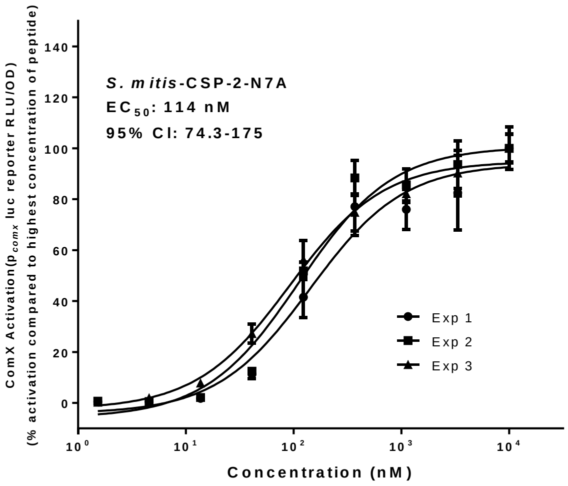
S. mitis-CSP-2-N7A
EC50: 114 nM
95% CI: 74.3-175
Exp 1
Exp 2
Exp 3
ComX Activation(pcomx luc reporter RLU/OD)
(% activation compared to highest concentration of peptide)
Concentration (nM)

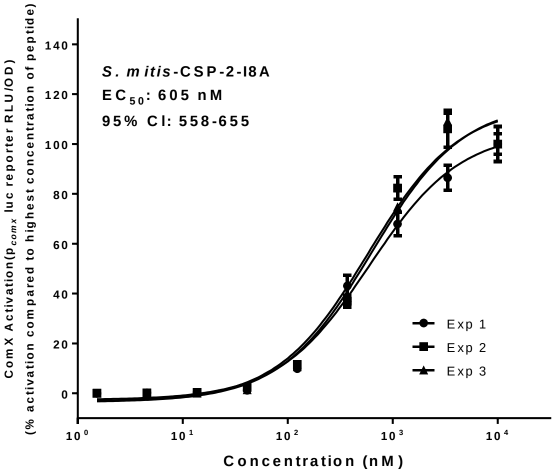
S. mitis-CSP-2-I8A
EC50: 605 nM
95% CI: 558-655
Exp 1
Exp 2
Exp 3
ComX Activation(pcomx luc reporter RLU/OD)
(% activation compared to highest concentration of peptide)
Concentration (nM)

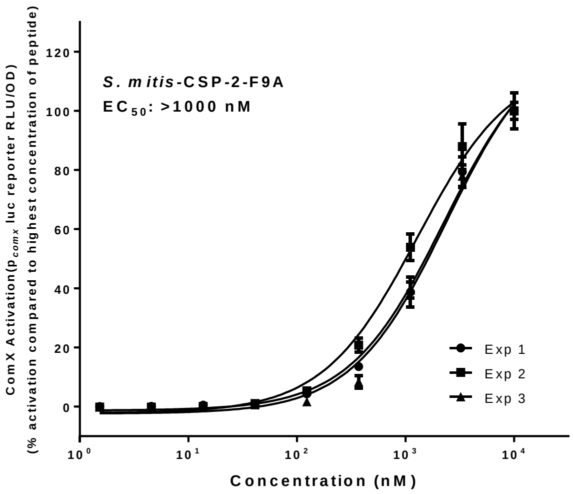
S. mitis-CSP-2-F9A
EC50: >1000 nM
Exp 1
Exp 2
Exp 3
ComX Activation(pcomx luc reporter RLU/OD)
(% activation compared to highest concentration of peptide)
Concentration (nM)

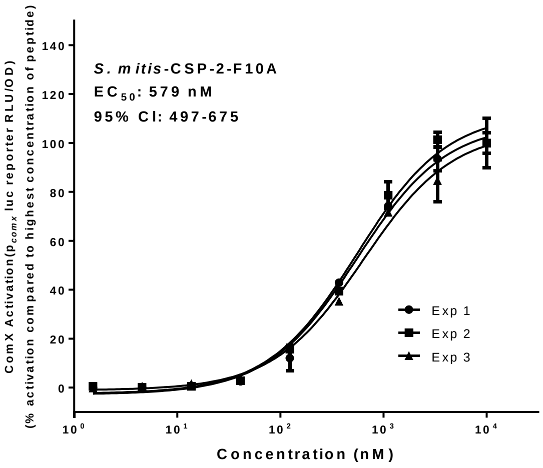
S. mitis-CSP-2-F10A
EC50: 579 nM
95% CI: 497-675
Exp 1
Exp 2
Exp 3
ComX Activation(pcomx luc reporter RLU/OD)
(% activation compared to highest concentration of peptide)
Concentration (nM)

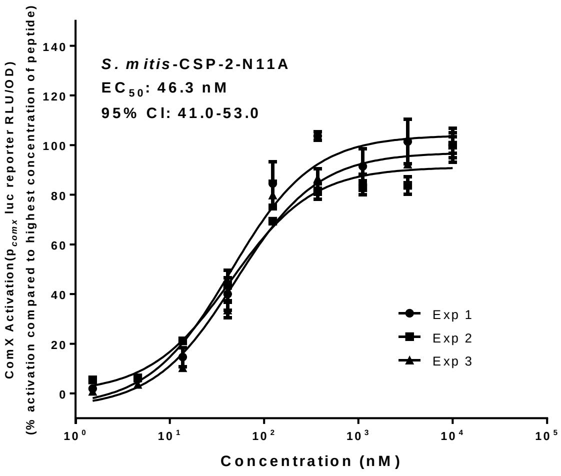
S. mitis-CSP-2-N11A
EC50: 46.3 nM
95% CI: 41.0-53.0
Exp 1
Exp 2
Exp 3
ComX Activation(pcomx luc reporter RLU/OD)
(% activation compared to highest concentration of peptide)
Concentration (nM)

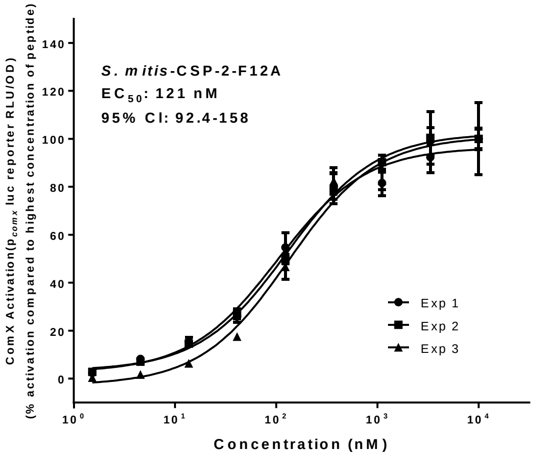
S. mitis-CSP-2-F12A
EC50: 121 nM
95% CI: 92.4-158
Exp 1
Exp 2
Exp 3
ComX Activation(pcomx luc reporter RLU/OD)
(% activation compared to highest concentration of peptide)
Concentration (nM)

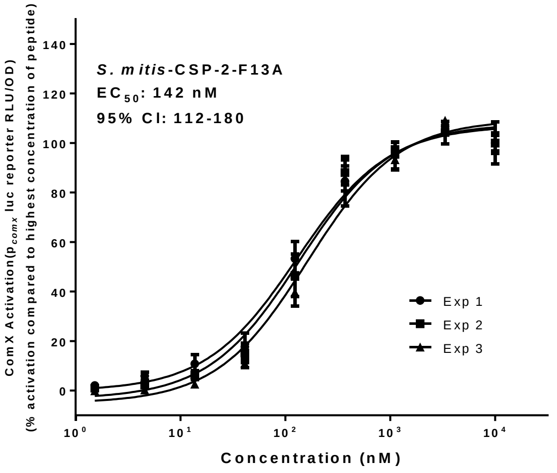
S. mitis-CSP-2-F13A
EC_{50}: 142 nM
95% CI: 112-180
Exp 1
Exp 2
Exp 3
ComX Activation(p_{comx} luc reporter RLU/OD)
(% activation compared to highest concentration of peptide)
Concentration (nM)

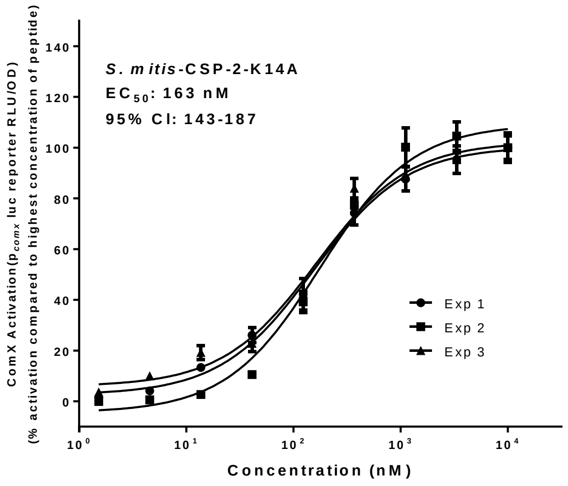
S. mitis-CSP-2-K14A
EC_{50}: 163 nM
95% CI: 143-187
Exp 1
Exp 2
Exp 3
ComX Activation(p_{comx} luc reporter RLU/OD)
(% activation compared to highest concentration of peptide)
Concentration (nM)

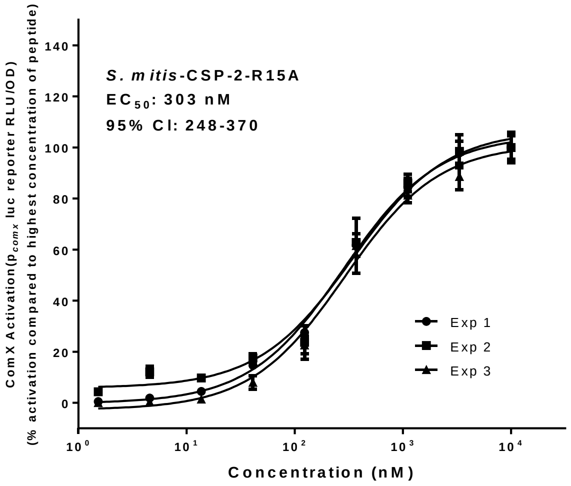
S. mitis-CSP-2-R15A
EC_{50}: 303 nM
95% CI: 248-370
Exp 1
Exp 2
Exp 3
ComX Activation(p_{comx} luc reporter RLU/OD)
(% activation compared to highest concentration of peptide)
Concentration (nM)

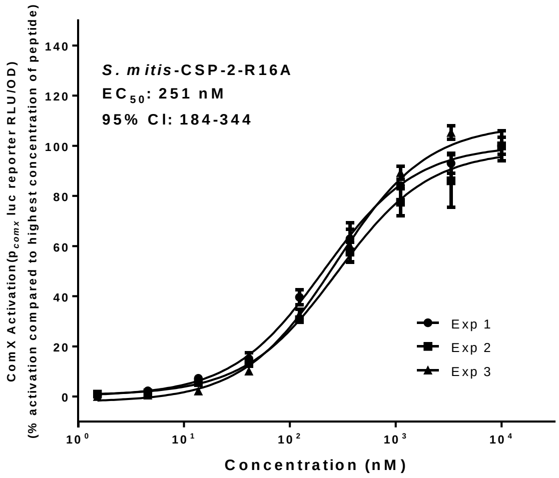
S. mitis-CSP-2-R16A
EC_{50}: 251 nM
95% CI: 184-344
Exp 1
Exp 2
Exp 3
ComX Activation(p_{comx} luc reporter RLU/OD)
(% activation compared to highest concentration of peptide)
Concentration (nM)

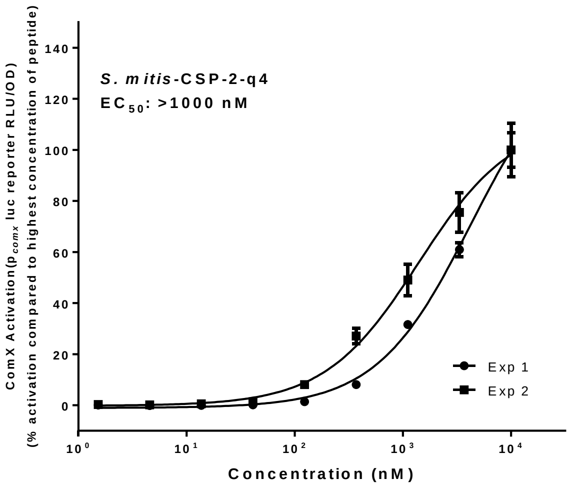
S. mitis-CSP-2-q4
EC_{50}: >1000 nM
Exp 1
Exp 2
ComX Activation(p_{comx} luc reporter RLU/OD)
(% activation compared to highest concentration of peptide)
Concentration (nM)

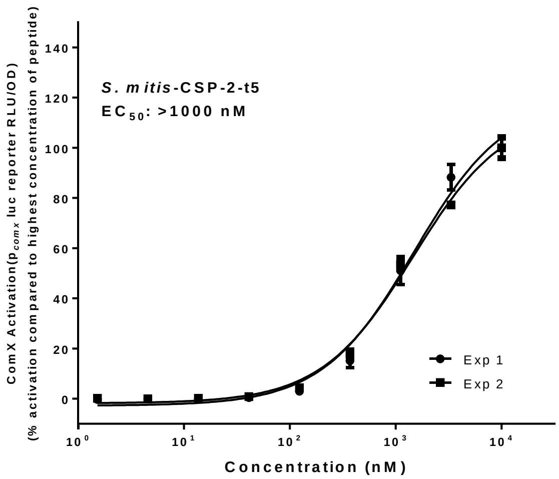
S. mitis-CSP-2-t5
EC_{50}: >1000 nM
Exp 1
Exp 2
ComX Activation(p_{comx} luc reporter RLU/OD)
(% activation compared to highest concentration of peptide)
Concentration (nM)

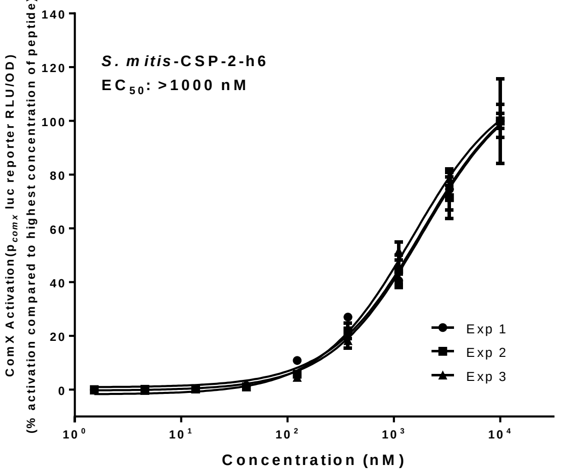
S. mitis-CSP-2-h6
EC50: >1000 nM
ComX Activation(pcomx luc reporter RLU/OD)
(% activation compared to highest concentration of peptide)
Concentration (nM)
Exp 1
Exp 2
Exp 3

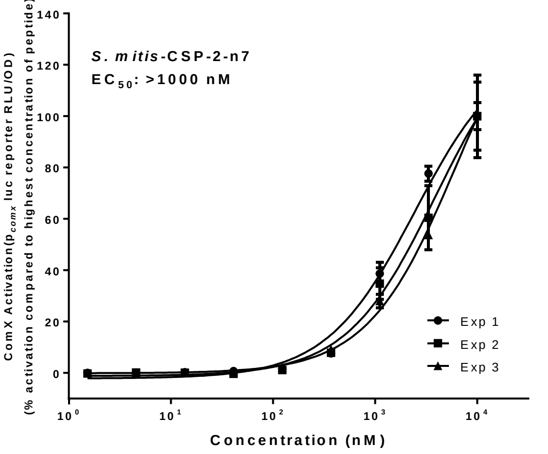
S. mitis-CSP-2-n7
EC50: >1000 nM
ComX Activation(pcomx luc reporter RLU/OD)
(% activation compared to highest concentration of peptide)
Concentration (nM)
Exp 1
Exp 2
Exp 3

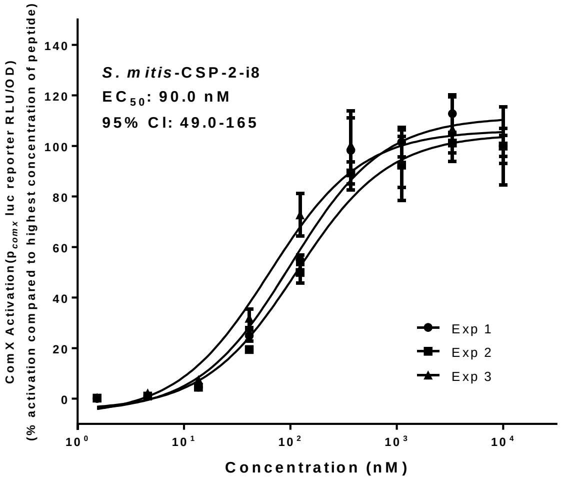
S. mitis-CSP-2-i8
EC50: 90.0 nM
95% CI: 49.0-165
ComX Activation(pcomx luc reporter RLU/OD)
(% activation compared to highest concentration of peptide)
Concentration (nM)
Exp 1
Exp 2
Exp 3

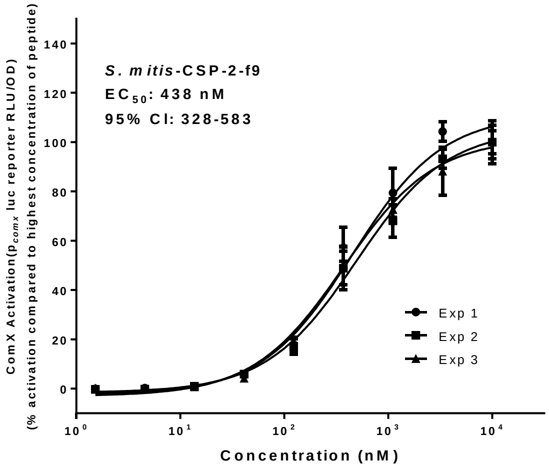
S. mitis-CSP-2-f9
EC50: 438 nM
95% CI: 328-583
ComX Activation(pcomx luc reporter RLU/OD)
(% activation compared to highest concentration of peptide)
Concentration (nM)
Exp 1
Exp 2
Exp 3

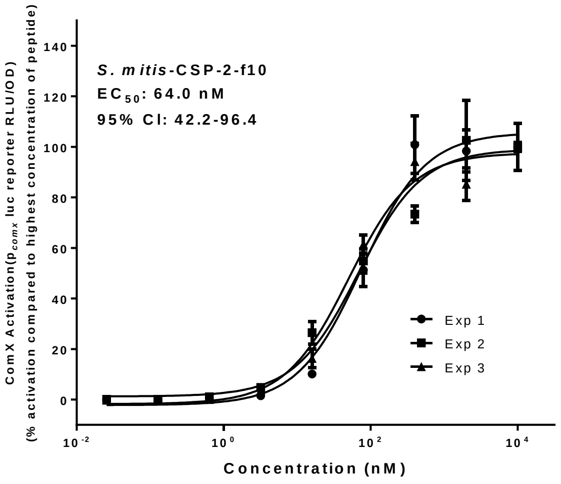
S. mitis-CSP-2-f10
EC50: 64.0 nM
95% CI: 42.2-96.4
ComX Activation(pcomx luc reporter RLU/OD)
(% activation compared to highest concentration of peptide)
Concentration (nM)
Exp 1
Exp 2
Exp 3

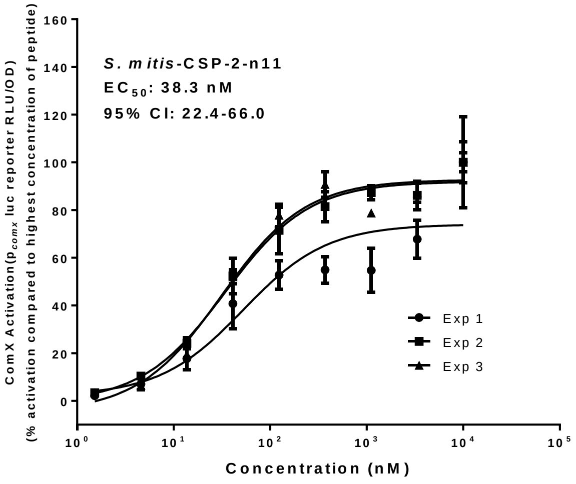
S. mitis-CSP-2-n11
EC50: 38.3 nM
95% CI: 22.4-66.0
ComX Activation(pcomx luc reporter RLU/OD)
(% activation compared to highest concentration of peptide)
Concentration (nM)
Exp 1
Exp 2
Exp 3

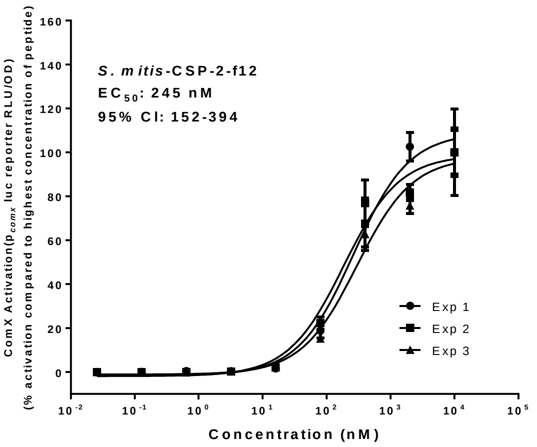
S. mitis-CSP-2-f12
EC50: 245 nM
95% CI: 152-394
Exp 1
Exp 2
Exp 3
ComX Activation(pcomx luc reporter RLU/OD)
(% activation compared to highest concentration of peptide)
Concentration (nM)

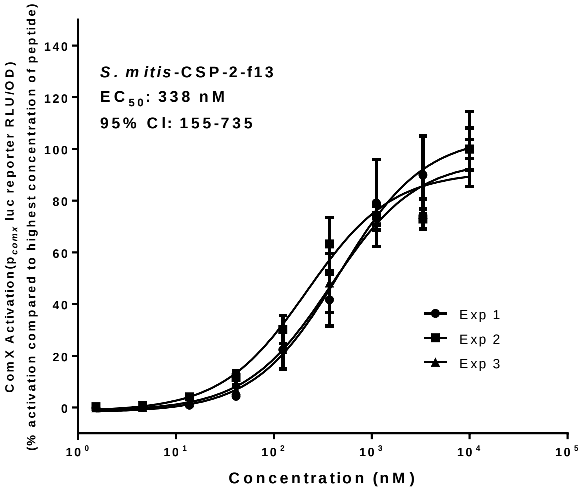
S. mitis-CSP-2-f13
EC50: 338 nM
95% CI: 155-735
Exp 1
Exp 2
Exp 3
ComX Activation(pcomx luc reporter RLU/OD)
(% activation compared to highest concentration of peptide)
Concentration (nM)

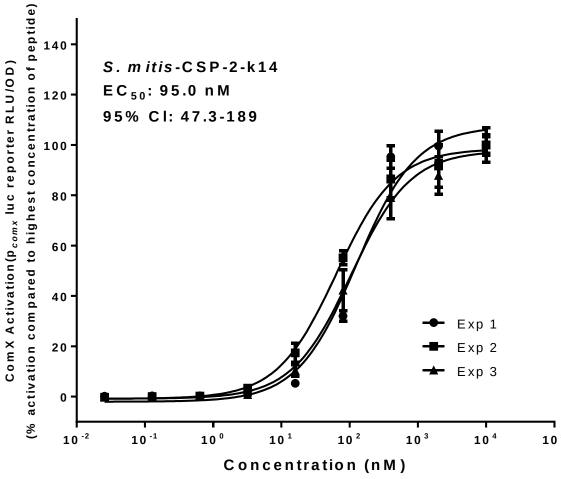
S. mitis-CSP-2-k14
EC50: 95.0 nM
95% CI: 47.3-189
Exp 1
Exp 2
Exp 3
ComX Activation(pcomx luc reporter RLU/OD)
(% activation compared to highest concentration of peptide)
Concentration (nM)

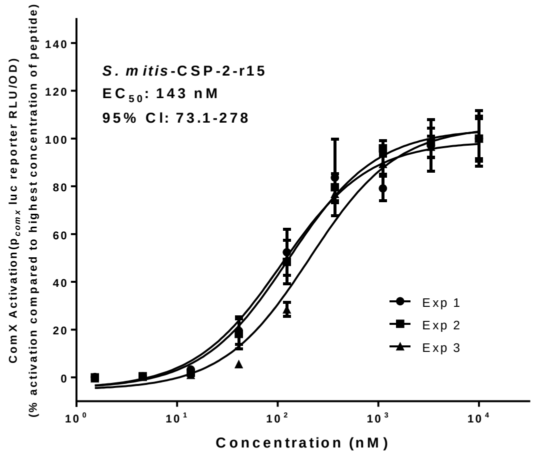
S. mitis-CSP-2-r15
EC50: 143 nM
95% CI: 73.1-278
Exp 1
Exp 2
Exp 3
ComX Activation(pcomx luc reporter RLU/OD)
(% activation compared to highest concentration of peptide)
Concentration (nM)

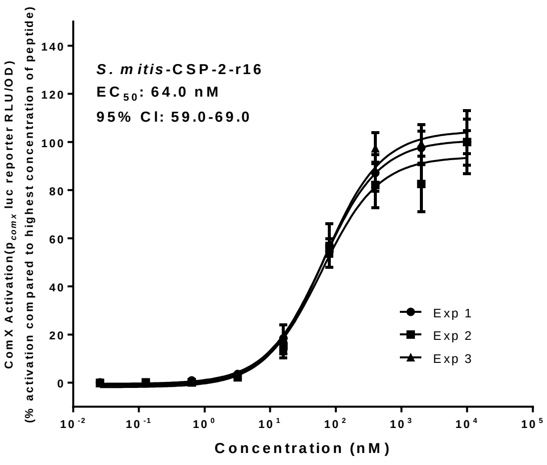
S. mitis-CSP-2-r16
EC50: 64.0 nM
95% CI: 59.0-69.0
Exp 1
Exp 2
Exp 3
ComX Activation(pcomx luc reporter RLU/OD)
(% activation compared to highest concentration of peptide)
Concentration (nM)

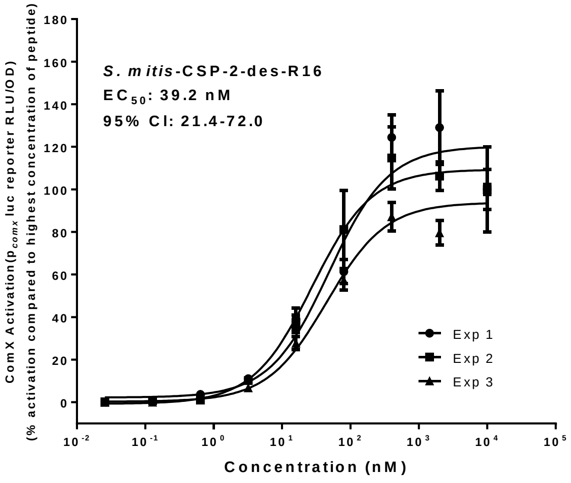
S. mitis-CSP-2-des-R16
EC50: 39.2 nM
95% CI: 21.4-72.0
Exp 1
Exp 2
Exp 3
ComX Activation(pcomx luc reporter RLU/OD)
(% activation compared to highest concentration of peptide)
Concentration (nM)

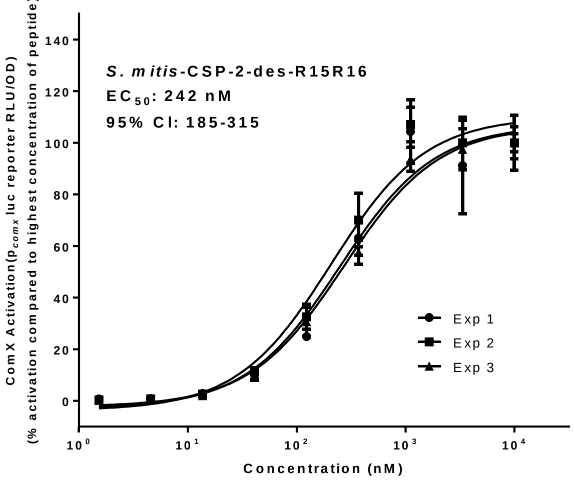
S. mitis-CSP-2-des-R15R16
EC_{50}: 242 nM
95% CI: 185-315
Exp 1
Exp 2
Exp 3
ComX Activation(p_{comx} luc reporter RLU/OD)
(% activation compared to highest concentration of peptide)
Concentration (nM)

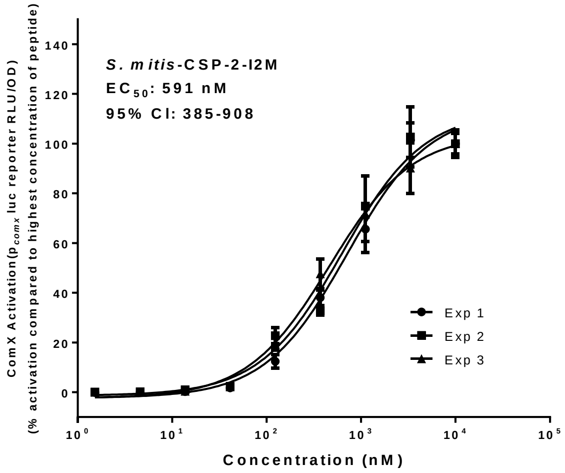
S. mitis-CSP-2-I2M
EC_{50}: 591 nM
95% CI: 385-908
Exp 1
Exp 2
Exp 3
ComX Activation(p_{comx} luc reporter RLU/OD)
(% activation compared to highest concentration of peptide)
Concentration (nM)

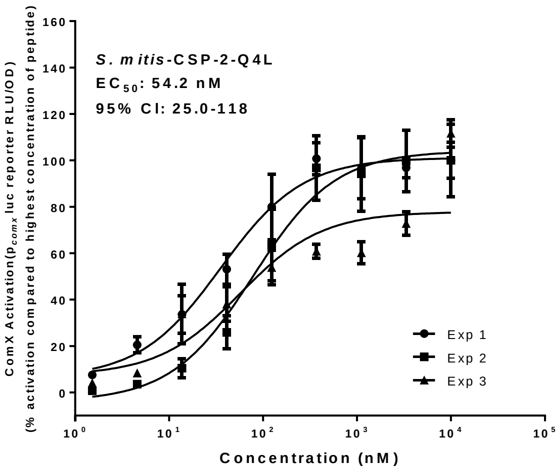
S. mitis-CSP-2-Q4L
EC_{50}: 54.2 nM
95% CI: 25.0-118
Exp 1
Exp 2
Exp 3
ComX Activation(p_{comx} luc reporter RLU/OD)
(% activation compared to highest concentration of peptide)
Concentration (nM)

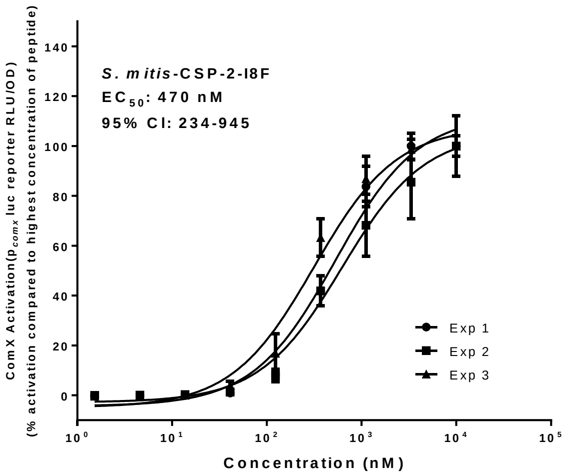
S. mitis-CSP-2-I8F
EC_{50}: 470 nM
95% CI: 234-945
Exp 1
Exp 2
Exp 3
ComX Activation(p_{comx} luc reporter RLU/OD)
(% activation compared to highest concentration of peptide)
Concentration (nM)

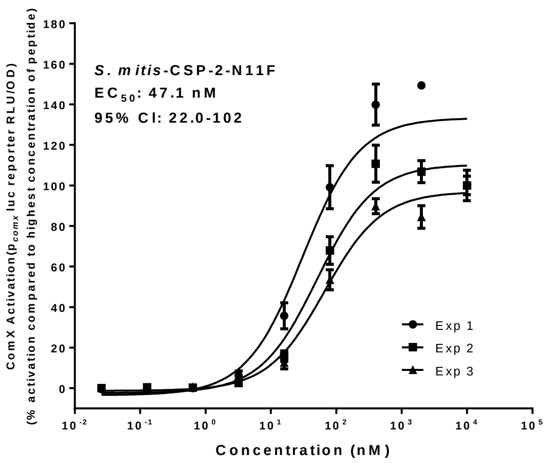
S. mitis-CSP-2-N11F
EC_{50}: 47.1 nM
95% CI: 22.0-102
Exp 1
Exp 2
Exp 3
ComX Activation(p_{comx} luc reporter RLU/OD)
(% activation compared to highest concentration of peptide)
Concentration (nM)

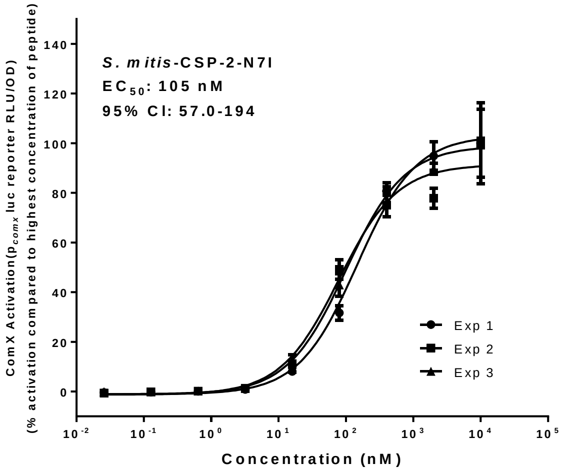
S. mitis-CSP-2-N7I
EC_{50}: 105 nM
95% CI: 57.0-194
Exp 1
Exp 2
Exp 3
ComX Activation(p_{comx} luc reporter RLU/OD)
(% activation compared to highest concentration of peptide)
Concentration (nM)

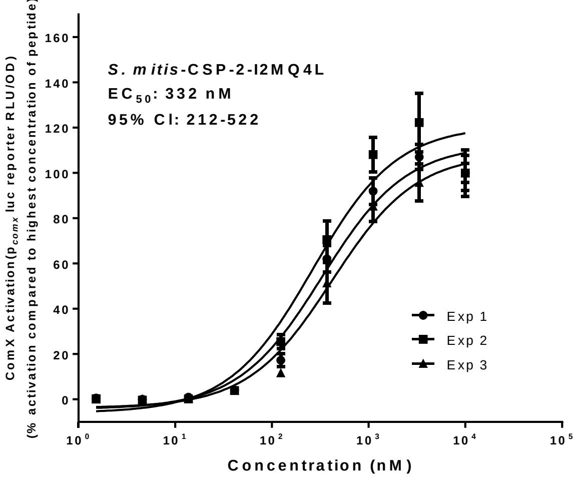
S. mitis-CSP-2-I2MQ4L
EC50: 332 nM
95% CI: 212-522
Exp 1
Exp 2
Exp 3
ComX Activation(pcomx luc reporter RLU/OD)
(% activation compared to highest concentration of peptide)
Concentration (nM)

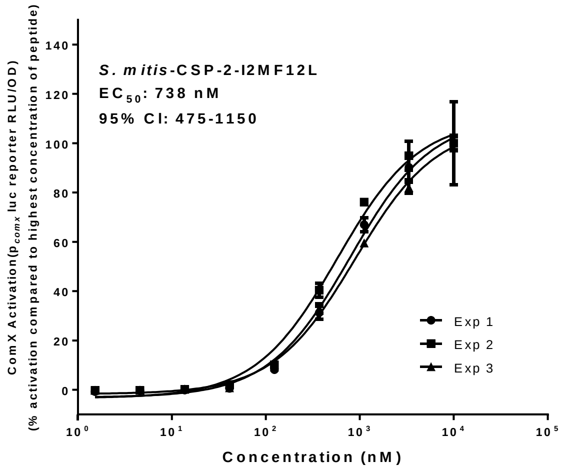
S. mitis-CSP-2-I2MF12L
EC50: 738 nM
95% CI: 475-1150
Exp 1
Exp 2
Exp 3
ComX Activation(pcomx luc reporter RLU/OD)
(% activation compared to highest concentration of peptide)
Concentration (nM)

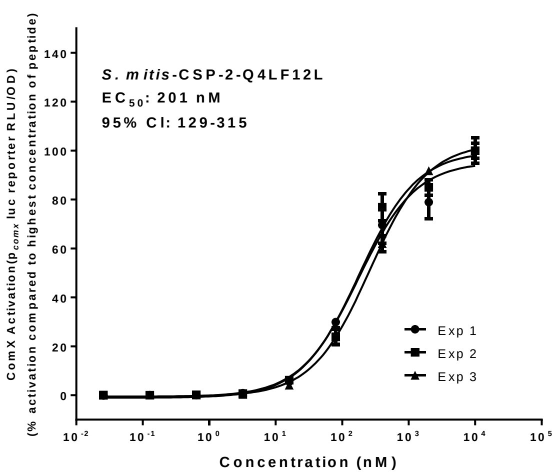
S. mitis-CSP-2-Q4LF12L
EC50: 201 nM
95% CI: 129-315
Exp 1
Exp 2
Exp 3
ComX Activation(pcomx luc reporter RLU/OD)
(% activation compared to highest concentration of peptide)
Concentration (nM)

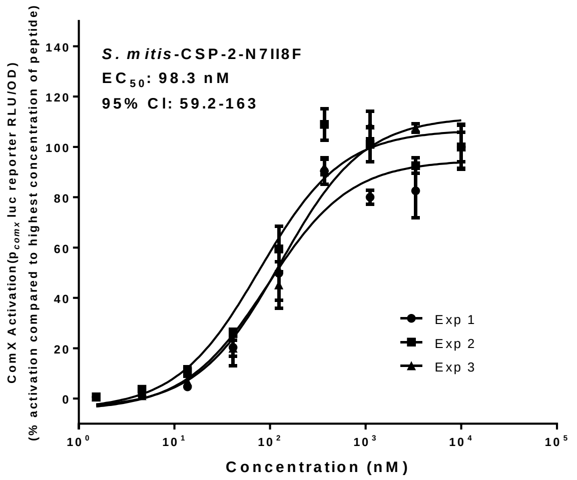
S. mitis-CSP-2-N7I8F
EC50: 98.3 nM
95% CI: 59.2-163
Exp 1
Exp 2
Exp 3
ComX Activation(pcomx luc reporter RLU/OD)
(% activation compared to highest concentration of peptide)
Concentration (nM)

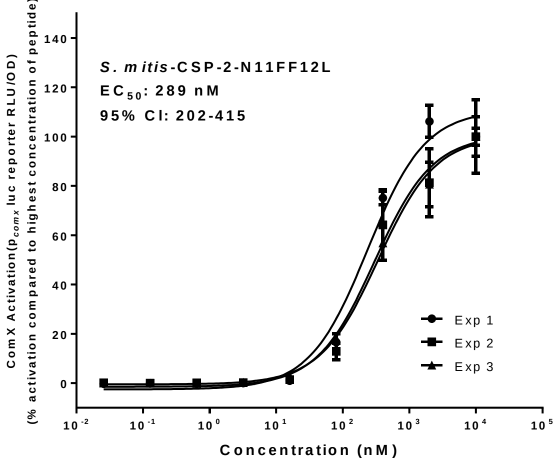
S. mitis-CSP-2-N11FF12L
EC50: 289 nM
95% CI: 202-415
Exp 1
Exp 2
Exp 3
ComX Activation(pcomx luc reporter RLU/OD)
(% activation compared to highest concentration of peptide)
Concentration (nM)

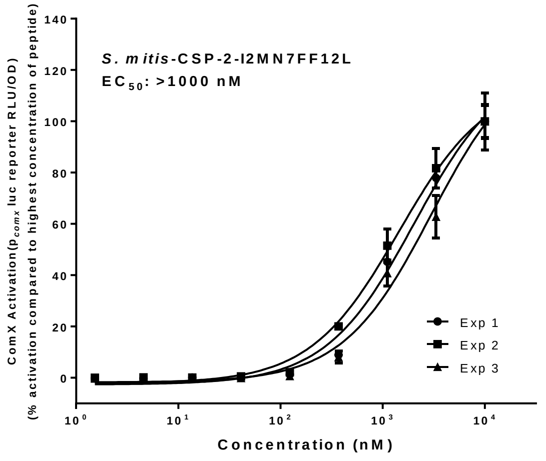
S. mitis-CSP-2-I2MN7FF12L
EC50: >1000 nM
Exp 1
Exp 2
Exp 3
ComX Activation(pcomx luc reporter RLU/OD)
(% activation compared to highest concentration of peptide)
Concentration (nM)

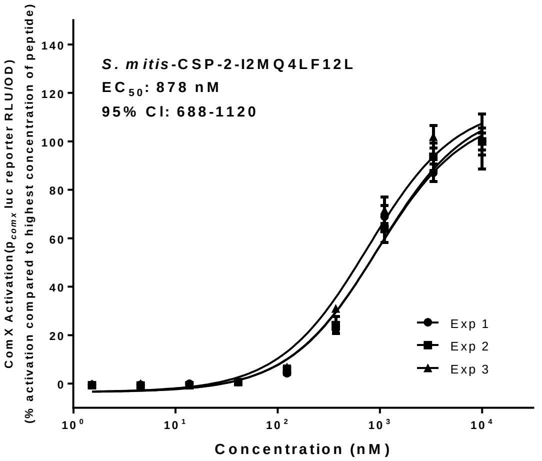

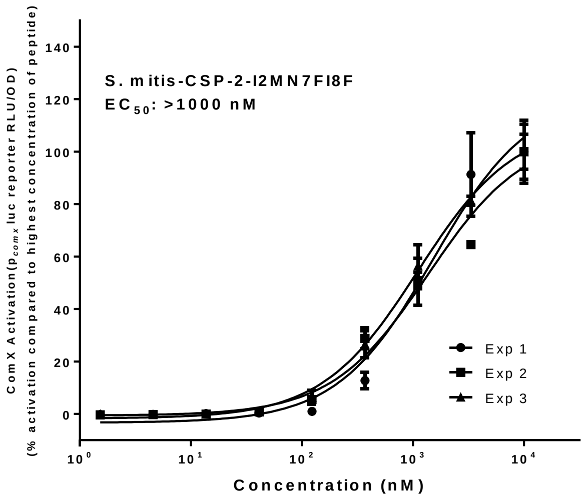

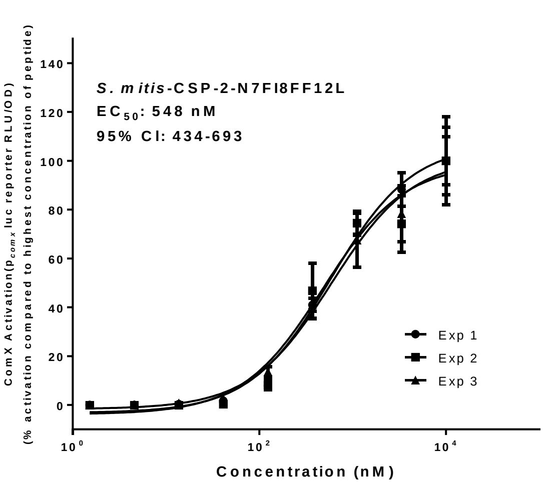

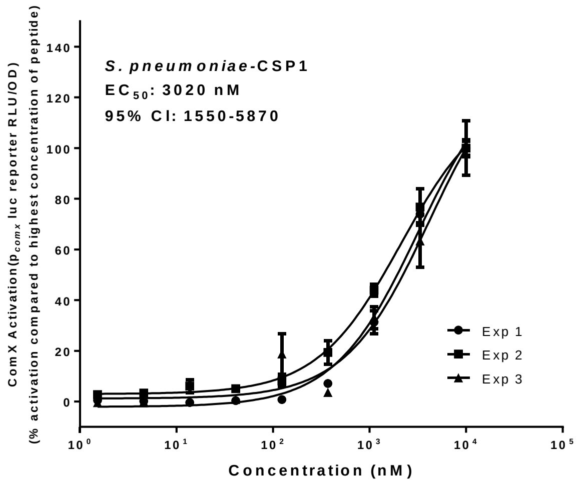

Inhibition dose response curves

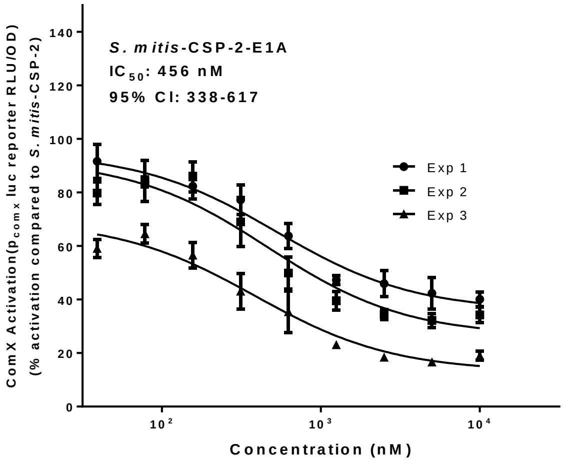

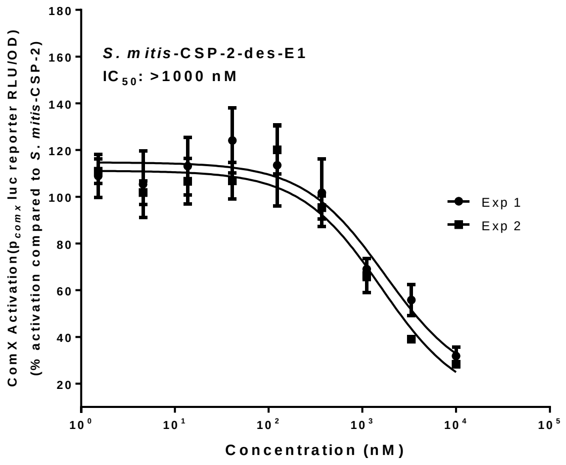

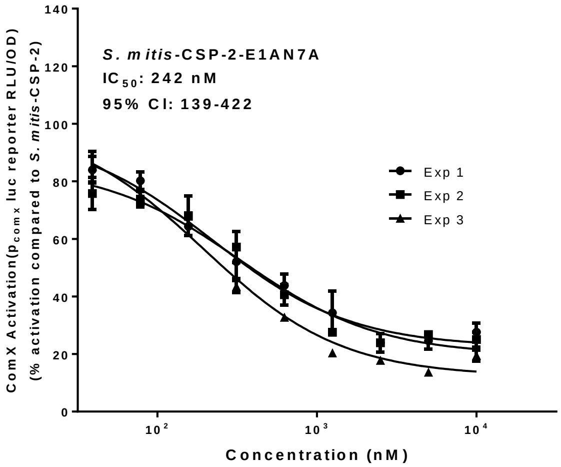
S. mitis-CSP-2-E1AN7A
IC50: 242 nM
95% CI: 139-422
Exp 1
Exp 2
Exp 3
ComX Activation(pcomx luc reporter RLU/OD)
(% activation compared to S. mitis-CSP-2)
Concentration (nM)

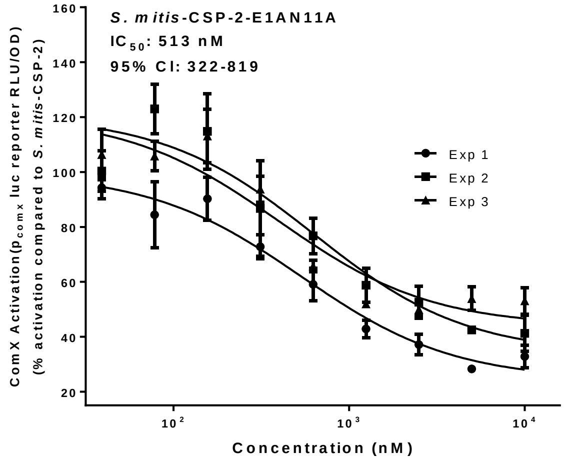
S. mitis-CSP-2-E1AN11A
IC50: 513 nM
95% CI: 322-819
Exp 1
Exp 2
Exp 3
ComX Activation(pcomx luc reporter RLU/OD)
(% activation compared to S. mitis-CSP-2)
Concentration (nM)

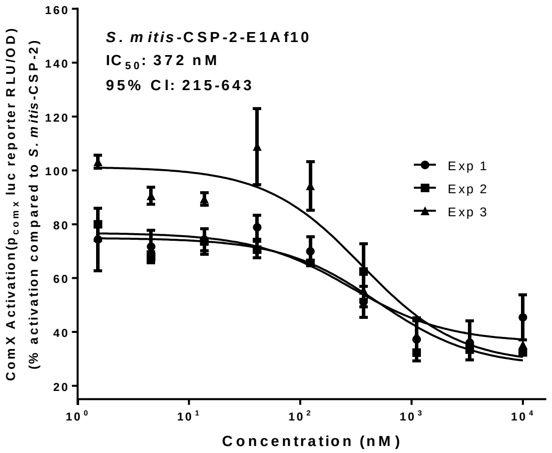
S. mitis-CSP-2-E1Af10
IC50: 372 nM
95% CI: 215-643
Exp 1
Exp 2
Exp 3
ComX Activation(pcomx luc reporter RLU/OD)
(% activation compared to S. mitis-CSP-2)
Concentration (nM)

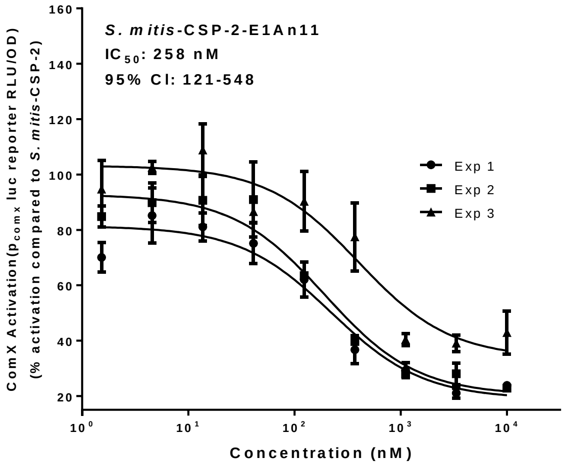
S. mitis-CSP-2-E1An11
IC50: 258 nM
95% CI: 121-548
Exp 1
Exp 2
Exp 3
ComX Activation(pcomx luc reporter RLU/OD)
(% activation compared to S. mitis-CSP-2)
Concentration (nM)

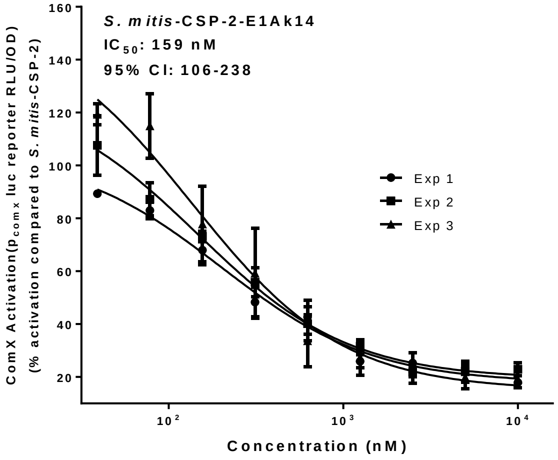
S. mitis-CSP-2-E1Ak14
IC50: 159 nM
95% CI: 106-238
Exp 1
Exp 2
Exp 3
ComX Activation(pcomx luc reporter RLU/OD)
(% activation compared to S. mitis-CSP-2)
Concentration (nM)

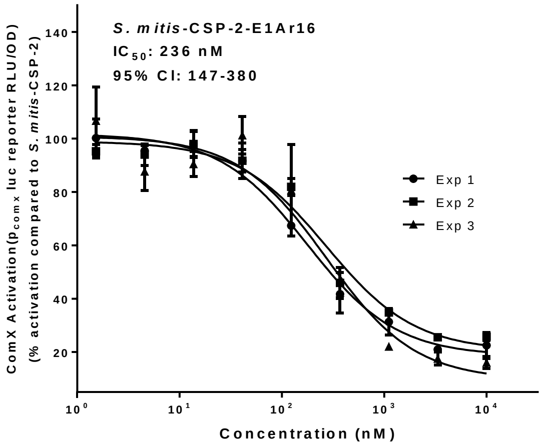
S. mitis-CSP-2-E1Ar16
IC50: 236 nM
95% CI: 147-380
Exp 1
Exp 2
Exp 3
ComX Activation(pcomx luc reporter RLU/OD)
(% activation compared to S. mitis-CSP-2)
Concentration (nM)

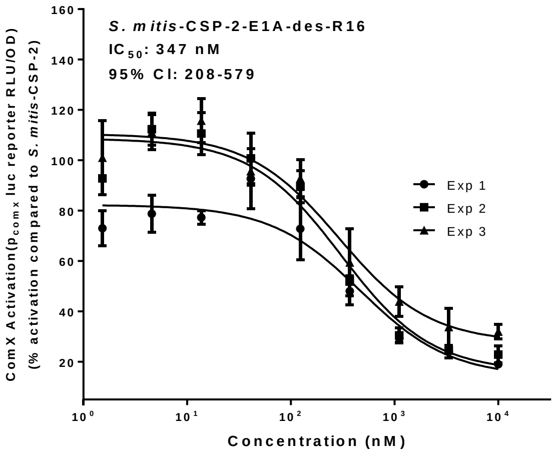
S. mitis-CSP-2-E1A-des-R16
IC50: 347 nM
95% CI: 208-579
Exp 1
Exp 2
Exp 3
ComX Activation(pcomx luc reporter RLU/OD)
(% activation compared to S. mitis-CSP-2)
Concentration (nM)

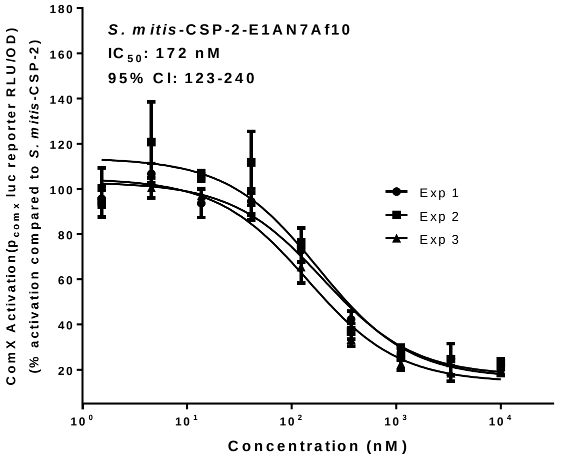
S. mitis-CSP-2-E1AN7Af10
IC50: 172 nM
95% CI: 123-240
Exp 1
Exp 2
Exp 3
ComX Activation(pcomx luc reporter RLU/OD)
(% activation compared to S. mitis-CSP-2)
Concentration (nM)

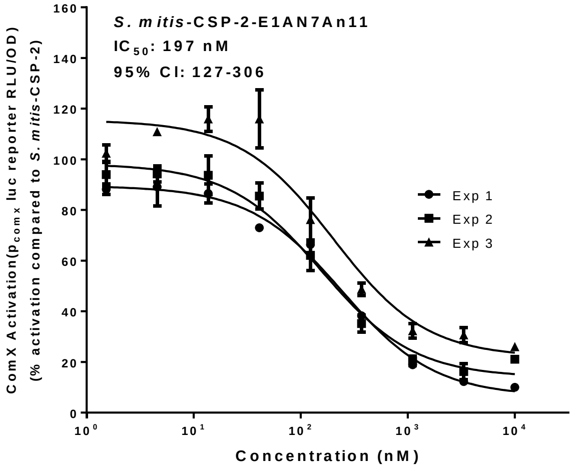
S. mitis-CSP-2-E1AN7An11
IC50: 197 nM
95% CI: 127-306
Exp 1
Exp 2
Exp 3
ComX Activation(pcomx luc reporter RLU/OD)
(% activation compared to S. mitis-CSP-2)
Concentration (nM)

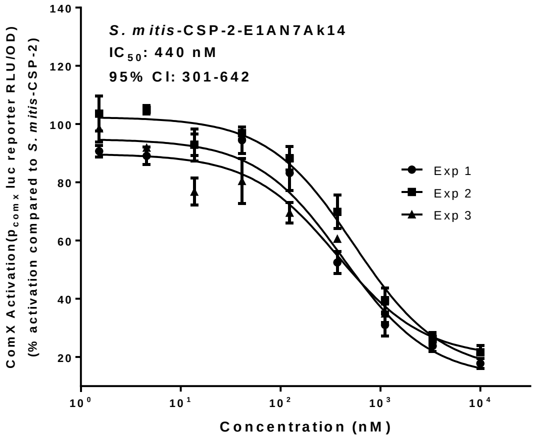
S. mitis-CSP-2-E1AN7Ak14
IC50: 440 nM
95% CI: 301-642
Exp 1
Exp 2
Exp 3
ComX Activation(pcomx luc reporter RLU/OD)
(% activation compared to S. mitis-CSP-2)
Concentration (nM)

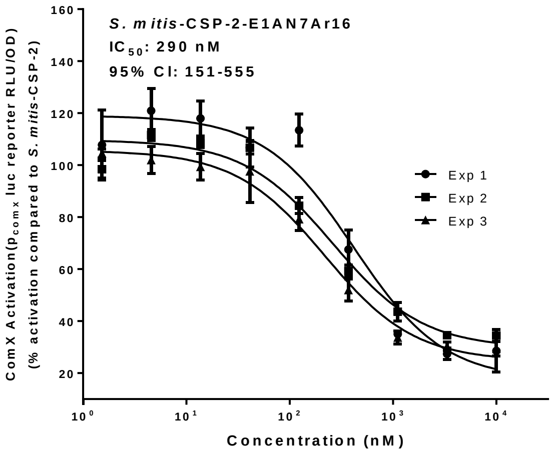
S. mitis-CSP-2-E1AN7Ar16
IC50: 290 nM
95% CI: 151-555
Exp 1
Exp 2
Exp 3
ComX Activation(pcomx luc reporter RLU/OD)
(% activation compared to S. mitis-CSP-2)
Concentration (nM)

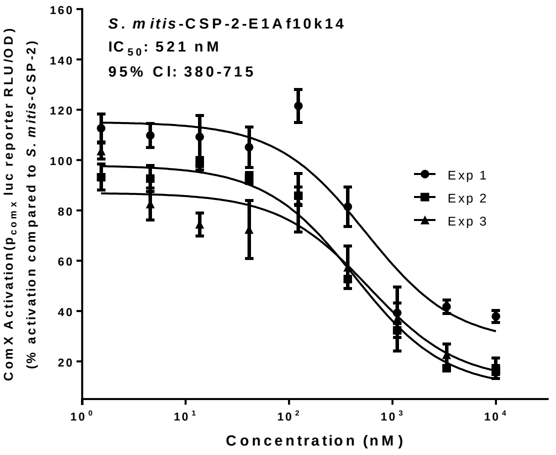
S. mitis-CSP-2-E1Af10k14
IC50: 521 nM
95% CI: 380-715
Exp 1
Exp 2
Exp 3
ComX Activation(pcomx luc reporter RLU/OD)
(% activation compared to S. mitis-CSP-2)
Concentration (nM)

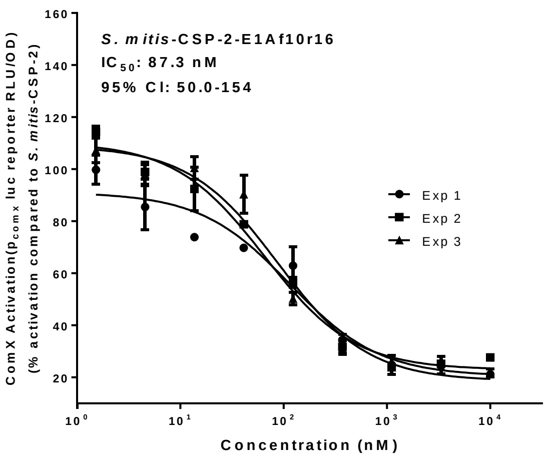
S. mitis-CSP-2-E1Af10r16
IC50: 87.3 nM
95% CI: 50.0-154
Exp 1
Exp 2
Exp 3
ComX Activation(pcomx luc reporter RLU/OD)
(% activation compared to S. mitis-CSP-2)
Concentration (nM)

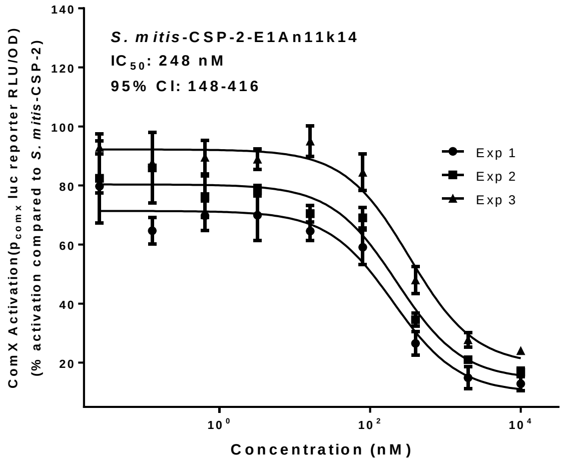
S. mitis-CSP-2-E1An11k14
IC50: 248 nM
95% CI: 148-416
Exp 1
Exp 2
Exp 3
ComX Activation(pcomx luc reporter RLU/OD)
(% activation compared to S. mitis-CSP-2)
Concentration (nM)

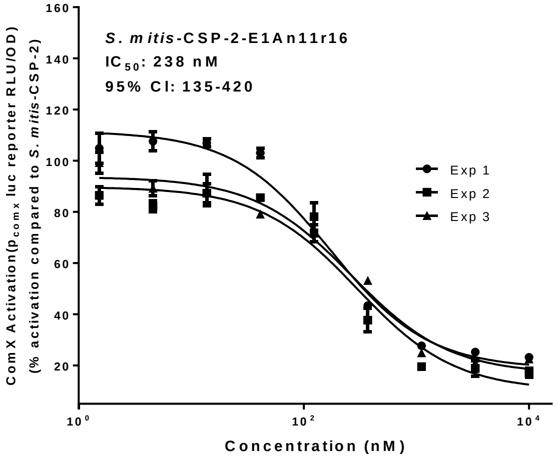
S. mitis-CSP-2-E1An11r16
IC50: 238 nM
95% CI: 135-420
Exp 1
Exp 2
Exp 3
ComX Activation(pcomx luc reporter RLU/OD)
(% activation compared to S. mitis-CSP-2)
Concentration (nM)

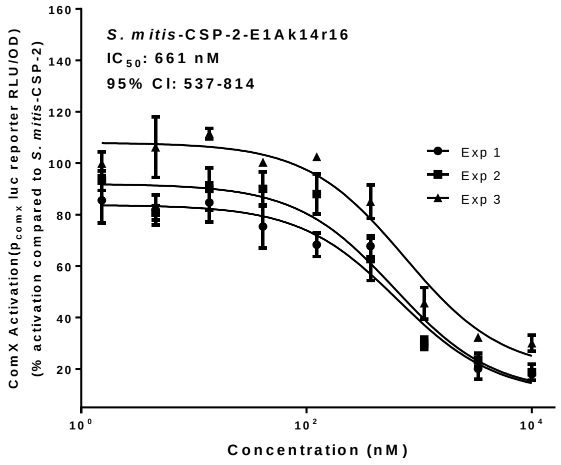
S. mitis-CSP-2-E1Ak14r16
IC50: 661 nM
95% CI: 537-814
Exp 1
Exp 2
Exp 3
ComX Activation(pcomx luc reporter RLU/OD)
(% activation compared to S. mitis-CSP-2)
Concentration (nM)

Circular dichroism (CD) spectra

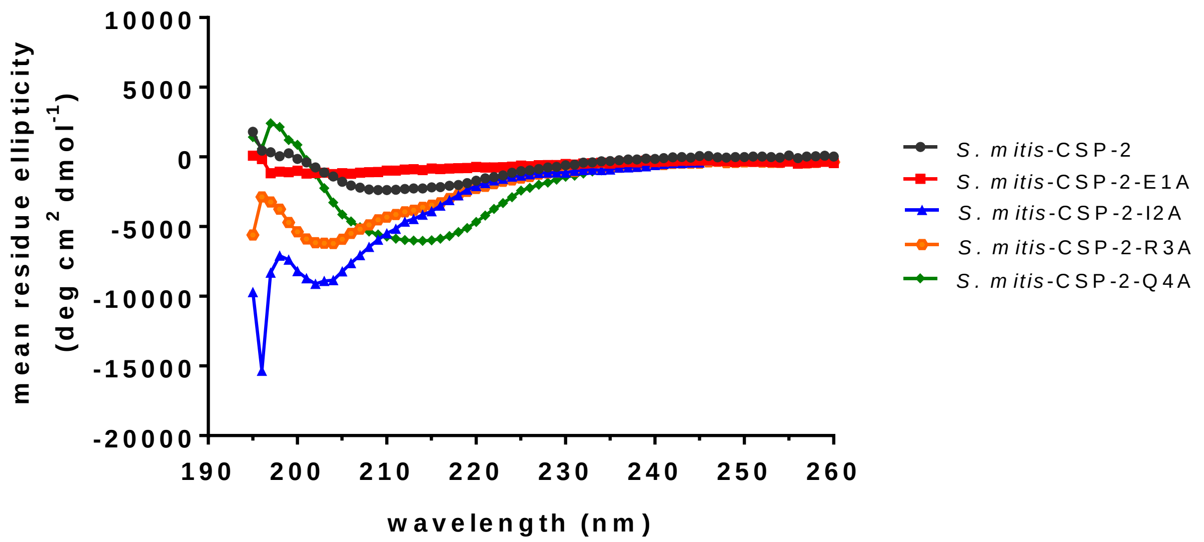

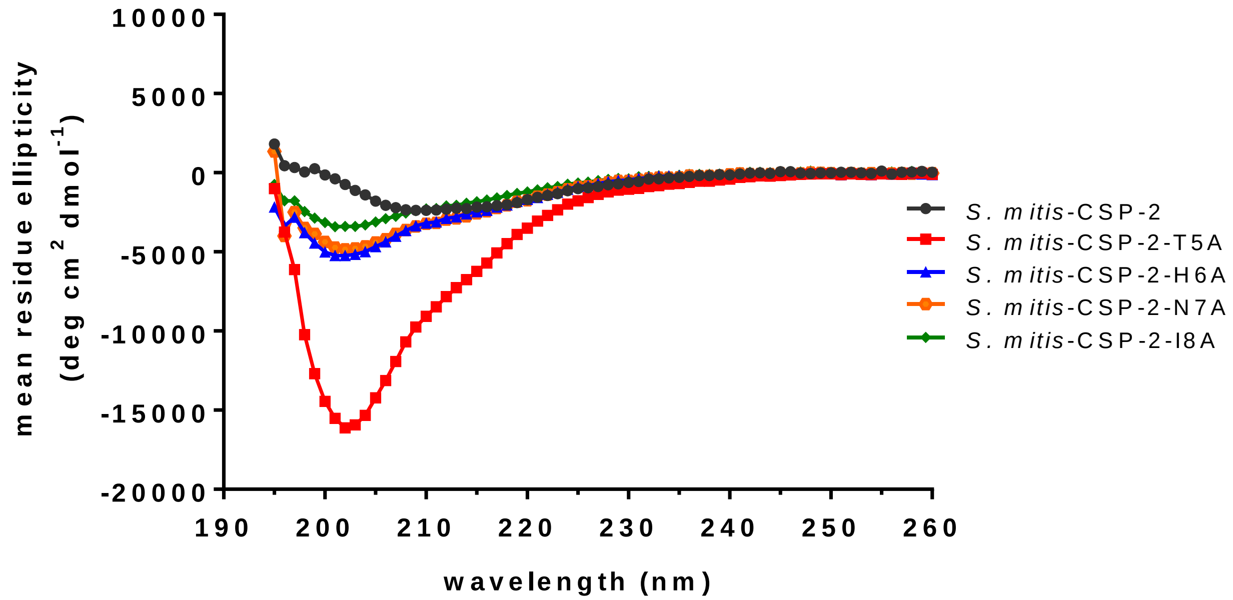

Figure S-14. CD spectra of the *S. mitis*-CSP-2 alanine scan library in aqueous solution (PBS, pH 7.4). All the measurements were performed with a peptide concentration of 200 µM. *S. mitis*-CSP-2 was added as a control. Most of the alanine screen analogs were unstructured exhibiting a random coil pattern, with the exception of *S. mitis*-CSP-2-Q4A that exhibited some β-sheet pattern.

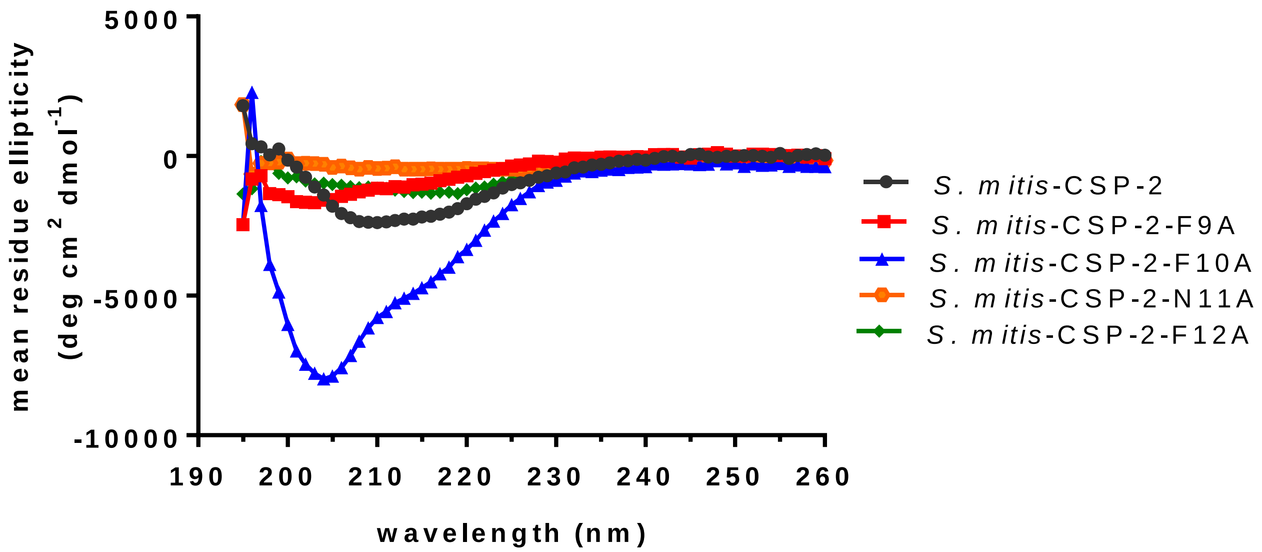

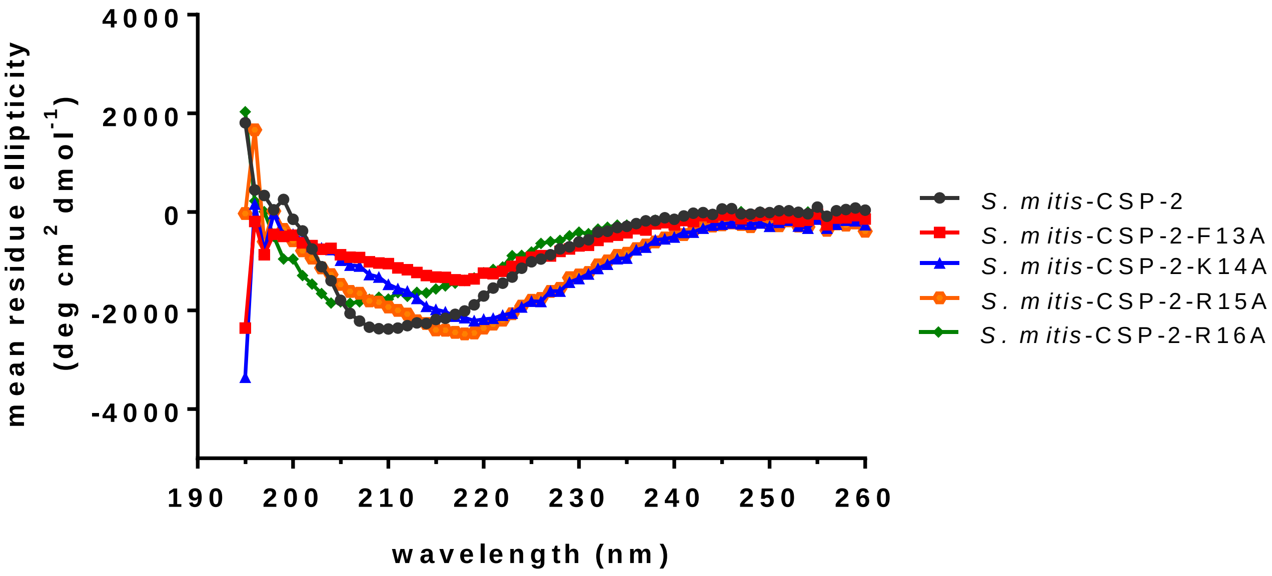

Figure S-15. CD spectra of the *S. mitis*-CSP-2 alanine scan library in aqueous solution (PBS, pH 7.4). All the measurements were performed with a peptide concentration of 200 µM. *S. mitis*-CSP-2 was added as a control. Most of the alanine screen analogs were unstructured exhibiting a random coil pattern, with the exception of *S. mitis*-CSP-2-F13A, *S. mitis*-CSP-2-K14A, and *S. mitis*-CSP-2-R15A that exhibited some β-sheet pattern.

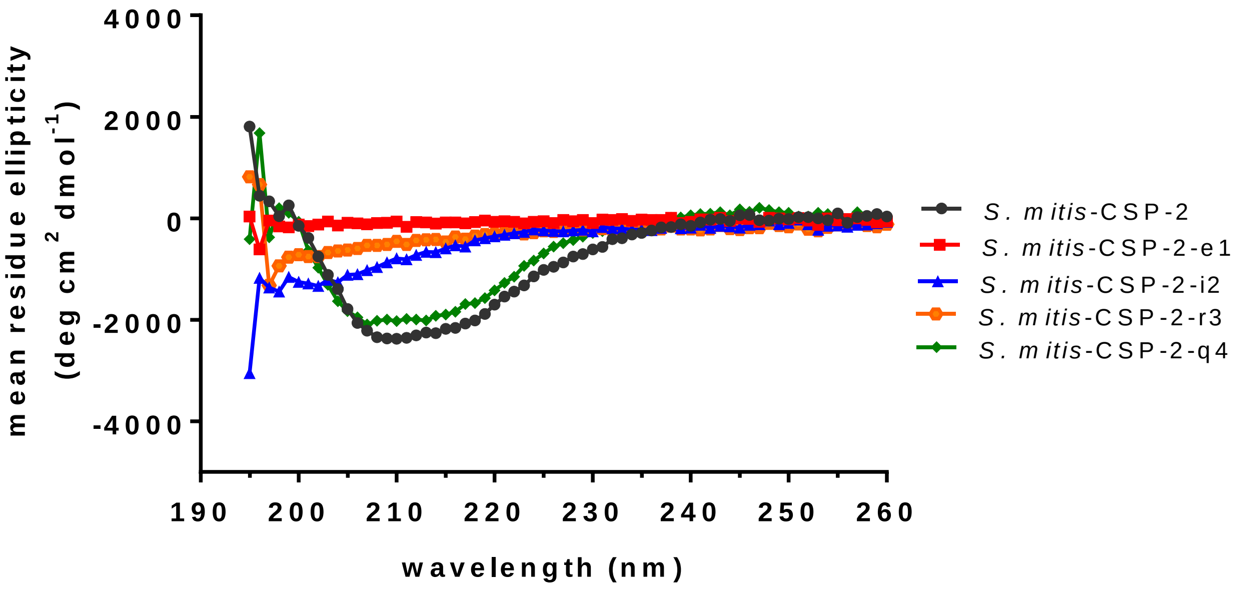

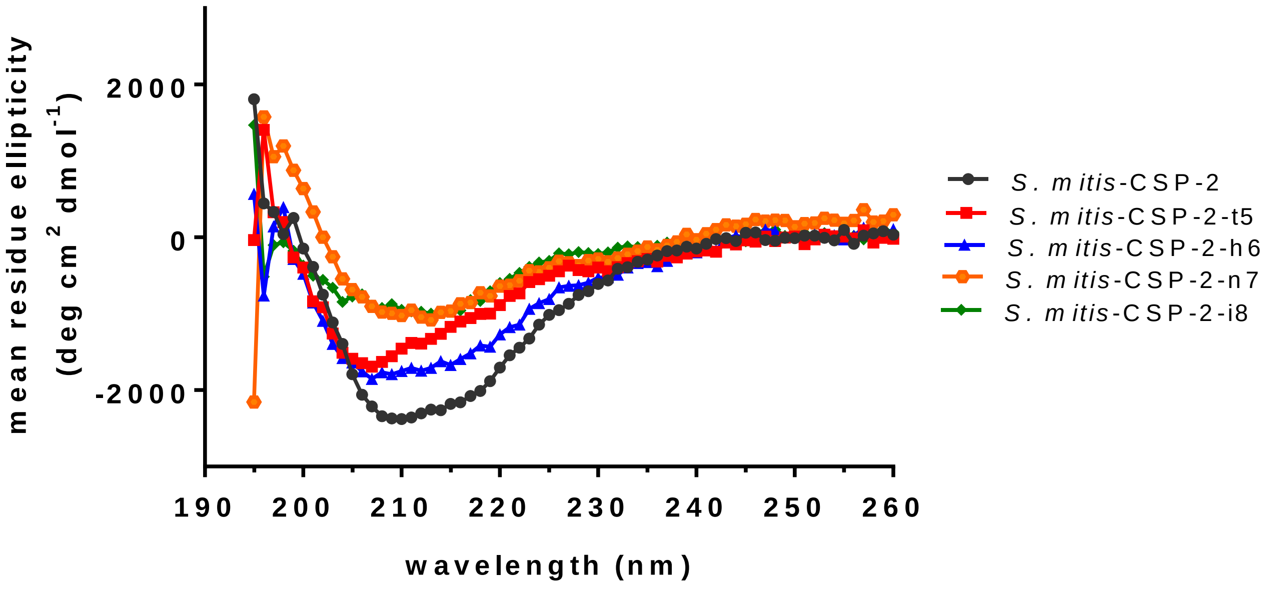

Figure S-16. CD spectra of the *S. mitis*-CSP-2 D-amino acid scan library in aqueous solution (PBS, pH 7.4). All the measurements were performed with a peptide concentration of 200 µM. *S. mitis*-CSP-2 was added as a control. All of the analogs were unstructured exhibiting a random coil pattern.

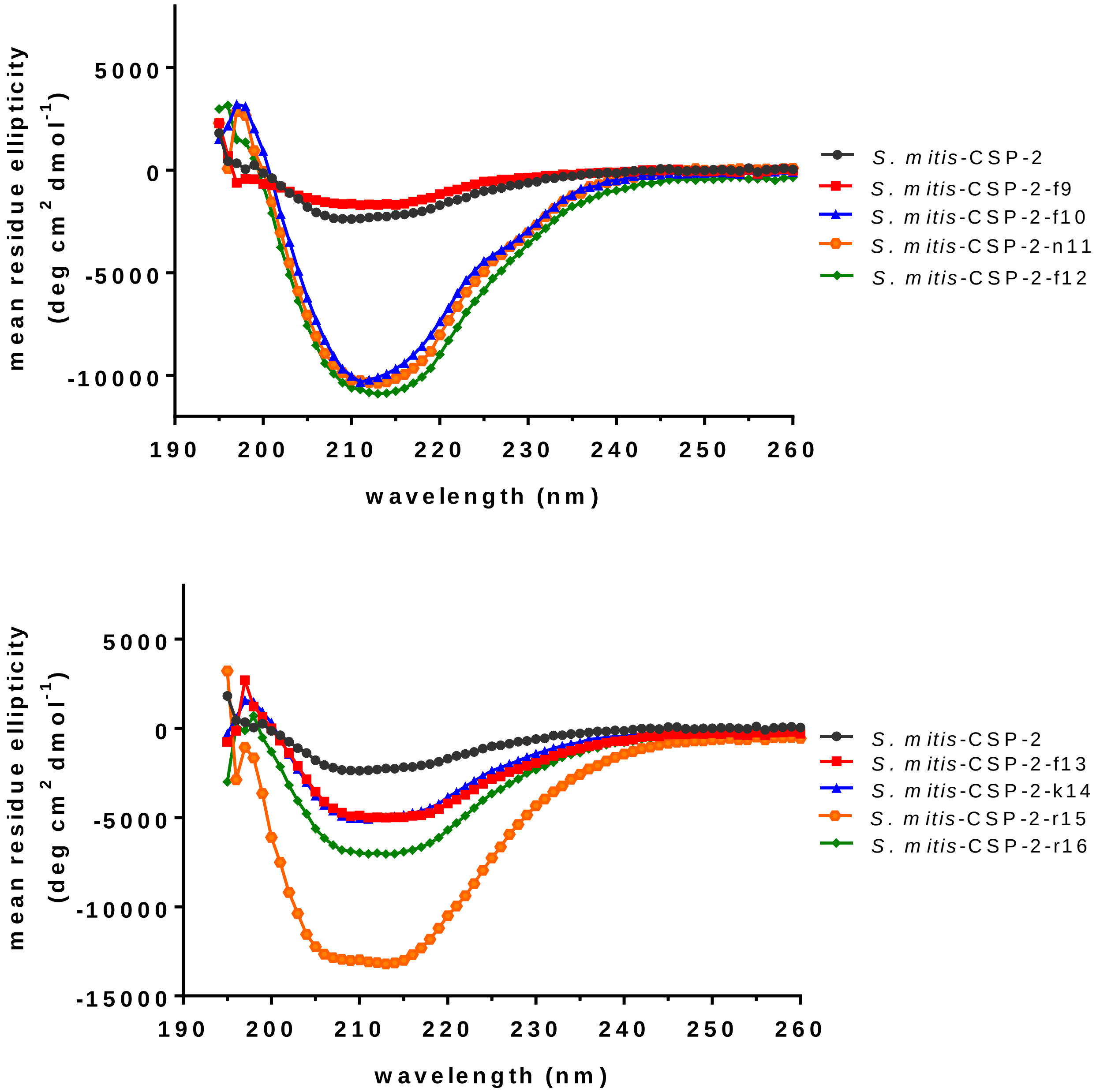

Figure S-17. CD spectra of the *S. mitis*-CSP-2 D-amino acid scan library in aqueous solution (PBS, pH 7.4). All the measurements were performed with a peptide concentration of 200 µM. *S. mitis*-CSP-2 was added as a control. Most of the D-amino acid scan analogs were unstructured exhibiting a random coil pattern, with the exception of *S. mitis*-CSP-2-f10, *S. mitis*-CSP-2-n11, and *S. mitis*-CSP-2-f12 that exhibited some β-sheet pattern.

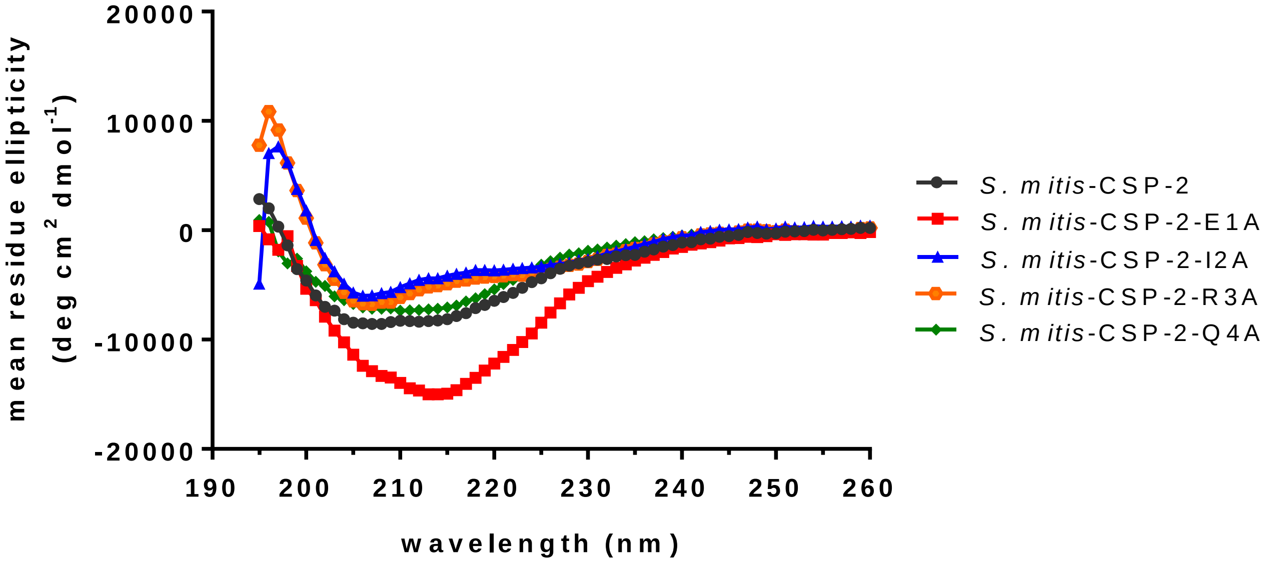

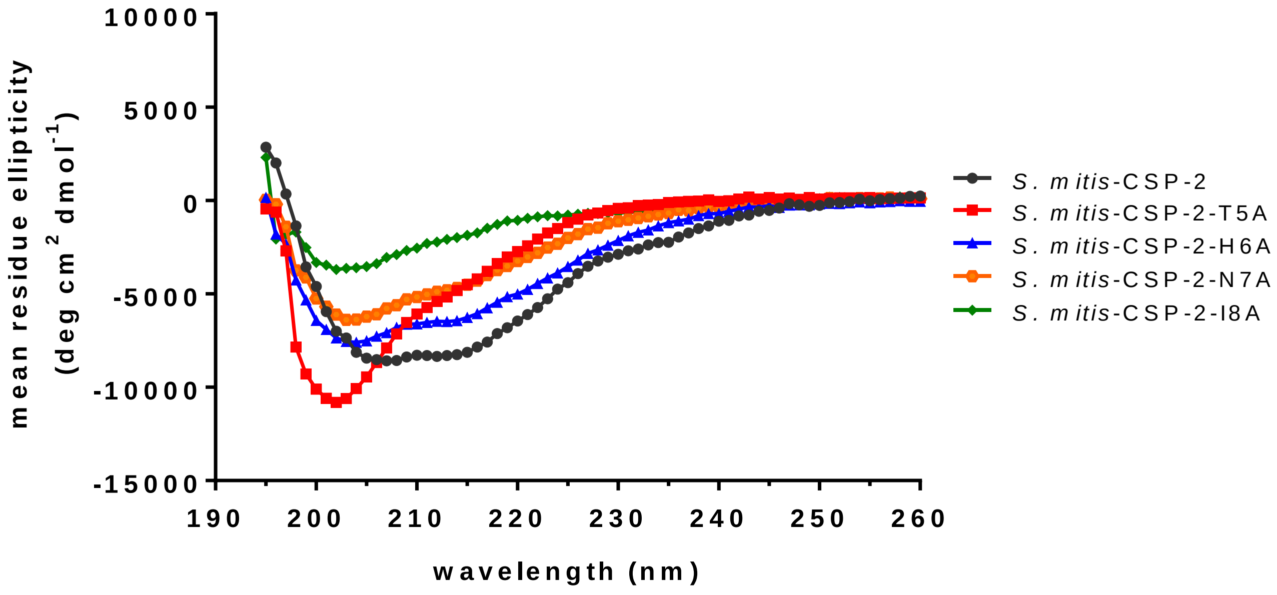

Figure S-18. CD spectra of the *S. mitis*-CSP-2 alanine scan library in membrane mimicking conditions (20% TFE: 80% PBS, pH 7.4). All the measurements were performed with a peptide concentration of 200 µM. *S. mitis*-CSP-2 was added as a control. Most of the alanine screen analogs were unstructured exhibiting a random coil pattern, with the exception of *S. mitis*-CSP-2-E1A that exhibited some β-sheet pattern.

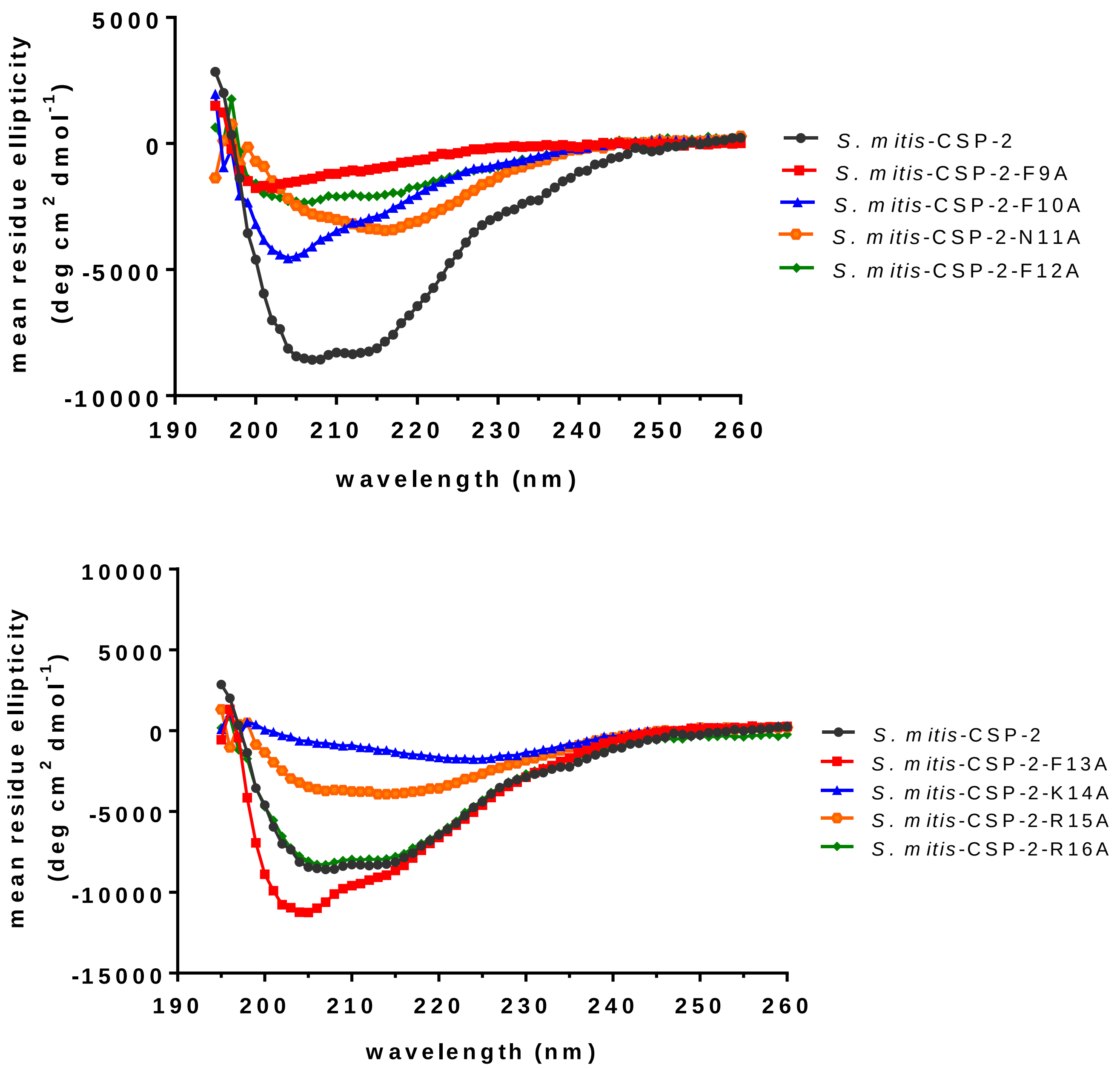

Figure S-19. CD spectra of the *S. mitis*-CSP-2 alanine scan library in membrane mimicking conditions (20% TFE: 80% PBS, pH 7.4). All the measurements were performed with a peptide concentration of 200 μM. *S. mitis*-CSP-2 was added as a control. Most of the alanine screen analogs were unstructured exhibiting a random coil pattern, with the exception of *S. mitis*-CSP-2-N11A that exhibited some β-sheet pattern.

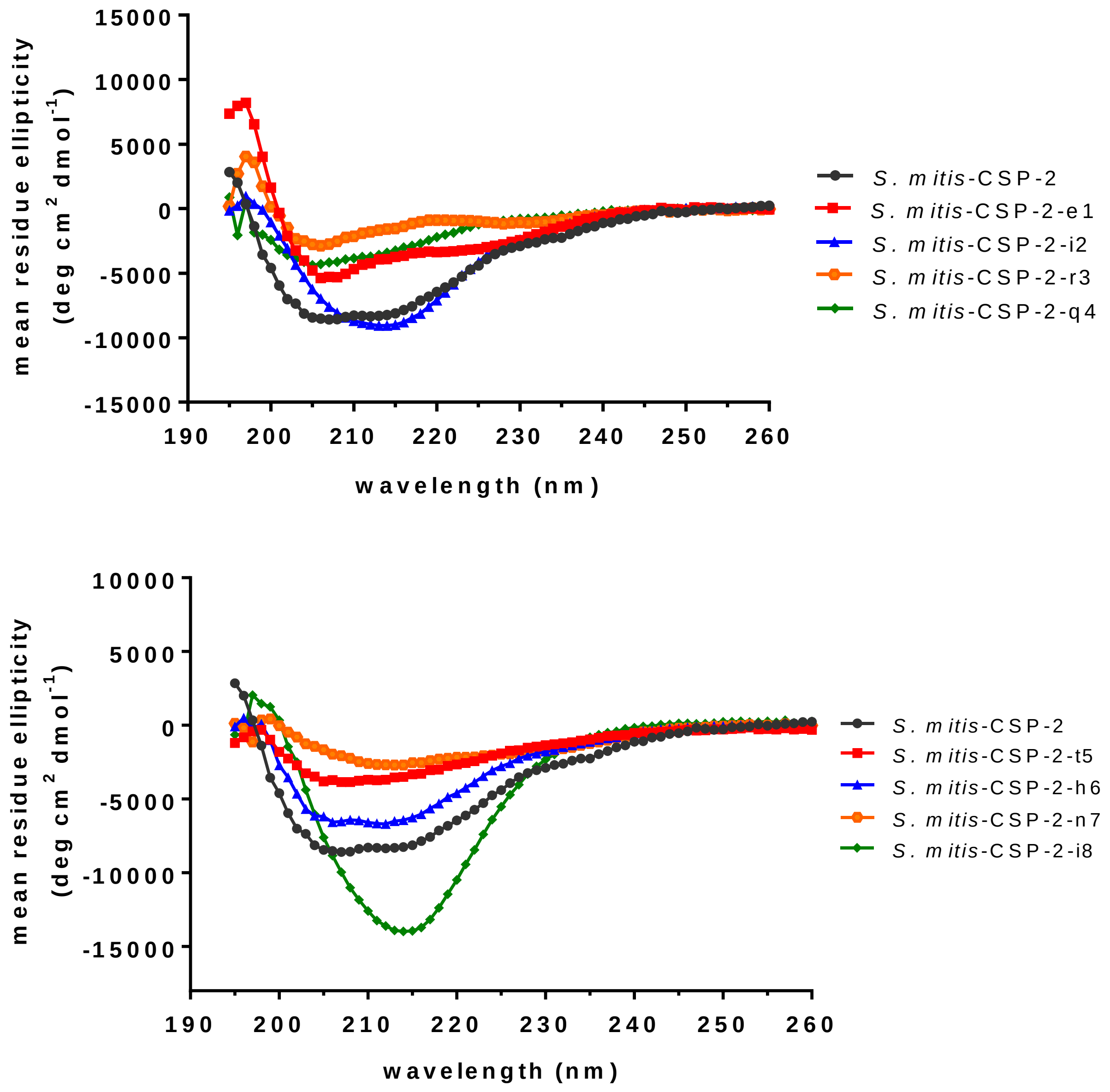

Figure S-20. CD spectra of the *S. mitis*-CSP-2 D-amino acid scan library in membrane mimicking conditions (20% TFE: 80% PBS, pH 7.4). All the measurements were performed with a peptide concentration of 200 µM. *S. mitis*-CSP-2 was added as a control. Most of the D-amino acid scan analogs were unstructured exhibiting a random coil pattern, with the exception of *S. mitis*-CSP-2-i2, and *S. mitis*-CSP-2-i8 that exhibited some β-sheet pattern.

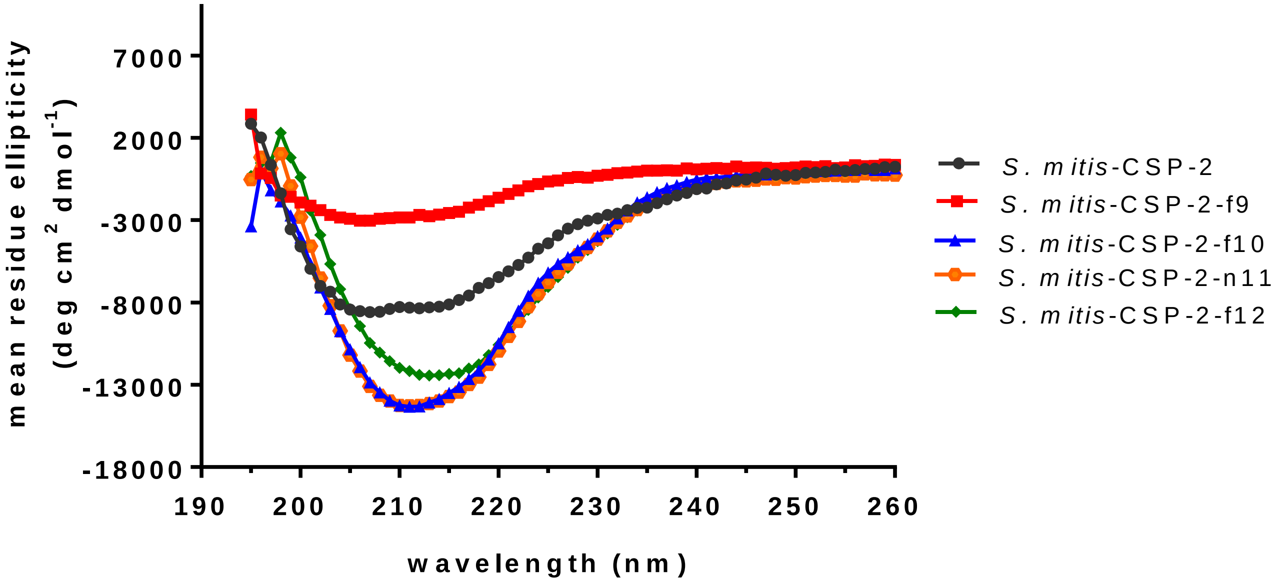

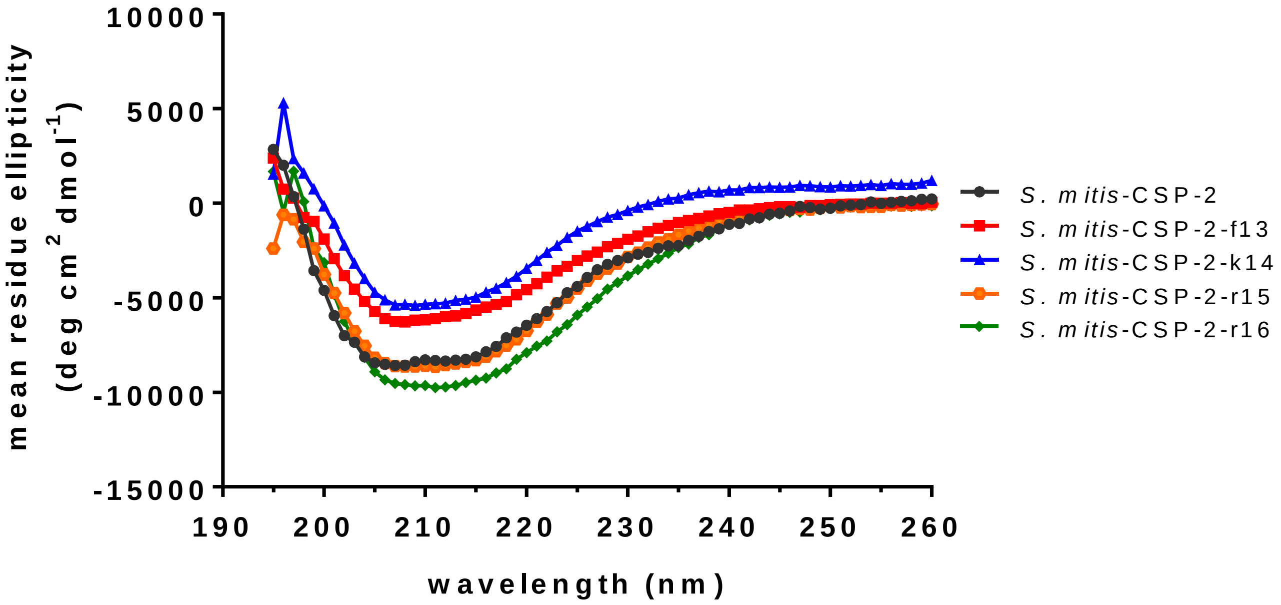

Figure S-21. CD spectra of the *S. mitis*-CSP-2 D-amino acid scan library in membrane mimicking conditions (20% TFE: 80% PBS, pH 7.4). All the measurements were performed with a peptide concentration of 200 µM. *S. mitis*-CSP-2 was added as a control. Most of the D-amino acid scan analogs were unstructured exhibiting a random coil pattern, with the exception of *S. mitis*-CSP-2-f10, *S. mitis*-CSP-2-n11, and *S. mitis*-CSP-2-f12 that exhibited some β-sheet pattern.

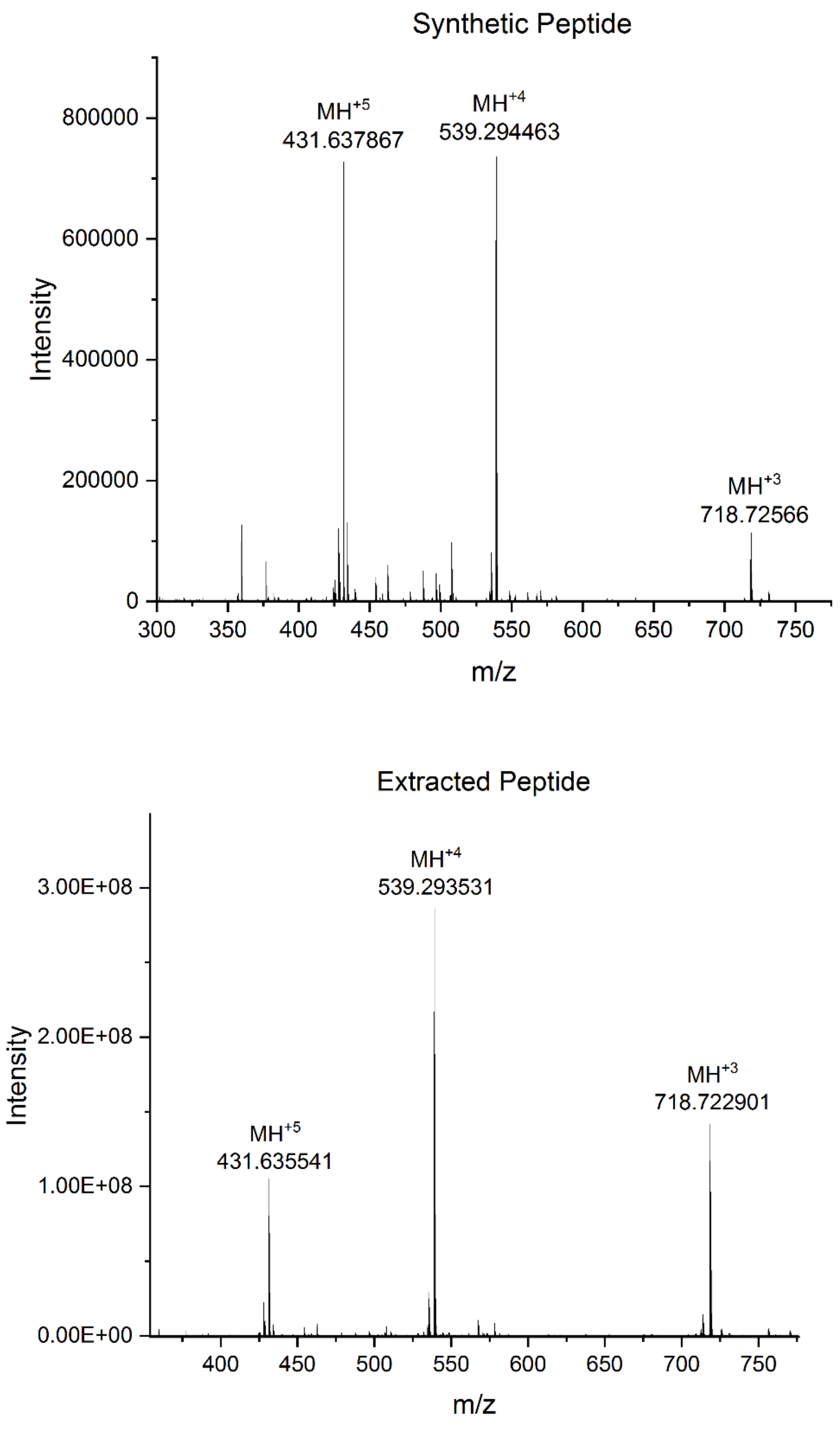

Figure S-22. Exact mass spectra of synthetic and extracted *S. mitis*-CSP-2.

A.

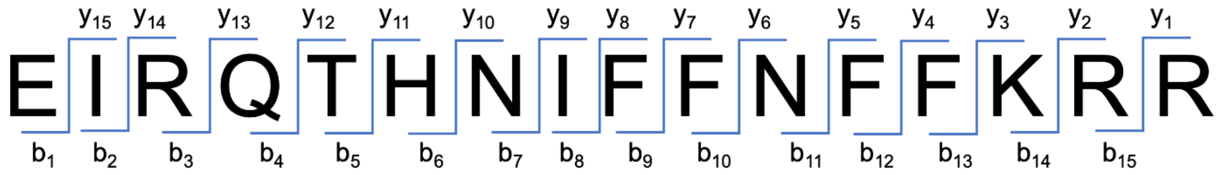

B.

Extracted Peptide			
**m/z**	**Y-Fragment**	**m/z**	**B-Fragment**
675.372021	y_{15}^{+3}	243.13322	b_2
637.677296	y_{14}^{+3}	399.234413	b_3
877.96467	y_{13}^{+2}	527.293264	b_4
813.933694	y_{12}^{+2}	628.341333	b_5
763.411276	y_{11}^{+2}	765.399488	b_6
694.882832	y_{10}^{+2}	879.442599	b_7
637.860666	y_9^{+2}	992.523141	b_8
581.318775	y_8^{+2}	570.301192	b_9^{+2}
507.783843	y_7^{+2}	643.834733	b_{10}^{+2}
867.494174	y_6	700.855728	b_{11}^{+2}
753.452229	y_5	774.393209	b_{12}^{+2}
606.382597	y_4	ND	ND
459.313944	y_3	452.237913	b_{14}-NH_3^{+4}
331.219297	y_2	990.023572	b_{15}^{+2}

Figure S-23. MS/MS analysis of the extracted *S. mitis*-CSP-2. (A) *S. mitis*-CSP-2 sequence showing the MS/MS fragments patterns. (B) Complete table of the fragments detected through the MS/MS analysis of the extracted *S. mitis*-CSP-2. ND, Not detected

A.

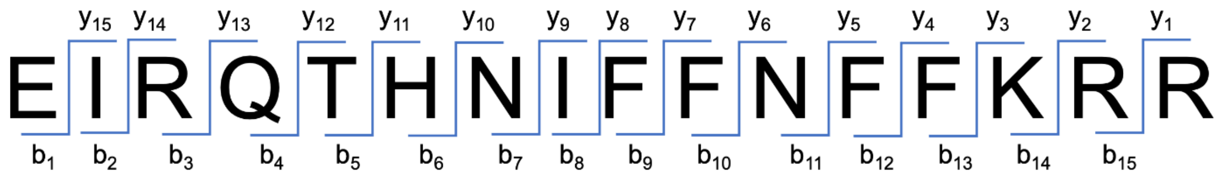

B.

Synthetic Peptide			
**m/z**	**Y-Fragment**	**m/z**	**B-Fragment**
675.373252	y_{15}^{+3}	225.122826	b_2-H_2O
637.678024	y_{14}^{+3}	399.233677	b_3
877.96259	y_{13}^{+2}	527.292339	b_4
813.934907	y_{12}^{+2}	628.340269	b_5
763.410042	y_{11}^{+2}	765.401093	b_6
694.881683	y_{10}^{+2}	879.441229	b_7
637.861752	y_9^{+2}	992.525846	b_8
1161.63507	y_8	570.300207	b_9^{+2}
1014.56025	y_7	643.833649	b_{10}^{+2}
867.492818	y_6	691.850776	b_{11}-H_2O^{+2}
753.451007	y_5	ND	ND
606.383567	y_4	ND	ND
459.314439	y_3	ND	ND
331.219479	y_2	491.25873	b_{15}-NH_3^{+4}
175.11883	y_1	ND	ND

Figure S-24. MS/MS analysis of the synthetic *S. mitis*-CSP-2. (A) *S. mitis*-CSP-2 sequence showing the MS/MS fragments patterns (this is a reuse of figure S-23A). (B) Complete table of the fragments detected through the MS/MS analysis of the synthetic *S. mitis*-CSP-2. ND, Not detected

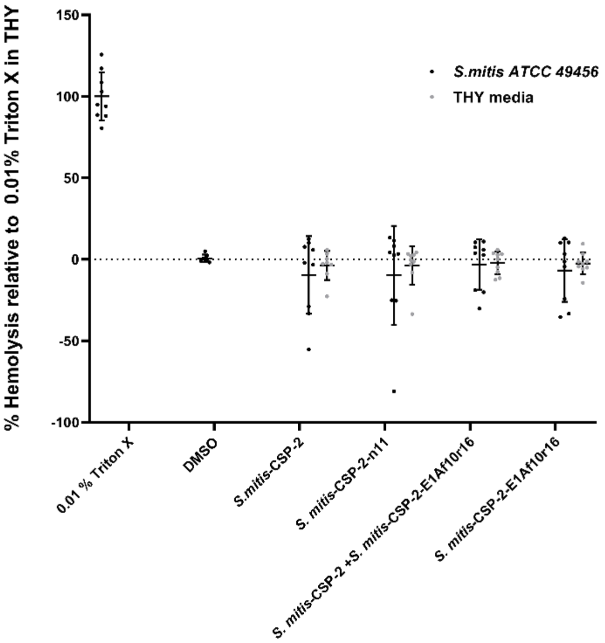

Figure S-25. Hemolytic activity of *S. mitis*-CSP-2-derived QS modulators against defibrinated rabbit red blood cells in THY media (grey) or in the presence of *S. mitis* ATCC 49456 (black). The mean (±S.D.) was as follows: 0.01% Triton X 100% ± 15%; DMSO 0.62% ±2.3%; *S. mitis*-CSP-2 in THY media -3.8% ±8.9%; *S. mitis*-CSP-2-n11 in THY media -3.8% ±12%; *S. mitis*-CSP-2 + *S. mitis*-CSP-2-E1Af10r16 in THY media -2.3% ±7.0%; *S. mitis*-CSP-2-E1Af10r16 in THY media -2.5% ±6.6%; *S. mitis*-CSP-2 with *S. mitis* ATCC 49456 -9.6% ±24%; *S. mitis*-CSP-2-n11 with *S. mitis* ATCC 49456 -9.8% ±30%; *S. mitis*-CSP-2 + *S. mitis*-CSP-2-E1Af10r16 with *S. mitis* ATCC 49456 -3.1% ±16%; and *S. mitis*-CSP-2-E1Af10r16 with *S. mitis* ATCC 49456 -6.9% ±19%. Both *S. mitis* and the lead *S. mitis*-CSP-2-based QS modulators exhibited no apparent cytotoxicity. Experiments were performed in triplicate on three separate days.

Reference:

1 Milly, T. A. & Tal-Gan, Y. Biological evaluation of native streptococcal competence stimulating peptides reveals potential crosstalk between Streptococcus mitis and Streptococcus pneumoniae and a new scaffold for the development of S. pneumoniae quorum sensing modulators. *RSC Chem Biol* **1**, 60-67 (2020).
